# Genetic heterogeneity and homogeneity among orofacial cleft subtypes: genome-wide association studies in the cleft collective

**DOI:** 10.1093/hmg/ddaf131

**Published:** 2025-10-11

**Authors:** Kyle Dack, Kerstin U Ludwig, Evie Stergiakouli, Jonathan Sandy, Sethlina Aryee, George Davey Smith, Amy Davies, Yvonne Wren, Gemma C Sharp, Kerry Humphries, Elisabeth Mangold, Lucy Goudswaard, Karen Ho, Tom Dudding, Sarah J Lewis

**Affiliations:** Population Health Sciences, Bristol Medical School, University of Bristol, Oakfield House, Oakfield Grove, Bristol, BS8 2BN, United Kingdom; MRC-Integrative Epidemiology Unit, University of Bristol, Oakfield House, Oakfield Grove, Bristol, BS8 2BN, United Kingdom; Institute for Human Genetics, University of Bonn, Karlrobert- Kreitenstr. 13. 53115, Bonn, Germany; Population Health Sciences, Bristol Medical School, University of Bristol, Oakfield House, Oakfield Grove, Bristol, BS8 2BN, United Kingdom; MRC-Integrative Epidemiology Unit, University of Bristol, Oakfield House, Oakfield Grove, Bristol, BS8 2BN, United Kingdom; Bristol Dental School, University of Bristol, 1 Trinity Quay, Avon Street, Bristol, BS2 0PT, United Kingdom; Institute for Social and Economic Research, University of Essex, Wivenhoe Park, Colchester, Essex, CO4 3SQ, United Kingdom; Population Health Sciences, Bristol Medical School, University of Bristol, Oakfield House, Oakfield Grove, Bristol, BS8 2BN, United Kingdom; MRC-Integrative Epidemiology Unit, University of Bristol, Oakfield House, Oakfield Grove, Bristol, BS8 2BN, United Kingdom; Bristol Dental School, University of Bristol, 1 Trinity Quay, Avon Street, Bristol, BS2 0PT, United Kingdom; Bristol Dental School, University of Bristol, 1 Trinity Quay, Avon Street, Bristol, BS2 0PT, United Kingdom; School of Psychology, University of Exeter, Washington Singer Building, Perry Road, Exeter, EX4 4QG, United Kingdom; Bristol Dental School, University of Bristol, 1 Trinity Quay, Avon Street, Bristol, BS2 0PT, United Kingdom; Institute for Human Genetics, University of Bonn, Karlrobert- Kreitenstr. 13. 53115, Bonn, Germany; Population Health Sciences, Bristol Medical School, University of Bristol, Oakfield House, Oakfield Grove, Bristol, BS8 2BN, United Kingdom; MRC-Integrative Epidemiology Unit, University of Bristol, Oakfield House, Oakfield Grove, Bristol, BS8 2BN, United Kingdom; Population Health Sciences, Bristol Medical School, University of Bristol, Oakfield House, Oakfield Grove, Bristol, BS8 2BN, United Kingdom; Bristol Dental School, University of Bristol, 1 Trinity Quay, Avon Street, Bristol, BS2 0PT, United Kingdom; Population Health Sciences, Bristol Medical School, University of Bristol, Oakfield House, Oakfield Grove, Bristol, BS8 2BN, United Kingdom; MRC-Integrative Epidemiology Unit, University of Bristol, Oakfield House, Oakfield Grove, Bristol, BS8 2BN, United Kingdom

**Keywords:** orofacial cleft, GWAS, heterogeneity, combined clefts, Pierre Robin sequence

## Abstract

Several genome wide association studies (GWASs) of orofacial cleft have been conducted. However only a few such studies to date have combined all cleft cases, focused on subtypes other than non-syndromic cleft lip with/without cleft palate, or investigated subtype heterogeneity. We conducted a GWAS of orofacial clefts within 2268 cases from the Cleft Collective and 7913 population-based controls; we performed analyses of all orofacial clefts, plus 7 subgroups. We replicated our findings in a meta-analysis of independent samples and investigated patterns of correlation across subgroups. We identified 27 regions at genome-wide significance, 8 of which were novel. We also conducted the first GWAS of Pierre Robin Sequence, despite the small sample size (n cases = 237), we found one genome wide significant SNP (*P* < 5 × 10^−8^), and another 21 suggestive associations (*P* < 10^−5^). Novel loci include those mapping to *LHX8* and *TSBP1* (combined clefts), *ARHGEF18* and *ARHGEF19* (cleft lip with/without palate), *FBN2* (cleft lip only), *SLC35B3* (cleft palate only), *CASC20* (Pierre Robin Sequence) and *CHRM2* (non-syndromic cleft palate only). Several novel hits were in regions previously associated with facial morphology in GWAS or were in regions involved in key developmental processes, including neural crest cell migration and craniofacial development. We identified genetic loci with similar effects across all subgroups and some loci which were subtype specific, we also identified 3 loci with opposing effects on cleft lip and Pierre Robin sequence. Our findings highlight the merit of including all orofacial cleft subtypes in GWAS studies and investigating heterogeneity of effects across subtypes.

## Introduction

Orofacial clefts are among the most common congenital birth anomalies, occurring in approximately 1 in 700 births globally [1]. These anomalies can manifest as cleft lip only, cleft palate only, or a combination of both cleft lip and palate [2]. Orofacial clefts occur in syndromic or non-syndromic forms, with non-syndromic clefts being more prevalent than syndromic [2, 3]. Syndromic clefts typically occur alongside other anomalies or health issues and are usually caused by a rare single gene or chromosomal mutation, although the penetrance of these mutations can differ between individuals resulting in heterogeneity of the phenotype [4]. In contrast, non-syndromic clefts follow a multifactorial aetiology and, accordingly, are influenced by both genetic and environmental factors [5].

Pierre Robin sequence (PRS) presents as a triad of anomalies arising from a single underlying cause. The three primary characteristics include micrognathia (a small and retrusive mandible), leading to glossoptosis (a downwardly displaced or retracted tongue), which subsequently obstructs the upper airway and causes breathing difficulties. Additionally, a wide, U-shaped cleft palate is frequently present [6]. Rare loss of function mutations in the *SOX9* gene on chromosome 17 are established risk factors for PRS, along with mutations in other genes including; *COL11A1, COL11A2, COL2A1,* which cause a syndromic form of PRS -Stickler’s syndrome [7, 8], mutations in *SATB2*, which have been shown to cause another syndromic form of PRS [9] and 22q11.2 chromosomal deletions [10]. In addition, it is likely that environmental factors and additional genes also play a role in PRS [10, 11].

Genome wide association studies (GWAS) of orofacial clefts have identified over 50 genetic risk loci associated with non-syndromic cleft lip with or without cleft palate (nsCL/P) but relatively few loci have been identified for cleft palate only (CPO) [12–20]. To our knowledge there haven’t been any GWAS of Pierre Robin Sequence to date.

Syndromic forms of cleft are typically excluded from GWAS as these are thought to add noise to the analysis due to having a monogenic cause. However, recent evidence suggests that the boundaries between syndromic and non-syndromic clefts, as well as between different cleft subtypes, are less distinct than previously assumed [21]. Both rare and common genetic variants appear to contribute to all cleft subtypes [21]. Exome sequencing studies have identified single pathogenic mutations in non-syndromic orofacial cleft families [22]. In addition, common genetic variants have been shown to contribute to the variation of neurodevelopmental conditions which were thought to have a monogenic cause [23], such variants may well contribute to syndromic orofacial clefts.

Furthermore, genetic variants identified at genome-wide significance in one cleft subtype might also influence other subtypes, albeit with smaller effect sizes; if this is the case combining genetic data across subtypes may increase the power to detect new loci. Although previous studies have combined cleft lip only (CLO) with cleft lip and palate (CLP) and cleft palate only (CPO) subtypes, and or investigated genetic heterogeneity among these subtypes most have excluded syndromic cases [24–28]. Notably, only the study by Moreno Uribe et al. [29] included syndromic forms. To date, no study has systematically evaluated the magnitude of SNP effects across multiple cleft subtypes, including Pierre Robin sequence.

The United Kingdom (UK) based Cleft Collective [30], at the University of Bristol, is one of the largest cohort studies of children born with cleft and their families. The aim of this study was to conduct GWASs to identify new genetic variants for all orofacial clefts and its subtypes and to investigate the heterogeneity between subtypes using a case–control design.

## Results

**Figure 1:**
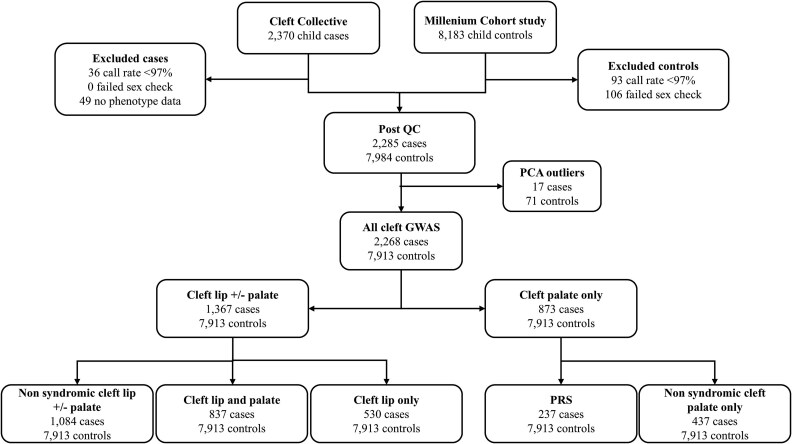
Flow chart showing number of individuals included in our combined cases and subgroup analyses following quality control exclusions.

After applying quality control procedures, we were left with 2268 cases and 7913 controls of European ethnicity for our GWAS analyses. Supplementary Fig. 1 shows that the cases and controls completely overlapped in a scatter plot of principal component (PC) 1 versus PC 2, suggesting that they were genetically similar. Out of the 2268 total orofacial cleft cases included in our study (which formed the ‘all cleft cases group’), we had detailed phenotype information on 2240 individuals and, we grouped these into cleft lip with or without palate (CL/P) (n = 1367) or cleft palate only (CPO)(n-873) subgroups. 283 of our CL/P cases had been diagnosed as having a syndrome, which left a subgroup of 1084 individuals without a known syndrome which were analysed as a subgroup of non-syndromic cleft lip with or without palate (nsCL/P). Our CL/P subgroup further consisted of non-overlapping groups of cleft lip only (CLO) (n = 530) and cleft lip and palate (CLP) (n = 837). Our CPO subgroup was further broken down into those individuals with Pierre Robin sequence (PRS) (n = 237) and non-syndromic cleft palate only cases without PRS (nsCPO) (n = 437). The remaining 160 cleft palate only cases were individuals who had a syndrome but did not have PRS, these were not included in a separate analysis but were included in the CPO analysis. Figure 1 shows the number of individuals excluded at each stage of the QC process and the final numbers of each subtype included in our analyses. Table 1 provides information on the orofacial cleft subgroups investigated.

**Table 1:** Table providing information on how orofacial subtypes were defined and abbreviated.

Cleft subtype	Abbreviation	Description
**Cleft lip with or without cleft palate**	CL/P	Children born with a cleft lip regardless of whether they also had a cleft palate or not and regardless of whether they had a syndrome or not
**non-syndromic cleft lip with or without cleft palate**	nsCL/P	As for CL/P above but excluding any children known to have a syndrome
**Cleft lip with cleft palate**	CLP	Children born with a cleft lip plus a cleft palate whether or not they are known to have a syndrome
**Cleft lip only**	CLO	Children born with a cleft lip but not a cleft palate, includes children with and without a syndrome or sequence.
**Cleft palate only**	CPO	Children born with a cleft palate but not a cleft lip, includes those with and without a syndrome or sequence (e.g. Pierre Robin sequence (PRS))
**non-syndromic cleft palate only**	nsCPO	Children with a cleft palate but not a cleft lip, excluding children with a known syndrome or sequence
**Pierre-Robin Sequence**	PRS	Children who have been diagnosed as have PRS regardless of whether they also have a syndrome or not

Lambda values for GWAS inflation ranged from 1.02 to 1.13 in our GWAS analyses (Supplementary Table S1). We identified 1077 genome-wide-significant SNPs (p < 5 × 10^−8^), which reduced to 27 lead SNPs after clumping (Table 2). Of the regions identified, 19 had been reported in previous orofacial cleft studies (indicated by the GWAS catalogue), while 8 were novel (Table 3). Our strongest association was between a previously identified SNP (rs17242358) on chromosome 8q24 and nsCL/P (*P* = 3.36 × 10^−42^). Supplementary Table S2 shows a summary of the number of genome-wide significant associations by cleft subtype and Supplementary Table S3 shows odds ratios and p-values for our lead SNPs across all cleft subtypes.

**Table 2:** Information on chromosome location and allele frequency for lead GWAS significant SNPs identified and results for the orofacial cleft phenotypes they were most strongly associated with.

Lead SNP	Location (GRCh37)	Chromosomal band	Mapped gene/s	chromosomal range of GWAS sign SNPs	A1	A2	MAF controls	95% credible interval	Lead phenotype	Phenotypes with *P* < 10^−4^	Lead phenotype Odds ratio	P-value	Replicates?
**rs61769781**	**1:16544626**	**1p36.13**	**ARHGEF19**	**16 537 366–1 655 264**	**A**	**G**	**0.11**	**16 514 898–16 633 123**	**nsCL/P**	**All lip phenotypes**	**1.44**	**1.81E-8**	**Yes, *P* = 0.0014**
rs9439714	1:18976489	1p36.13	PAX7^a^ (intron)	18 920 364-19 027 239	C	T	0.35	18 965 061–18 978 372	CL/P	All lip phenotypes	1.45	4.06E-18	Yes, *P* = 1.1E-16
**rs4112328**	**1:75500597**	**1p31.1**	**LHX8**	**75 498 339–75 589 337**	**A**	**G**	**0.43**	**75 498 339–75 577 086**	**All cleft**	**CL/P** **nsCL/P** **CLP**^c^	**1.22**	**3.73E-09**	**No, *P* = 0.94**
rs66515264	1:94548110	1p22.1	ABCA4^a^ (intron)	94 540 675-94 558 110	T	G	0.18	94 548 080–94 558 110	CL/P	All lip phenotypes	1.37	4.38E-10	Yes, *P* = 2.98E-22
rs126280	1:210019824	1q32.2	UTP25^a^ (intron)	210 019 824-210 020 013	A	G	0.23	209 950 760–210 022 893	CLO	nsCL/PCL/P, CLP	1.49	1.86E-18	Yes, *P* = 8.52E-14
rs13385292	2:16726456	2p24.2	CYRIA^a^ (3′ UTR)	16 634 551–16 774 094	T	G	0.3	16 706 891–16 733 054	CL/P	All lip phenotypes	1.40	1.08E-14	Yes, *P* = 3.66E-11
rs4952552	2:42198217	2q14.2	PKDCC/LINCO2898	42 181 679–42 213 484	A	G	0.47	42 179 157–42 215 172	CL/P	All Cleft nsCL/P CLP^d^	0.77	2.66E-09	Yes, *P* = 0.0074
rs6741434	2:43695148	2p21	THADA^a^ (downstream transcript)	43 540 125–43 741 440	T	C	0.24	43 540 125–43 741 440	nsCL/P	CL/P CLO	1.36	2.45E-09	Yes, *P* = 0.0015
rs79482068	3:189541869	3q28	TP63^a^ (intron)	189 537 013-189 553 372	T	C	0.06	189 541 869–189 547 735	CL/P	All lip phenotypes	1.69	2.97E-13	Yes, *P* = 5.58E-05
rs1347188	4:124743259	4q28.1	LINC01091^a^ (intron)	124 743 259-12 474 571	G	A	0.24	124 678 521–124 766 956	CLP	CL/P nsCL/P	1.37	2.58E-08	Yes, *P* = 0.0171
**rs62390705**	**5:127913096**	**5q23.3**	**FBN2**	**127 911 108–127 914 018**	**G**	**A**	**0.13**		**CLO**	**CL/P** **nsCL/P**^d^	**1.58**	**4.00E-08**	**Yes, *P* = 0.0088**
**rs72823645**	**6:8359186**	**6p24.3**	**SLC35B3**	**8 359 186–8 359 186**	**T**	**C**	**0.05**	**8 359 186–8 359 186**	**CPO**	**All cleft** **nsCPO**	**1.69**	**3.67E-08**	**No CPO**
**rs28361060**	**6:32303848**	**6p21.32**	**TSBP1** ^a^ **(intron)**	**32 273 060-32 303 848**	**A**	**G**	**0.22**	**32 212 655–32 338 386**	**All cleft**	**All lip phenotypes** ^c^	**0.75**	**7.97E-09**	**No, *P* = 0.1524**
**rs17168736**	**7:136304959**	**7q33**	**CHRM2**	**136 304 959–136 304 959**	**A**	**C**	**0.18**	**136 296 025–136 321 655**	**nsCPO**	**All clefts** **CPO**	**1.56**	**3.35E-08**	**No CPO**
rs12543318	8:88868340	8q21.3	DCAF4L2	88 868 340–89 017 568	C	A	0.35	88 868 340–89 017 568	CL/P	All lip phenotypes	1.30	5.67E-10	Yes, *P* = 3.09E-11
rs17242358	8:129964873	8q24.21	CCDC26/ LINC00976	128 194 706–130 023 578	A	G	0.18	129 929 311–129 990 382	CL/P	All lip phenotypes	1.92	3.36E-42	Yes, *P* = 1.2E-62
rs7870795	9:100574120	9q22.33	FOXE1/ PTCSC2^a^	100 573 164-100 658 123	T	C	0.29	1 005 753 164–100 620 730	All cleft	All lip phenotypes^c^	0.77	3.78E-11	Yes, *P* = 9.28E-06
rs1898349	10:118817067	10q25.3	SHTN1^a^ (upstream transcript)	118 786 985–118 869 886	T	C	0.17	118 786 985–118 869 886	CL/P	All cleft nsCL/P CL/P^e^	1.37	1.40E-09	Yes, *P* = 8.31E-10
rs1854110	13:80701485	13q31.1	SPRY2	80 679 302–80 702 065	C	T	0.46	80 689 877–80 701 485	CLP	All cleft nsCL/P CL/P^e^	1.47	3.78E-13	Yes, *P* = 7.47E-15
rs10483604	14:51485195	14q22.1	TRIM9^a^ (intron)	51 427 267-51 490 767	T	C	0.23	51 427 267–51 490 767	CL/P	All cleft nsCL/P CL/P^e^	1.33	1.37E-09	Yes, *P* = 0.0143
rs4901118	14:51856109	14q22.1	FRMD6	51 856 109–51 856 566	G	A	0.39	51 856 109–51 856 566	CL/P	nsCL/P CLP CLO	0.77	1.51E-09	Yes, *P* = 1.58E-08
rs2600519	15:33063809	15q13.3	FMN1^a^	33 058 523-33 064 636	G	A	0.34	33 058 304–33 064 636	CL/P	All cleft nsCL/P CLP^e^	0.75	7.40E-10	Yes, *P* = 3.42E-06
rs8044196	16:3984034	16p13.3	CREBBP_ADCY9^a^	3 918 004-3 997 030	A	G	0.33	3 918 004–3 985 081	CL/P	All cleft lip phenotypes	1.33	4.58E-11	Yes, *P* = 0.0007
**rs12971753**	**19:7499238**	**19p13.2**	**ARHGEF18** ^a^ **(intron)**	**7 447 672–7 529 415**	**C**	**T**	**0.23**	**7 447 672–7 529 415**	**CL/P**	**All cleft** **nsCL/P** **CLP**^e^	**0.73**	**3.42E-09**	**No, *P* = 0.082 (same direction)**
**rs2210119**	**20:6506889**	**20p12.3**	**CASC20** ^a^ **(intron)**	**6 428 764–6 591 010**	**C**	**T**	**0.23**	**6 428 764–6 591 010**	**CPO**	**PRS** ^f^	**0.68**	**4.37E-09**	**No, CPO**
rs34753522	20:39278391	20q12	MAFB	39 268 516–39 278 391	C	T	0.36	39 268 516–39 278 391	CL/P	All cleft nsCL/P CLP^e^	0.76	6.84E-10	Yes, *P* = 0.0056
rs6018099	20:45473014	20q13.12	EYA2	45 440 006–45 486 817	G	A	0.48	45 440 006–45 486 817	CL/P	All cleft nsCL/P CLP^e^	1.30	6.32E-10	Yes, *P* = 0.0018

Mapped gene is either the one which the SNP falls within or the nearest gene to the variant according to dbSNP (https://www.ncbi.nlm.nih.gov/SNP).

Table Key: A1 = minor and effect allele, A2 = reference allele,

^a^= falls within the gene region

^b^=taken from GWAS of Europeans in paper lead phenotype = phenotype which has the smallest p-value for association with the lead phenotype,

^c^Nominally associated with all phenotypes p < 0.05,

^d^Effect in opposite direction for PRS, p < 0.05,

^e^Also nominally associated with CLO p < 0.05,

^f^Effect not present in nsCPO, greatest effect size in PRS only cases OR = 1.7

**Table 3:** Evidence linking GWAS significant regions from case–control analyses to orofacial cleft.

rsID	CHR	Chromosome location (GRCh37)	Nearest gene	Prior evidence from GWAS			Gene function	Other evidence linking gene to cleft	References
				**SNP**	**Gene**	**Region**			
rs61769781	1	16 544 626	ARHGEF19	X	X	X	*Guanine nucleotide exchange factor (GEF) for RhoA GTPase. Regulation of cell shape, small GTPase mediated signal transduction*	Gene previously associated with facial morphology (right endocanthion-left cheilion distance).	[31]
rs9439714	1	18 976 489	PAX7	✓	✓	✓	*Paired box (PAX) family of transcription factor critical role during fetal development and cancer growth.*		[24, 28, 29]
rs4112328	1	75 500 597	LHX8	X	X	X	*Transcription factor that plays a role in patterning and differentiation of tissues including tooth morphogenesis.*	Disruption of *LHX8* gene function caused impairment in palatal shelf contact and fusion, leading to formation of a cleft secondary palate. Vieira [32] sequenced DNA from CLP patients and found a mutation for LHX8	[32, 33]
rs66515264	1	94 548 110	ABCA4	✓	✓	✓	*Transport of molecules across extra- and intracellular membranes—retina specific*	Nearest GWAS SNP rs3789432 at 1:94575308	[13, 15, 24, 29]
rs126280	1	210 019 824	UTP25 (IRF6)	X	✓	✓	*Component of ribosome, enables RNA binding activity. Regulates p53 and is essential for embryonic development.*	*Just upstream of IRF6* Nearest GWAS SNP rs2064163, 1:210048819. Previous candidate SNP analysis found associations with same SNP [27].	[13, 15, 24, 27, 34–36]
rs13385292	2	16 726 456	CYRIA	X	✓	✓	*Predicted to enable small GTPase binding activity and involved in regulation of actin filament polymerization.*	Nearest GWAS lead SNP rs4832655 2:16725432	[15, 19, 20]
rs4952552	2	42 198 217	PKDCC LINC02898	X	✓	✓	*Secreted tyrosine-protein kinase that mediates phosphorylation of extracellular proteins and endogenous proteins in the secretory pathway.*	Previously associated with cleft parent endophenotype [38]. Imuta et al. [37] generated Pkdcc −/− mice, resulted in shortening of the long bones of the limbs and cleft palate.	[24, 37–39]
rs6741434	2	43 695 148	THADA	X	✓	✓	*Methylates the 2'-O-ribose of nucleotides at position 32 of the anticodon loop of substrate tRNAs.*		[12, 14, 29]
rs79482068	3	189 541 869	TP63	X	✓	✓	*Transcription factor, Involved in NOTCH signalling by probably inducing JAG1 and JAG2. Plays a role in the regulation of epithelial morphogenesis.*	Nearest GWAS SNP rs76479869, 3:189553372 (also sign in our analysis).	[24]
rs1347188	4	124 743 259	LINC01091	X	✓	✓	*Long Intergenic Non-Protein Coding RNA 1091.*		[15, 17]
rs62390705	5	127 913 096	FBN2	X	X	X	*Component of connective tissue microfibrils and may be involved in elastic fibre assembly.*	SNPs at position 128 107 907 and 128 190 363 have been found to be associated with lip morphology. A syndromic form of PRS is caused by a deletion in 5q23 including FBN2	[40–42]
rs72823645	6	8 359 186	SLC35B3	X	X	X	*Solute Carrier Family 35 (Adenosine 3'-Phospho 5'-Phosphosulfate Transporter) gene.*	Related pathway involved in chondrodysplasia with Joint Dislocations, Gpapp Type (CDP-GPAPP), disorder characterised by short stature, chondrodysplasia with brachydactyly, congenital joint dislocations, cleft palate, and facial dysmorphism.	[43]
rs28361060	6	32 303 848	TSBP1	X	X	X	*Testis expressed basic protein. Associated with connective tissue diseases.*	Just upstream of *NOTCH4* involved in craniofacial development.	
rs17168736	7	136 304 959	CHRM2	X	X	X	*Muscarinic cholinergic receptors belong to a larger family of G protein-coupled receptors*	Associated with psychiatric disease, alcohol use disorder and cognitive function (GWAS catalog). Children born with 7q33 deletions had dysmorphic facial features including a narrow palate.	[44]
rs12543318	8	88 868 340	DCAF4L2	✓	✓	✓	*DCAF4L2 = Protein that regulates epithelial-mesenchymal transition (EMT) by activating NFKB signaling.*		[15, 20, 24, 29, 39, 45, 46]
rs17242358	8	129 964 873	CCDC26 LINC00976	✓	✓	✓	*Long Non-Protein Coding RNA*		[13–15, 24, 29, 46–49]
rs7870795	9	100 574 120	FOXE1 PCTSC2	X	✓	✓	*A thyroid transcription factor that plays a role in thyroid morphogenesis*	Involved in cleft palate formation. Nearest GWAS SNP rs12347191 in 9:100619719 (also sign in our analysis)	[24, 28, 45, 50]
rs1898349	10	118 817 067	SHTN1	✓	✓	✓	*Involved in positive regulation of neuron migration.*		[12, 14, 15, 24, 36, 46, 48, 51]
Rs1854110	13	80 701 485	SPRY2	✓	✓	✓	*Inhibitory activity on receptor tyrosine kinase signaling proteins. Bimodal regulator of epidermal growth factor receptor/mitogen-activated protein kinase signaling.*	Sole locus to have previously been identified with an effect on CLP but not CLO.	[12, 14, 15, 19, 29, 39, 46, 47]
rs10483604	14	51 485 195	TRIM9	X	X	✓	*Member of the tripartite motif (TRIM) family, function not identified.*	Within 40 kb of hit below	[14, 39]
rs4901118	14	51 856 109	FRMD6	✓	✓	✓	*FRMD6 = may be involved in maintaining cytoskeleton.*		[39]
rs2600519	15	33 063 809	FMN1	X	✓	✓	*Protein has a role in the formation of adherens junction and the polymerization of linear actin cable**.***	Associated with Robinow Syndrome, Autosomal Recessive 2 (RRS2) characteristics include facial dysmorphology (genecards)	[12, 14, 39, 46, 47]
rs8044196	16	3 984 034	CREBBP ADCY9	X	X	✓	*Transcription factor regulator, plays a critical role in embryonic development.*	Mutation in CREBBP recently identified in case with CPO [21].	[21, 39, 51]
rs12971753	19	7 499 238	ARHGEF18	*X*	*X*	*X*	*Guanine nucleotide exchange factor (GEF) for RhoA GTPase. Regulation of actin cytoskeleton.*	Mutation in ARHGEF18 identified in patient with cleft lip and palate	[52]
rs2210119	20	6 506 889	CASC20	*X*	*X*	X	*Cancer susceptibility gene 20*	Region associated with facial morphology	[41]
rs34753522	20	39 278 391	MAFB	X	✓	✓	*MAF bZIP transcription factor B*	*MAFB* is a transcription factor	[24, 29, 36, 51]
rs6018099	20	45 473 014	EYA2	X	X	✓	*Eyes absent 2 gene. Transcription factor involved in early embryonic development*	Expressed in early embryonic development. Region associated with cleft endophenotype	[19, 38]

Lead SNPs rs4112328 (*LHX8,* 1p31.1*)*, rs28361060 (*TSBP1,* 6p21.32) and rs7870795 *(FOXE1,* 9q22.33) had the smallest p-values in the "all cases" GWAS and were nominally significant (*P* < 0.05) in the same direction across all subgroups. Most of the other SNPs we identified were genome-wide significantly associated with cleft lip phenotypes or cleft palate only phenotypes but were not strongly associated with both and so the smallest p-values for these SNPs were not in ‘all cases’. After clumping, we identified 18 regions (2 mapping to *ARHGEF18* (19p13.2) and *ARHGEF19* (1p36.13) were novel) which showed the smallest p-values in the cleft lip with or without cleft palate (CL/P) or nsCL/P subtype GWAS and which were associated with both CLO and CLP. Additionally, rs62390705 (a novel region mapping to *FBN2,* 5q23.3) was associated with CLO but not CLP (Cochrane’s Q p-value for heterogeneity = 0.002), rs1347188 (*LINC01091,* 4q28.1) was associated with CLP but not CLO (Cochrane’s Q p-value for heterogeneity =0.020) and finally rs1854110 (*SPRY2,* 13q31.1) showed strong evidence of association with CLP (OR = 1.46, *P* = 1.01 × 10^−12^) but much weaker evidence of an effect on CLO (OR = 1.14, *P* = 0.04) (Cochrane’s Q p-value for heterogeneity = 0.0007). All SNPs which were genome-wide significant for CL/P were also strongly associated (at least *P* < 1 × 10^−6^) with nsCL/P, and vice versa.

Finally, we identified three novel loci primarily associated with cleft palate only (CPO). Lead SNP rs72823645 (*SLC35B3,* 6p24.3*)* was associated with both non-syndromic cleft palate only (nsCPO) and Pierre Robin sequence (PRS) (albeit not genome wide significant in PRS, *P* = 2.82 × 10^−5^), as well as being nominally associated with cleft lip phenotypes with attenuated effects in the same direction. Lead SNP rs17168736 (*CHRM2,* 7q33*)*, was strongly associated with nsCPO and weakly with cleft lip phenotypes in the same direction but was not associated with PRS. The third lead SNP, rs2225351 (*CASC20,* 20p12.3*)* was genome wide significant for CPO and was approaching genome wide significant for PRS (other SNPs in this region were genome wide significant for PRS), with a weaker effect on nsCPO, and no association with cleft lip phenotypes. Table 3 shows a summary of the evidence linking each of the GWAS significant regions to orofacial cleft.

### Pierre Robin sequence (PRS)

Although we identified only one genome-wide significant region for PRS (*CASC20,* 20p12.3), we found 21 additional genomic regions with suggestive associations in our case–control analysis (*P* < 1 × 10^−5^) (Table 4*).* The identified regions have not previously been linked to orofacial clefts in GWAS. Several of these regions or mapped genes however have been associated with facial morphology in GWAS [38, 40, 73], linked to syndromes characterised by facial features [59], or have shown evidence of involvement in orofacial clefts in animal studies [55, 65].

**Table 4:** Results of clumped SNPs showing suggestive associations (p < 1x10^−5^) with Pierre Robin sequence in case–control analysis**.**

RSid	CHR	Chromosome Band	Location (GRCh37)	Nearest Gene	Region *P* < 1E-04	A1	A2	OR	P-value	Gene function	Evidence from other studies linking gene or region to cleft
rs7532822	1	1p36.13	20 171 884	RNF186	20 161 864 to 20 249 510	T	C	1.64	2.75E-06	E3 ubiquitin ligase that regulates endoplasmic reticulum (ER) stress and apoptosis [53]	Unbalanced translocation found in 1p36 in a patient with PRS [54].
rs1481347	1	1q41	218 766 058	TGFB2	218 744 152 to 218 813 229	A	G	1.61	3.97E-07	Transforming growth factor	Tgfb2-knock-out mice had a wide range of developmental defects including craniofacial defects such as retrognathia, dysmorphic calvaria, and cleft palate [55]
rs73186867	3	3q27.3	187 646 163	LINCO1991	187 649 163	T	C	1.96	6.93E-06	Non-coding RNA (ncRNA) and has been linked to gene regulatory mechanisms	
rs1964334	5	5q15	94 889 277	SKIC3 (TTC37)^a^/ ARSK^a^ (upstream_ transcript_variant, 5’ UTR Variant)	94 799 142 to 94 938 392	T	G	1.74	8.30E-06	SKIC3 is part of a multiprotein complex that assists the RNA-degrading exosome during the mRNA decay and quality-control pathways [56]. ARSK Hydrolyzes sulfate esters from sulfated steroids, carbohydrates, proteoglycans, and glycolipids. Involved in hormone biosynthesis, modulation of cell signaling, and degradation of macromolecules [57].	*SKIC3* gene is highly expressed in placenta, mutations in this gene cause trichohepatoenteric syndrome one of the features of which is facial dysmorphism (prominent forehead and cheeks, broad nasal root and wide-spaced eyes) [56]. A study of 4 patients with functional mutations in *ARSK* found that all 4 had mild coarse facial features, midface retrusion, full lips as well as disproportionate short-trunk, short stature, and genu valgus [58]
rs4960288	6	6p25.1	7 090 193	RREB1	7 090 193 to 7 091 385	C	T	1.71	5.41E-06	Zinc finger transcription factor that binds to RAS-responsive elements (RREs) of gene promoters. RREB1 haploinsufficiency leads to sensitization of MAP Kinase signalling.	Seven patients with a deletion in this gene had clinical features consistent with a Noonan-spectrum disorder, including short stature, dysmorphic facial features (wide-set eyes, broad forehead, wide nasal base, and downward slanting palpebral fissures), and cardiovascular abnormalities [59].
rs693602	6	6q22.1	117 085 784	FAM162B^a^ Upstream transcript variant	117 082 582 to 117 157 774	A	C	1.52	8.61E-06	Predicted to be integral component of membrane	Diseases associated with FAM162B include Waardenburg syndrome characterized by varying degrees of deafness and minor defects in structures arising from neural crest, including pigmentation anomalies of eyes, hair, and skin [60]. Facial features include wide nasal bridge, wide spacing of inner corners of the eyes.
rs1752332	9	9q31.3	112 859 074	PALM2AKAP2^a^ Transcript variant	112 855 644 to 112 860 621	G	C	1.62	5.09E-06	Binds to and modulates the structure of the actin cytoskeleton	Associated with developmental and epileptic encephalopathy 37 and scoliosis (prominent forehead, down slanting palpebral fissures, drooping eyelids) [61]. Gene associated with nose morphology [40].
rs28589413	9	9q34.3	140 329 618	ENTPD8^a^ (intron)/NOXA1	140 298 718 to 140 358 581	A	G	1.61	1.57E-06	ENTPD8 is a liver-enriched, plasma membrane-bound enzyme that regulates extracellular nucleotide concentrations	
rs11255521	10	10p14	8 139 959	GATA3	8 139 237 to 8 144 673	C	A	0.62	2.28E-06	Transcription factor, interacts with BMP signaling, essential for neural crest cell migration into pharyngeal arches during craniofacial development.	Role in the differentiation of multiple cell lineages during embryogenesis [62]. Loss of GATA3 function has been associated with palatal defects, particularly affecting the trabeculae, a structure critical to proper palate formation​ [63]. Mutations in this gene cause Hypoparathyroidism, Deafness and Renal dysplasia (HDR) syndrome [64].
rs7896113	10	10q21.3	65 581 452	REEP3	65 541 174 to 65 591 322	C	T	1.74	3.92E-06	Transport of G protein-coupled receptors (GPCRs) to the cell surface membrane, critical for receptor-ligand recognition	Diseases linked to gene include Adult Hypophosphatasia and Autism (www.genecards.com).
rs67831458	11	11p11.2	44 927 625	TSPAN18^a^ (intron)	44 924 728 to 44 927 625	C	T	1.88	1.54E-06	Gene involved in cellular signalling and adhesion, particularly in endothelial and immune cells.	Gene expressed in chick cranial premigratory neural crest cells [65]. Associated with Brain measurement in GWAS [66]
rs1871396	12	12p12.1	21 335 781	SLCO1B1^a^ (intron)	21 253 270 to 21 408 632	T	C	1.56	1.82E-06	Encodes a liver transporter protein involved in drug metabolism and bile acid transport.	
rs6580928	12	12q13.13	53 474 963	SPRYD3^a^ (upstream transcript variant)	53 471 929 to 53 522 814	C	G	1.69	2.24E-06	Regulatory gene, predicted to be involved in cell surface receptor signalling pathway and cytoskeleton organization	
rs7983347	13	13q14	43 271 058	TNFSF11	43 271 058 to 43 284 032	A	G	1.55	4.83E-06	Member of the tumor necrosis factor (TNF) family, ligand for osteoprotegerin and functions as a key factor for osteoclast differentiation and activation.	Mutations in this gene cause osteoclast-poor osteopetrosis [67]
rs9527944	13	13q21.1	59 709 993	DIAPH3	59 709 993 to 59 711 110	C	T	1.59	2.73E-06	Involved in actin remodeling and regulating cell movement and adhesion.	Mutations in this gene are associated with autosomal dominant auditory neuropathy 1 [68]
rs12591226	15	15q21.3	54 677 559	UNC13C^a^ (intron)	54 651 171 to 54 693 478	C	T	1.63	7.67E-06	Plays a role in the regulation of synaptic vesicle priming and neurotransmitter release, important component of neural communication and brain function.	A SNP in this gene has previously been associated with orofacial clefts [69], dental anomalies [70] and facial morphology measurement—The distance between the crista philtri and stomion [71]
rs4777036	15	15q23	68 605 568	ITGA11^a^ (intron)	68 553 914 to 68 605 568	T	C	1.57	3.85E-06	Encodes the alpha-11 subunit of integrin, a protein that plays a critical role in cell adhesion, migration, and signaling.	Nearby SNP at locus 68 763 788 is associated with morphology of the chin and outer jaw [40]. Region associated with cleft endophenotype [38]
rs34555955	17	17q22	53 916 012	PCTP^a^ (downstream transcript variant)	53 830 710 to 53 916 012	G	A	1.64	8.92E-06	Catalyses the transfer of phosphatidylcholine between membranes.	Gene associated with brain volume measurement in GWAS [72]. Region associated with cleft endophenotype [38]). Nearby SNP in 17q22 locus identified as having an opposing effect on CPO versus CL/P [28]
rs6045431	20	20p11.23	18 465 583	POLR3F^a^ (downstream transcript variant)	18 452 664 to 18 568 962	T	G	1.74	3.97E-06	Gene encoding a protein that forms part of RNA polymerase III (Pol III). This enzyme is crucial for the transcription of small non-coding RNAs, including 5S ribosomal RNA, tRNA, and certain microRNAs.	
rs6016331	20	20q12	38 988 839	TOP1	38 918 153 to 39 038 828	G	A	1.78	2.90E-06	Catalyses the breaking and rejoining of DNA strands in a way that allows the strands to pass through one another, thus altering the topology of DNA	
rs1988821	21	21q21.2	26 292 166	LINCO1692^a^ (intron)	26 216 316 to 26 324 293	A	G	1.57	1.58E-06	codes for a type of long non-coding RNA (lncRNA), which plays roles in regulating gene expression.	

^a^= falls within the gene region.

Supplementary Figs 2–44 show Manhattan, Quantile-Quantile (Q-Q) and Locus Zoom plots of our analyses.

### Replication

Almost all of our lead SNPs with the strongest evidence of association in CL/P or nsCL/P were replicated (with at least nominal levels of significance *P* < 0.05 and effects in the same direction) in the meta-analysis by Welzenbach et al. [19] (see Table 2), with some also being GWAS-significant in that study. Two novel SNPs which were genome wide significant in nsCL/P (rs61769781, *ARHGEF19*, 1p36.13) and CLO (rs62390705, *FBN2*, 5q23.3) showed some evidence of association with nsCL/P in the replication meta-analysis. Although rs12971753 (*ARHGEF18,* 19p32.2) did not replicate, this SNP showed weak evidence (*P* = 0.082) in the same direction. Of the three SNPs with the strongest evidence in the "all clefts" group, two were not associated with nsCL/P in the replication sample; however, rs7870795 (*FOXE1,* 9q22.33) showed strong evidence of association. As expected, our CPO hits were not associated with nsCL/P in the replication sample. We wanted to replicate our CPO hits in CPO GWAS, however no GWAS of all CPO (syndromic and non-syndromic cases) or of PRS were available for this purpose and our nsCPO hit was not available in a recent meta-analysis of nsCPO [74].

### Correlation across subgroups

**Figure 2:**
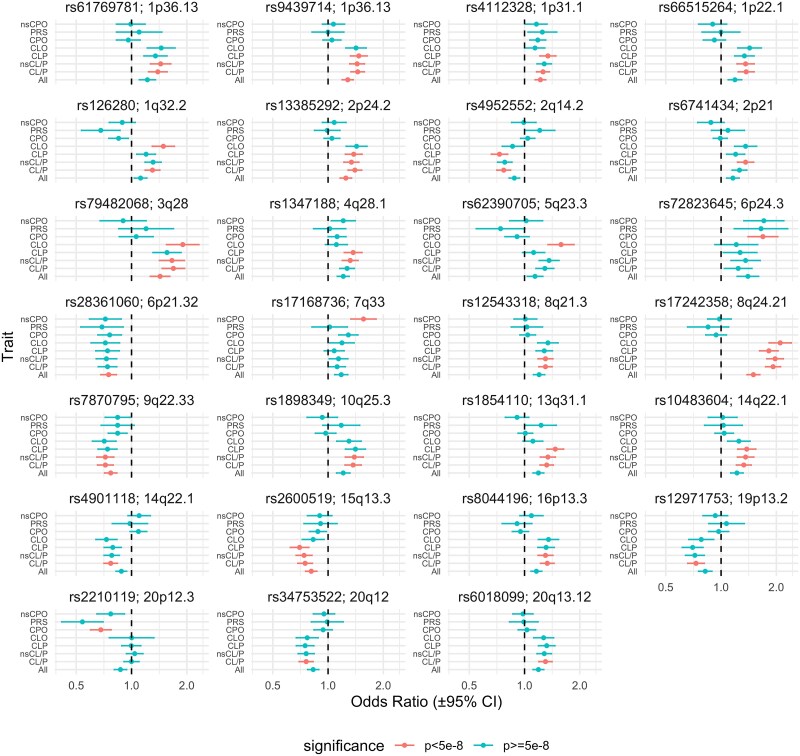
Forest plot of our showing SNP effects across all subgroups for the 27 SNPs reaching genome wide significance in at least one of our GWAS.

Figure 2 shows a forest plot of effect sizes and confidence intervals for each of our 27 genome wide significant hits across all subgroups. For the most part, SNPs primarily associated with cleft lip phenotypes were not associated with cleft palate only phenotypes. However, three SNPs showed some weak evidence of an effect on PRS in the opposite direction to their effect on cleft lip phenotypes: rs62390705 (*FBN2*, 5q23.3) (*P* = 0.05), rs126280 (*UTP25*, 1q32.2) (*P* = 0.002), and rs4952552 (*PKDCC*, 2q14.2) (*P* = 0.04). Along with the three SNPs which showed the strongest evidence of effect across all clefts, there were four other SNPs (rs1347188 (*LINCO1091*, 4q28.1), rs17168736 (*CHRM2*, 7q33), rs2600519 (*FMN1,* 15q13.3), rs72823645 *SLC35B3* 6p24.3) which showed some evidence of association in the same direction across all phenotypes.

**Figure 3:**
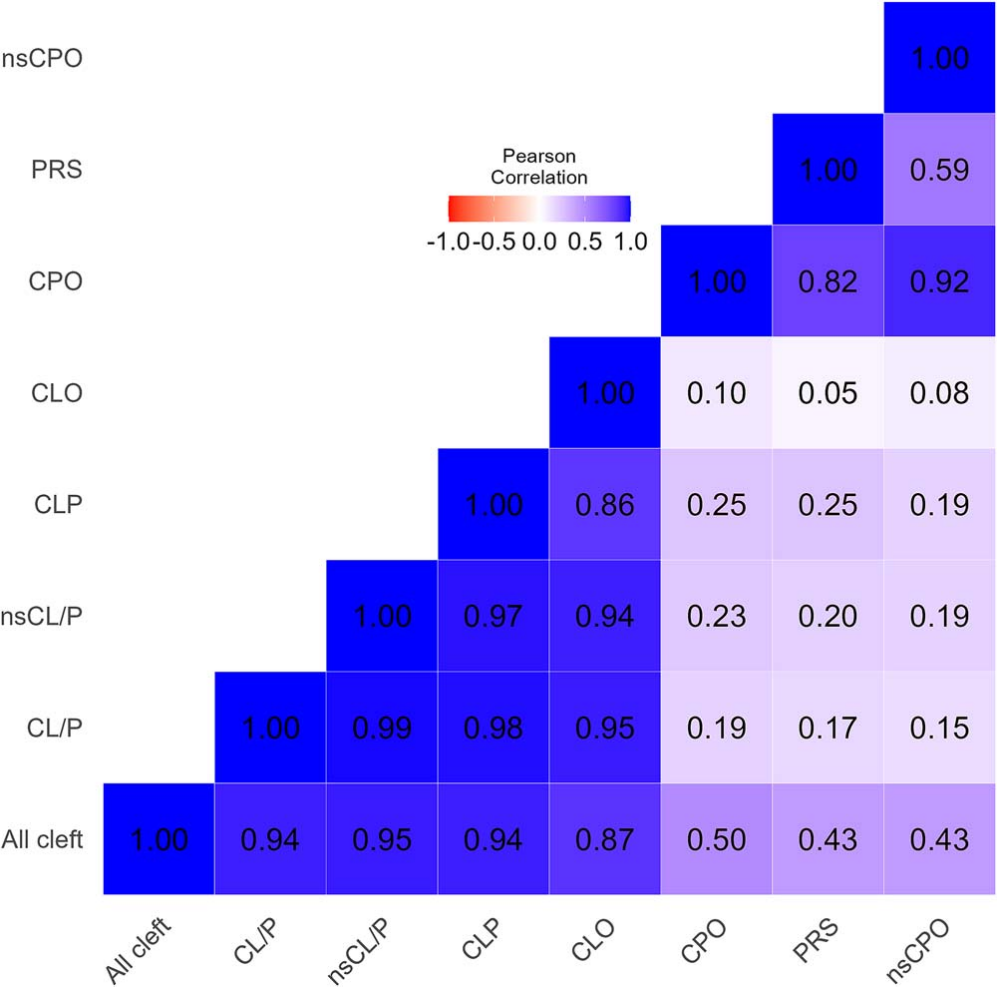
Heatmap showing correlation coefficients based on effect sizes of our 27 lead genome wide significant SNPs across subtypes.

Our correlation matrix of effect estimates for lead SNPs across subgroups (Fig. 3) revealed very strong correlations (> 0.86) between the various cleft lip phenotypes. Despite CLO and CLP being mutually exclusive, they still showed a high correlation. In contrast, correlations between cleft palate only and cleft lip subgroups were much weaker, (Pearson's correlation coefficients ranged from 0.05 to 0.25). Within the CPO subgroups, the correlation between nsCPO and PRS was higher (0.59) than the correlation between CPO and cleft lip subtypes, but indicated that these phenotypes are likely to have different underlying genetic causes. For CL/P, there was almost perfect correlation between all CL/P cases and a subgroup which excluded syndromic cases- nsCL/P (correlation coefficient = 0.99). There was also a strong correlation between all CPO cases and the subgroup excluding syndromic cases (correlation coefficient = 0.92).

### Proportion of variance explained

Our estimates for SNP heritability (calculated using LD-score regression) based on our case–control analysis ranged from 5% (95%CI: −0.07 to 0.17) for PRS to 38% (95%CI: 0.22 to 0.54) for nsCL/P, with the SNP heritability of nsCPO estimated to be around 17% (95%CI: 0.05 to 0.29) (Table 5).

**Table 5:** Whole genome SNP heritability estimated using LD score regression.

	Case–control GWAS			
Phenotype	H^2^SNP	SE	95% CI	nSNP in LDSC
All cleft	0.22	0.06	0.10–0.34	1 041 927
Cleft lip +/− palate	0.37	0.08	0.21–0.53	1 042 073
Non syndromic cleft lip +/− palate	0.38	0.08	0.22–0.54	1 041 995
Cleft lip and palate	0.21	0.07	0.07–0.35	1 041 945
Cleft lip only	0.14	0.06	0.02–0.26	1 042 056
Cleft palate only	0.20	0.06	0.08–0.32	1 042 094
PRS	0.05	0.06	−0.07-0.17	1 042 117
Non syndromic cleft palate only	0.17	0.06	0.05–0.29	1 042 176

### Enriched biological processes

The biological processes which were most enriched in the gene set which mapped to all 27 genome wide significant hits from our case–control analyses were limp morphogenesis (5 genes 25.07 fold enrichment, *P* = 1.46 × 10^−6^, false discovery rate (FDR) adjusted *P* = 0.007) and embryo development (10 genes, 7.29 fold enrichment, *P* = 4.17 × 10^−7^, FDR adjusted *P* = 0.006). Within the gene set identified by the PRS GWAS none of the biological processes showed strong evidence of enrichment.

## Discussion

Our study represents a comprehensive set of GWAS of orofacial clefting, in which we performed GWAS of all clefts combined plus GWAS of 7 orofacial cleft subgroups. We identified several novel loci, replicated key associations from previous studies, and provided new insights into the genetic architecture of orofacial clefts.

### Key findings, novel associations and consistency with other evidence

We identified 27 genome-wide-significant loci, 8 of which were novel. Novel associations were identified between SNPs mapping to *ARHGEF18* and SNPs just upstream of *ARHGEF19* and CL/P. These genes are involved in Rho GTPase signalling pathways, which regulate neural crest cell migration and cytoskeletal organization—processes critical for craniofacial structure development and fusion [75, 76]. We have previously shown *ARHGEF19* to be associated with facial morphology (right endocanthion to left cheilion distance) [31]. In addition, a Mutation in *ARHGEF18* has been previously identified in a patient with cleft lip and palate [52].

*CASC20* has been previously linked to cleft lip, but we did not find an association between SNPs at this locus and cleft lip; to our knowledge ours was the first study to associate it with cleft palate, and specifically to PRS. *CASC* genes are classified as cancer susceptibility genes and may therefore influence cell growth and proliferation, although their functional roles are currently unclear.

Some prior GWAS have been conducted on nsCL/P combined with nsCPO [16], or have investigated differences between cleft subtypes [24–26, 28]. Our study along with those above suggests that GWAS of all cases combined may also be fruitful and future studies, which include all orofacial clefts, with larger sample sizes than ours may find more variants. Three genomic regions mapping to *LHX8, FOXE1*, and *TSBP1* showed the strongest evidence of association when all clefts were combined. All three regions are transcription factors and may therefore have wide ranging effects on development. *LHX8* and *FOXE1* are transcription factors essential for craniofacial development and have been linked to cleft palate [33, 77]. Whilst SNPs in the *FOXE1* region have been associated with different subgroups of orofacial clefts in GWAS [78] and SNPs in this region were genome wide significantly associated with all orofacial clefts in the GWAS by Leslie et al. [24], *LHX8* has not been previously been identified in orofacial cleft GWAS. However, a sequencing study did detect a mutation in *LHX8* in a CLP patient [32]. *TSBP1* is less well-studied but is thought to regulate transcription, and its promoter region includes binding sites for forkhead (FOX) proteins [79]. Additionally, *TSBP1* is located upstream of *NOTCH4*, a gene implicated in craniofacial development [80]. Two further previously identified regions and two novel regions were also nominally associated with all cleft types in the same direction (*LINCO1091, FMN1, SLC35B3, CHRM2*), albeit for these SNPs they exhibited genome wide significant associations with either lip or palate only phenotypes. *SLC25B3*, is a novel locus associated with CPO in our GWAS (and to a lesser extent with nsCL/P), it is a solute transporter gene linked to disorders featuring cleft palate and facial dysmorphism [43]. Another novel locus which we found to be associated with nsCPO mapped to CHRM2 on 7q33, children born with 7q33 deletions have been described as having dysmorphic facial features including a narrow palate [44].

SNPs upstream of the *FBN2* gene were associated with CLO in our analysis. *FBN2* encodes a component of connective tissue microfibrils and has been associated with lip morphology [40, 41]. In addition, a syndromic form of PRS was found to be caused by a deletion in 5q23 including FBN2 [42].

### PRS findings

To our knowledge this was the first GWAS of PRS to date. Our sample size for this subgroup was small, just 237 cases and we were therefore underpowered to detect many potential associations. Nonetheless we did identify one GWAS significant SNP mapping to *CASC20*. We also found a further 21 regions where associations approached GWAS significance. Only two of these regions (*UNC13C* (15q21.3) and *PCTP (*17q22)) had previously been identified in a cleft GWAS, with the region in 17q22 having been identified as having opposing effects on CL/P and CPO in the study by Ray et al., [28]. However, one SNP mapping to the *ITGA11* gene was close to a GWAS hit associated with the morphology of the chin and outer mandible [40]; the same region was also associated with a cleft endophenotype identified in parents of children born with a cleft [38]. Another, region identified in our study was in *SKIC3/ARSK* (5q15), a previous study found that four children with functional mutations in this gene had an under-developed upper jaw [58]. In addition, mutations in *SKIC3* cause trichohepatoenteric syndrome, one of the features of which is facial dysmorphism (prominent forehead and cheeks, broad nasal root and wide-spaced eyes) [64]. *GATA3* and *TGFB2* have been shown to be involved in palate formation with knock-out mice displaying palatal defects [55, 81]. A further three regions mapped to genes associated with syndromes which have craniofacial anomalies as a feature (*RREB1, FAM162B, PALM2AKAP2*) [59–61, 82], common facial features of these syndromes are broad prominent foreheads, wide nasal bridge and widely spaced eyes. The *PALM2AKAP2* locus had also been found to be associated with nasal morphology [40]. These genes warrant future research, although replication in larger GWAS is required.

### Phenotypic and genetic correlations

The correlation matrix of effect estimates across subgroups (Fig. 3) revealed strong genetic overlap within cleft lip phenotypes, consistent with shared genetic underpinnings. The weaker correlations between cleft lip and cleft palate phenotypes support their classification as distinct entities. We found two regions to be genome wide significant in our CLP subgroup, which were not strongly associated with CLO (with evidence of heterogeneity between them), one of which maps to *SPRY2*. The CLP-specific effect of this locus has been shown in previous analyses [14, 83], although similar effects were seen for both subtypes in another study [29]. The other CLP specific locus we identified was at 4q28.1. Similar to our study, Yu et al. [15] found strong evidence for this SNP being associated with CLP but weaker evidence for association with CLO. Both Ray et al. [28] and Moreno Uribe et al. [29] found evidence of differential effects of SNPs in the ABC4A locus 1p22.1 on CLO and CLP, however, we found similar effects at this locus for both subtypes.

Like Ray et al. [28] and Alade et al. [25] we found SNPs which had opposing effects on CL/P compared with CPO. We found the greatest difference in effect estimates to be between PRS compared to cleft lip only, suggesting that the two subtypes might be two opposite extremes resulting from disruptions to shared developmental processes. The three genes implicated in having opposing effects on PRS and cleft lip -*IRF6/UTP25, PKDCC, and FBN2* are all associated with limb and connective tissue anomalies. *FBN2* mutations cause congenital contractural arachnodactyly, a rare autosomal dominant connective tissue disorder characterized by joint contractures, arachnodactyly, scoliosis, and crumpled ears [84]. *PKDCC* mutations result in rhizomelic limb shortening with dysmorphic features, which includes short upper limbs, macrocephaly, a prominent forehead, hypertelorism, a broad nasal bridge, and mandibular hypoplasia [85, 86]. *IRF6* mutations, in addition to causing Van der Woude syndrome, can cause popliteal pterygium syndrome, an autosomal dominant disorder characterized by orofacial anomalies including lower lip pits, cleft lip and/or palate, syngnathia, syndactyly, webbing of the lower limbs, and genital abnormalities [87]. The opposing effects of SNPs near these genes on CLO and PRS may therefore be related to their roles in regulating connective tissue development.

Support for our findings comes from a previous study examining candidate SNPs in the *IRF6* region which found that rs126280 was the driver of associations between *IRF6* haplotypes and orofacial clefts. Notably, that study also reported that rs126280 showed opposing effects on CPO and CLP—consistent with the direction of effects we observed [27]. In addition, Ray et al., [28] found that a SNP between *IRF6* and *UTP25,* which is around 30 kb from our SNP, was associated with an increased risk of CPO but a reduced risk of CLP. *UTP25* is a regulator of p53 and is essential for embryonic development [34, 35], so this may have effects on cleft which are independent of *IRF6*. Alternatively *UTP25* may contain regulatory regions for the *IRF6* gene.

Consistent with previous studies, our study identified SNPs at the *FOXE1* locus 9q22.33 that exhibit similar effects across CL/P and CPO subtypes [26, 28, 29]. In contrast, our findings diverged from those of Ray et al. [28] at the *MAFB* (20q13) loci as they found similar effects across CL/P and CPO subtypes. We observed that this region was associated with CL/P and CLO but not with CPO, aligning with the results of Moreno Uribe et al. [29], also in line with that study, we found that several other loci—*PAX7, IRF6, THADA* and, 8q21.3, 8q14, and *MAFB*—were associated with CLO and CL/P but not with CPO. Regarding the *PAX7* locus on 1p36.13, our study observed a strong association with CL/P but not with CPO. This again contrasts with the findings of Ray et al. [28], who reported effects in the opposite direction. The absence of effects in the opposite direction in our study suggests that *PAX7* may play a more prominent role in CL/P than in CPO.

Incorporating syndromic cases into our CL/P GWAS analysis had minimal impact on SNP effect estimates, as indicated by a high correlation (r = 0.99) between the full dataset and the subset excluding syndromic cases, whereas including syndromic cases increased the sample size by 26%. These results suggest that (a) restricting the analysis to non-syndromic cases is unnecessary and (b) common genetic variants may contribute similarly to both syndromic and non-syndromic cases. Our findings align with Wilson et al. [21], who reported that the distinction between these groups is not well-defined and Moreno Uribe et al., [29] who have previously shown SNP effects in the same direction for syndromic CL/P and nsCL/P. Given that information on syndrome diagnosis is sometimes not available, and syndromes are often diagnosed later in life or underdiagnosed [3], it is reassuring to know this is unlikely to influence results.

We estimated using SNP heritability that 38% of the variation in nsCL/P and 17% of the variation in nsCPO was explained by common genetic variation. The estimate for nsCL/P is slightly higher than a previous estimate of 32% (standard error = 8.5%) reported in 2017 [39], though the confidence intervals overlap. It is also comparable to an estimate of 37% generated in a Chinese cohort [88]. Lower heritability estimates derived from genome-wide association study (GWAS) significant SNPs have been reported at 11% based on 26 SNPs in a multiethnic GWAS [15] and 25.5% based on 24 SNPs in a cohort from Bonn [39]. To our knowledge, this is the first study to estimate SNP heritability for nsCPO. Heritability estimates are specific to the population in which they are measured, therefore given that our study sample lacked ethnic diversity and was based on a European ancestry population, these estimates might not to be applicable to other populations.

### Strengths and limitations of the study

Our study has several notable strengths. First, we utilized a well-characterized case population with detailed classification of cleft subtypes. Combined with a large, population-based control group at an approximate 4:1 control-to-case ratio, this significantly enhanced the statistical power of our analysis.

Another strength of our study was the ability to compare genetic effect estimates across multiple cleft subtypes. This analysis revealed risk loci shared across all cleft subtypes as well as those specific to cleft lip only, cleft palate only, or cleft lip and palate. Furthermore, the cross-subtype comparison identified genes exhibiting opposing effects on PRS and cleft lip. This intriguing finding warrants further exploration to identify the mechanism involved.

Our study had several limitations, the most significant being the small number of cases in many subgroup analyses, particularly for PRS. To address this, we focused not only on p-values but also on effect sizes across subgroups for SNPs that reached genome wide significance in at least one analysis, and we included a replication sample. Despite these efforts, we likely missed many regions associated with cleft.

We investigated the correlation between subtypes with and without syndromes, however we expected these to be correlated to a certain extent as one is a subset of the other. Ideally we would have investigated the correlation of non-syndromic and syndromic forms of CL/P, but we were not able to do this because the sample size for syndromic cases with a cleft lip was small. In addition, we were unable to use whole genome methods to estimate genetic correlation and some of the correlation coefficients between subgroups are likely to have been biased due to random fluctuations in effect estimates, both due to small sample size. In the future, we plan to enhance our analyses by integrating data from other cleft studies through meta-analyses to increase sample sizes.

Many of the SNPs we identified were located in non-coding regions of the genome and are unlikely to be the causal variants. We mapped SNPs to the nearest gene, but this is not necessarily the gene involved in orofacial clefts. For example, SNPs, such as the ones we identified, in the *ABCA4* gene have previously been shown to be in enhancer regions which control *ARHGAP29* expression and evidence suggests that it is *ARHGAP29* not *ABCA4* that plays a role in orofacial clefting [89]. Future studies are required to pinpoint causal variants in these regions and to clarify the biological processes through which the associated genes influence cleft formation.

## Conclusion

The identification of novel loci and the replication of known regions highlights the robustness of our approach. The novel associations, particularly for CPO and PRS expand the genetic landscape of orofacial clefts and may inform future studies of craniofacial development. Functional validation of the implicated genes and loci, particularly those with pleiotropic effects, is a critical next step. Larger studies with more diverse populations which follow our approach are likely to capture even more genetic variation contributing to orofacial clefts. The interplay of genetic and environmental factors, particularly for rarer phenotypes such as PRS remains an important area for future research.

## Materials and methods

### Ethical approval

All study participants were recruited with informed parental consent. Ethical approval was granted for the Cleft Collective by the NRES committee South-West (13/SW/0064). The Millennium cohort study obtained ethical approval from London-Central Research Ethics Committee (13/LO/1786).

### Study population and sample

#### Orofacial cleft families

Children born with an orofacial cleft and their families who were registered at one of 17 cleft centres across the UK were recruited to the Cleft Collective Cohort study between November 2013 and June 2020. Parents who consented to participate were sent a questionnaire to complete and were asked to provide a saliva sample, both of which were returned by post, the questionnaires to the Cleft Collective team and the biological samples to the Bristol Bioscience Laboratories. For children recruited before their first surgery, blood and tissue samples were collected during the procedure. For those cases recruited at age 5, saliva samples were obtained at the five-year audit clinic.

#### Controls

Controls were from the Millenium Cohort Study, a population-based study of children who were born across the UK over a 17 month period (September 2000–January 2002). The study is coordinated from the University College London [90], although processing of the biological samples was performed at the University of Bristol. Saliva samples were collected from 9259 children in the cohort during a home visit by the study team in 2015–2016 when they were approximately 14 years old [91].

### Case definition

Information on cleft subtypes including syndrome status came from either the parent questionnaire, forms completed by surgeons at the time of cleft surgery or from clinical notes. We initially grouped all cases together to form a group labelled ‘all clefts’. In addition, we performed orofacial cleft subtype analyses of seven different subtypes with some overlap between them as detailed in Table 1. We have used ‘cleft lip phenotypes’ within the manuscript to refer to any subtype which includes individuals with a cleft lip. This is now clarified in the methods.

All biological samples from the Cleft Collective and the Millenium Cohort Study were sent to Bristol Bioresource Laboratories (https://www.bristol.ac.uk/population-health-sciences/research/groups/bblabs/) where they were stored and processed, and where genotyping was carried out [30, 91].

For information on DNA extraction and quantification, please see **Supplementary method**.

### Genotyping

Genotyping was performed on 200 ng of DNA using the Illumina Infinium global screening array-24 v1.0 in the Millennium cohort (606 434 SNPs) and using the Illumina Infinium global screening array-24 v3.0 in the Cleft Collective (645 027 SNPs). Different arrays were used for the cases and controls because genotyping was performed at a later date for the Cleft Collective. Quality control (QC) steps were performed and samples were excluded if they had a within sample SNP call rate < 97%, biological sex mismatch (between records and DNA) and low child–parent inter-relatedness based on identity by descent (IBD < 0.4). SNPs were excluded based on call rate < 98%, minor allele frequency (MAF) < 1%, Hardy–Weinberg equilibrium *P*-value <1 × 10^−6^ in controls or poor clustering and excess heterozygosity in cases (excess het > +/−0.3). After QC 457642 SNPs genotyped in controls and 480 916 SNPs genotyped in cases remained. Figure 1 shows the number of individuals excluded at each stage of the QC process and the number of included cases for each orofacial cleft subtype.

### Merged cases and controls

Cases and controls were merged and 399 882 SNPs common to both groups were retained. Principal components (PC) for ancestry were generated and applied to the combined group, and outlier filtering applied. Supplementary Fig. 1 shows the distribution of the included cases and controls in a scatter plot of PC1 versus PC2.

### Imputation

Imputation was performed on the merged case–control dataset using the TopMed Imputation Server to the TopMed R3 reference panel of 133 597 samples [92]. The imputation used Minimac4, and phasing with Eagle v2.4. Following imputation, SNPs were excluded if imputation quality RSq score < 0.3, MAF < 0.01, or they were monomorphic.

Following imputation, SNPs with call rate < 98% were identified and excluded (n = 166), and additional filtering was performed on SNPs with HWE *P* < 1 × 10^−6^ in controls (n = 399 457), leaving 7 606 581 SNPs which passed our quality control steps. After excluding SNPs with a minor allele frequency of < 0.05, a maximum of 5 457 364 SNPs were included in our analyses.

### Statistical analysis

Genome-wide association analyses were conducted comparing cases with controls. Initially, all cleft subtypes were combined to maximize statistical power. Subsequently, GWAS were performed for individual cleft subtypes as defined above. Whilst we used a common set of controls across all our analyses the number of cases varied in the different analyses (see Fig. 1 for number of cases in each subtype). Finally, we explored the extent to which genetic variants are shared between the subtypes and the heterogeneity between them. PLINK v1.9 [93] was used to perform genome-wide association tests.

Allele frequency differences between cases and controls were tested using logistic regression adjusted for scores of the first 10 principal components generated from our merged case–control pre-imputed data. SNPs were included if MAF > 0.05 in the specific phenotype subset. This analysis was performed using data imputed using the TopMed R3 reference panel [92].

#### Subtype comparisons

Results for any SNP that was genome-wide significant (5 × 10^−8^) in at least one subgroup were reported across all subgroups. We did not make any adjustment for multiple testing due to the inclusion of multiple subgroups because the subgroups were not independent of each other. However, we examined the differences in p-values and effect estimates between subgroups, and plotted these as a forest plot as part of our analysis. We also reported the results for the orofacial cleft subgroup with the lowest p-value for each SNP, and listed any other subgroups with *P*-values of < 1 × 10^−4^ in Table 2. We relaxed our threshold for reporting associations with secondary phenotypes because some subgroups had smaller numbers and hence lower power to detect similar effects. We conducted pairwise comparisons across subtypes by calculating Pearson's correlation coefficients using effect estimates for any clumped SNP that was GWAS significant for at least one cleft subtype. We also performed a meta-analysis and used a Cochrane’s Q-test to test whether there was evidence for heterogeneity between cleft lip only (CLO) and cleft lip and palate (CLP) subtypes for the following SNPs: rs1347188, rs62390705, rs1854110 which appeared to show a difference in associations between the two subtypes.

#### Replication

A look-up was performed by extracting the results for the lead SNPs for each genome-wide significant locus in our analyses from a recent independent meta-analysis of non-syndromic cleft lip with or without cleft palate (nsCL/P) and we reported the results alongside our results. The meta-analysis combined case–control and trio data from 3 separate studies of nsCL/P [12, 13, 16] comprising of individuals of European, Asian and Latin American ancestry. The final dataset comprised 1247 nsCL/P cases, 2879 controls, 2699 case-parent trios, and approximately 7.74 million SNPs. We reported the p-value of the association of the SNP with nsCL/P in our replication sample alongside our main results. Where the SNP effect was in the same direction and with a *P*-value < 0.05 we considered this a positive replication.

#### Follow-up analyses

There were 8 sets of summary statistics generated by the GWAS of the 8 phenotypes. For each set of results, Manhattan and QQ plots were generated in R version 4.3.1 using the package *qqman* [94]. QQ plots were used to assess inflation, along with lambda estimates. Genome-wide significant SNPs (*P* < 5 × 10^−8^) were identified, and genetically independent locations identified using the package *ieugwasr* [95] to clump SNPs with a clumping window of 250 kb and r2 cutoff of 0.2.

Summary statistics were uploaded to FUMA SNP2GENE [96] for functional mapping and annotation, and LocusZoom [97] to generate plots of locations with genome-wide significant associations. On visual inspection of the locus zoom plots, some clumped regions were merged or separated. The lead SNP in each region was reported as the one with the smallest p-value. The 95% credible region was also noted. Mapped genes were either genes which contained the SNP or the gene which was closest to the SNP according to dbSNP (https://www.ncbi.nlm.nih.gov/projects/SNP/).

Clumped SNPs, their mapped genes and a region of 500 000 bases either side of the location of the SNP were searched in GWAS catalog (GWAS Catalog) and any associations with orofacial clefts were noted. We also used https://www.geneontology.org/ to investigate whether there were any biological processes which were over-represented among the mapped genes.

Finally, the measured SNP-based heritability was quantified for each analysis, by LD score regression [98]. This involved regressing the SNP-level summary statistics for a phenotype against the standardised sum of LD.

## Supplementary Material

Supplementary_Figures_revised_ddaf131

Supplementary_tables_-_cleft_GWAS_final_ddaf131

Supplementary_Methods_ddaf131
