# Supplementary material for: Genetic heterogeneity and homogeneity among orofacial cleft subtypes: genome-wide association studies in the cleft collective
Source: Hum Mol Genet. 2025 Oct 11;34(23):1934–50. doi: 10.1093/hmg/ddaf131 (PMC12627943; doi:10.1093/hmg/ddaf131)

Supplementary Figure 1 – scatter plot showing principal component 1 versus principal component 2 for ancestry in cases versus controls


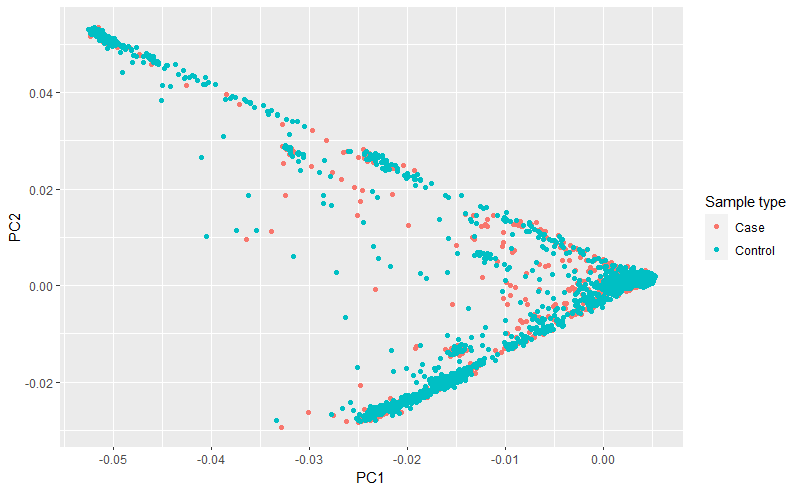


Supplementary Figure 2 – Manhattan plot – All cases


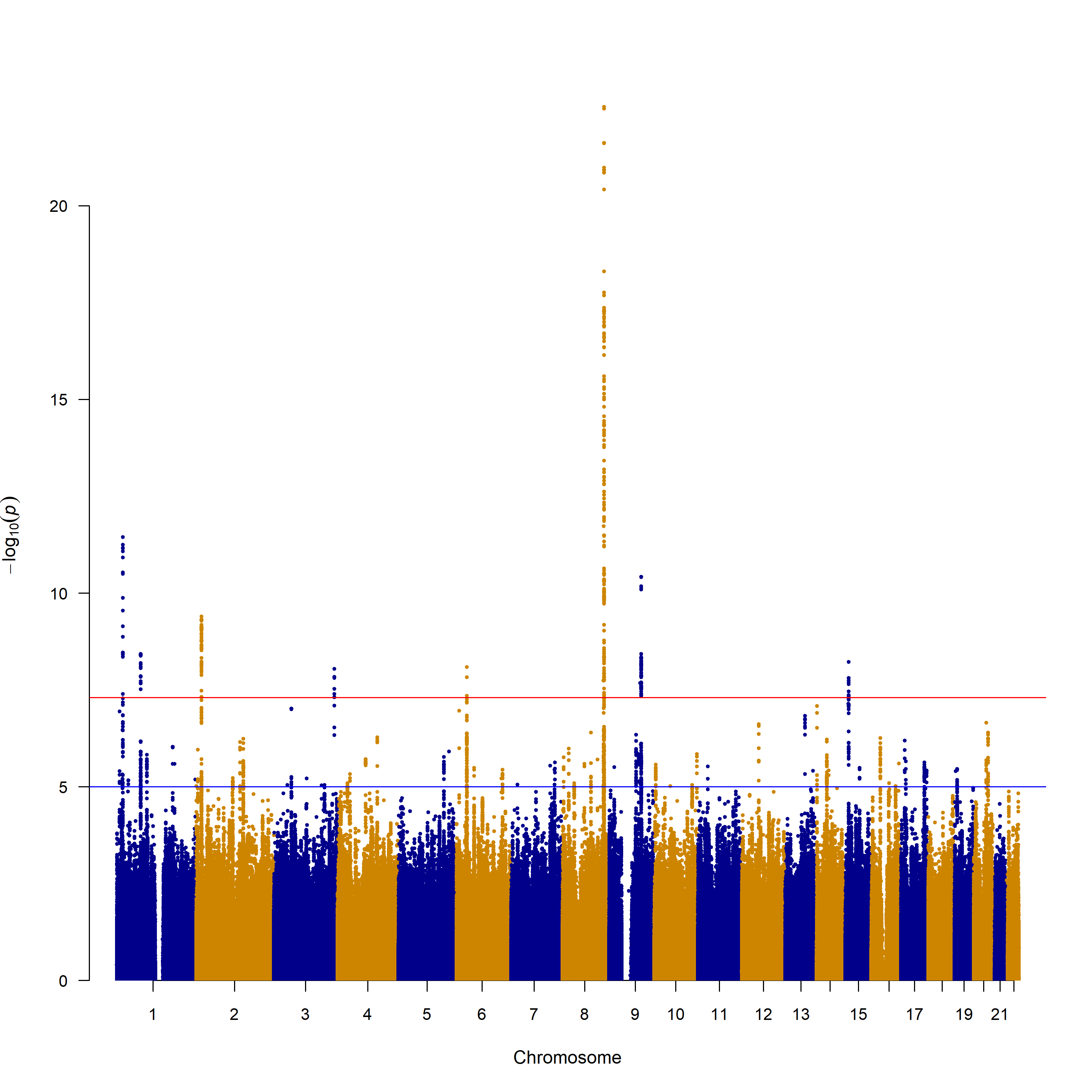


Supplementary Figure 3 – Quantile-Quantile plot – All cases


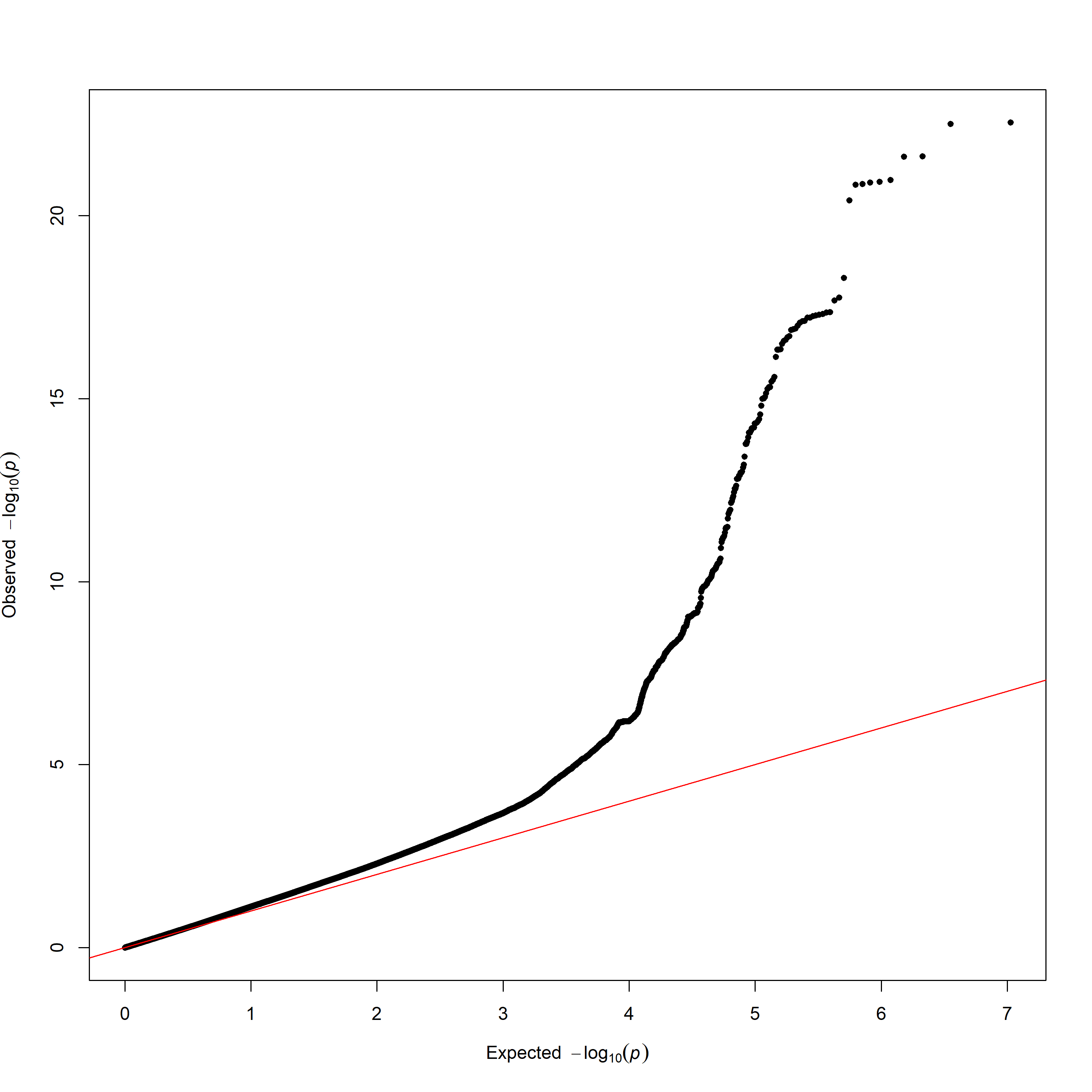


Supplementary Figure 4 - Locus Zoom Plot of region in 1p31.1 (lead SNP rs4112328) - all cleft cases


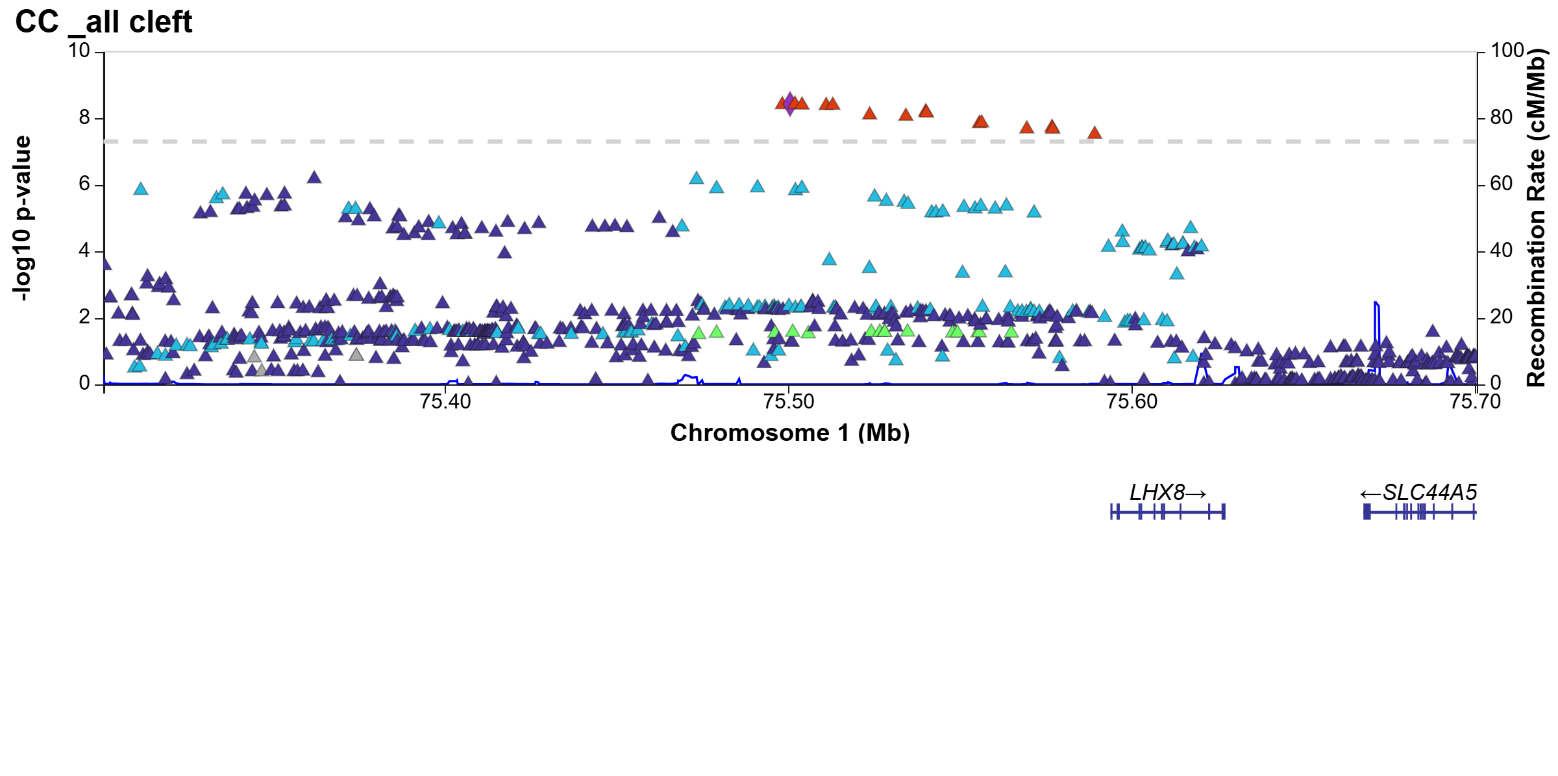


Supplementary Figure 5 - Locus Zoom Plot of region in 6p21.32 (lead SNP rs28361060) - all cleft cases


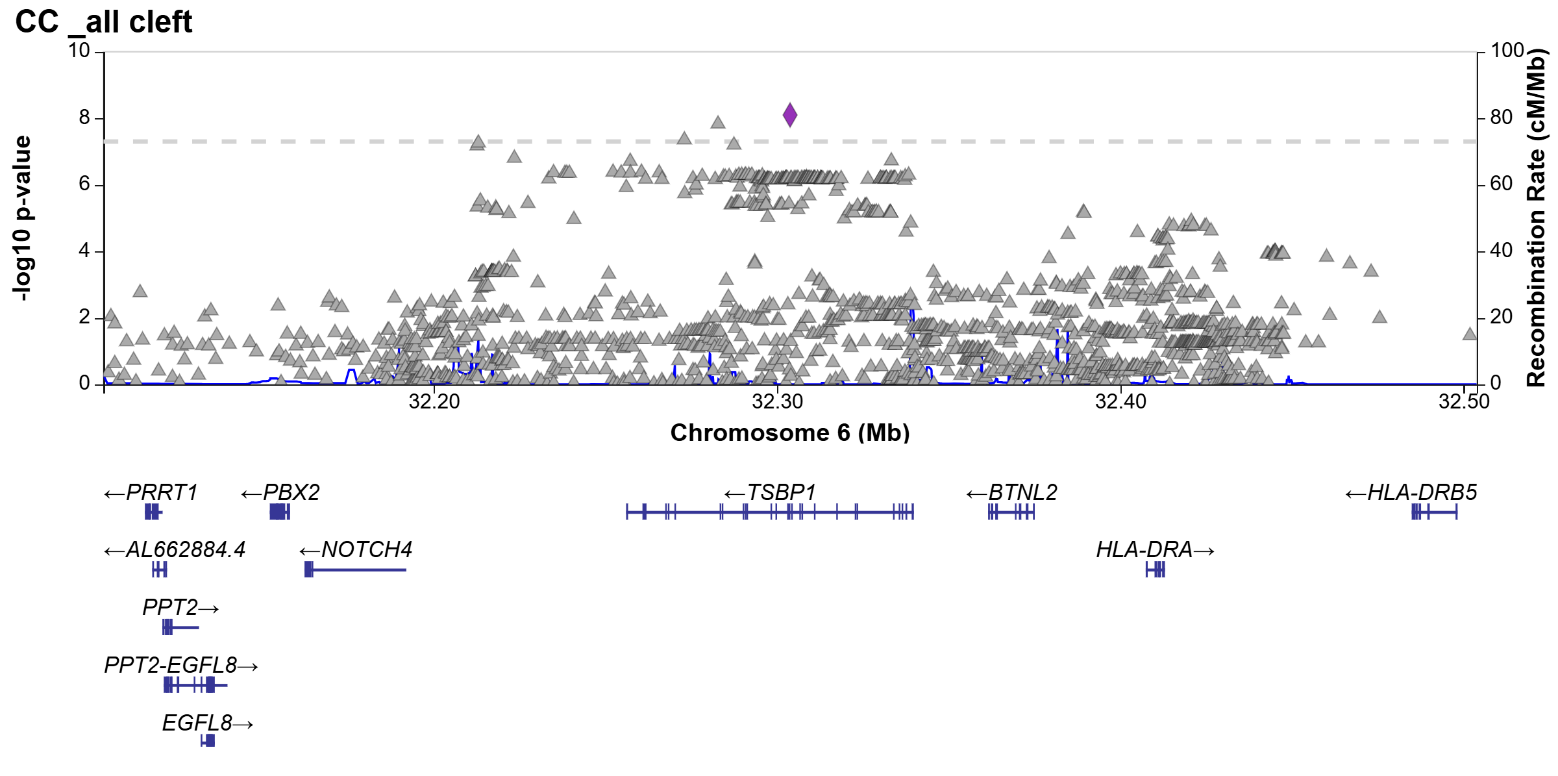


Supplementary Figure 6 - Locus Zoom Plot of region in 9q22.33 (lead SNP) rs7870795 - all cleft cases


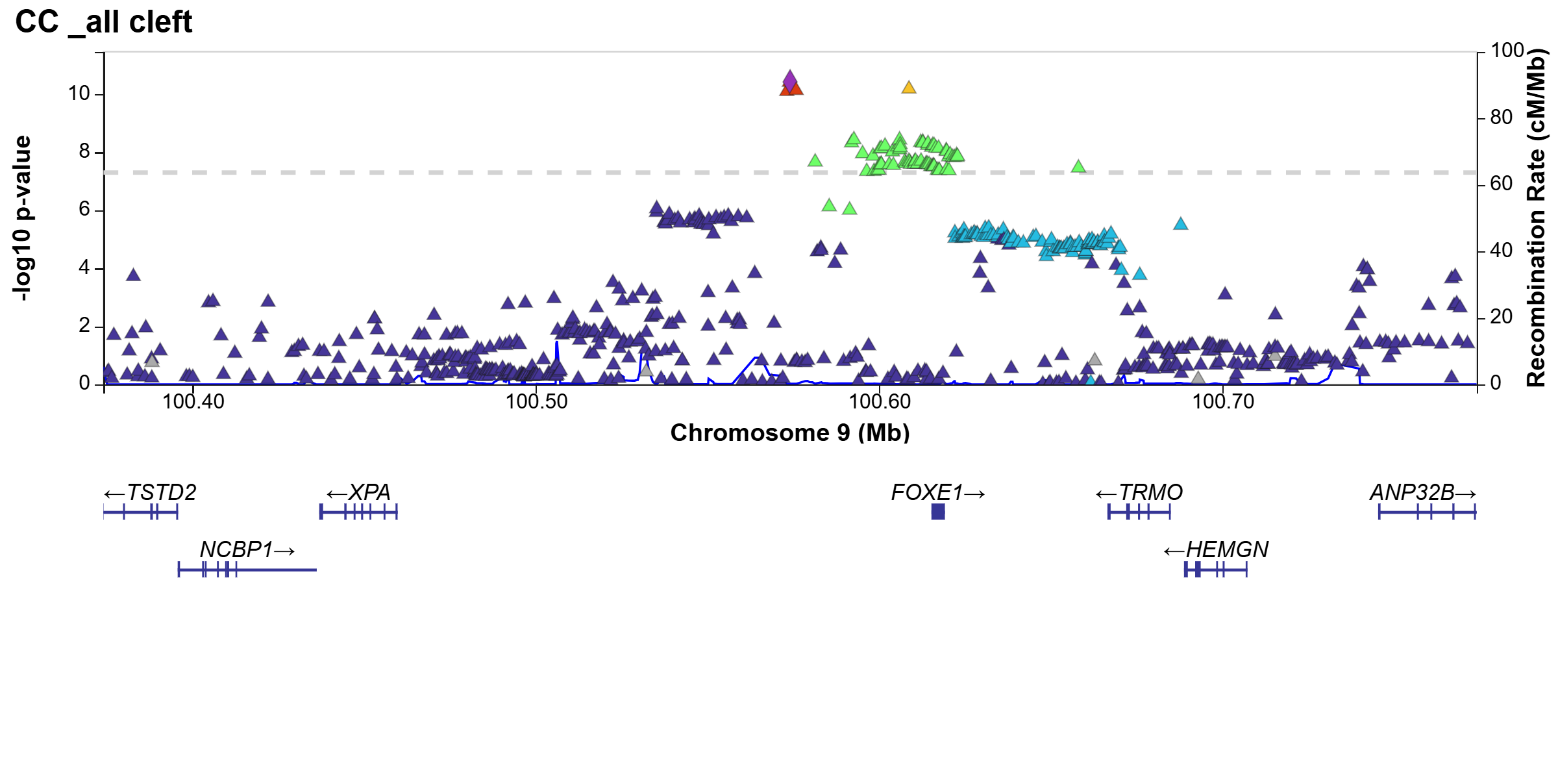


Supplementary Figure 7 – Manhattan plot – Cleft lip with or without cleft palate


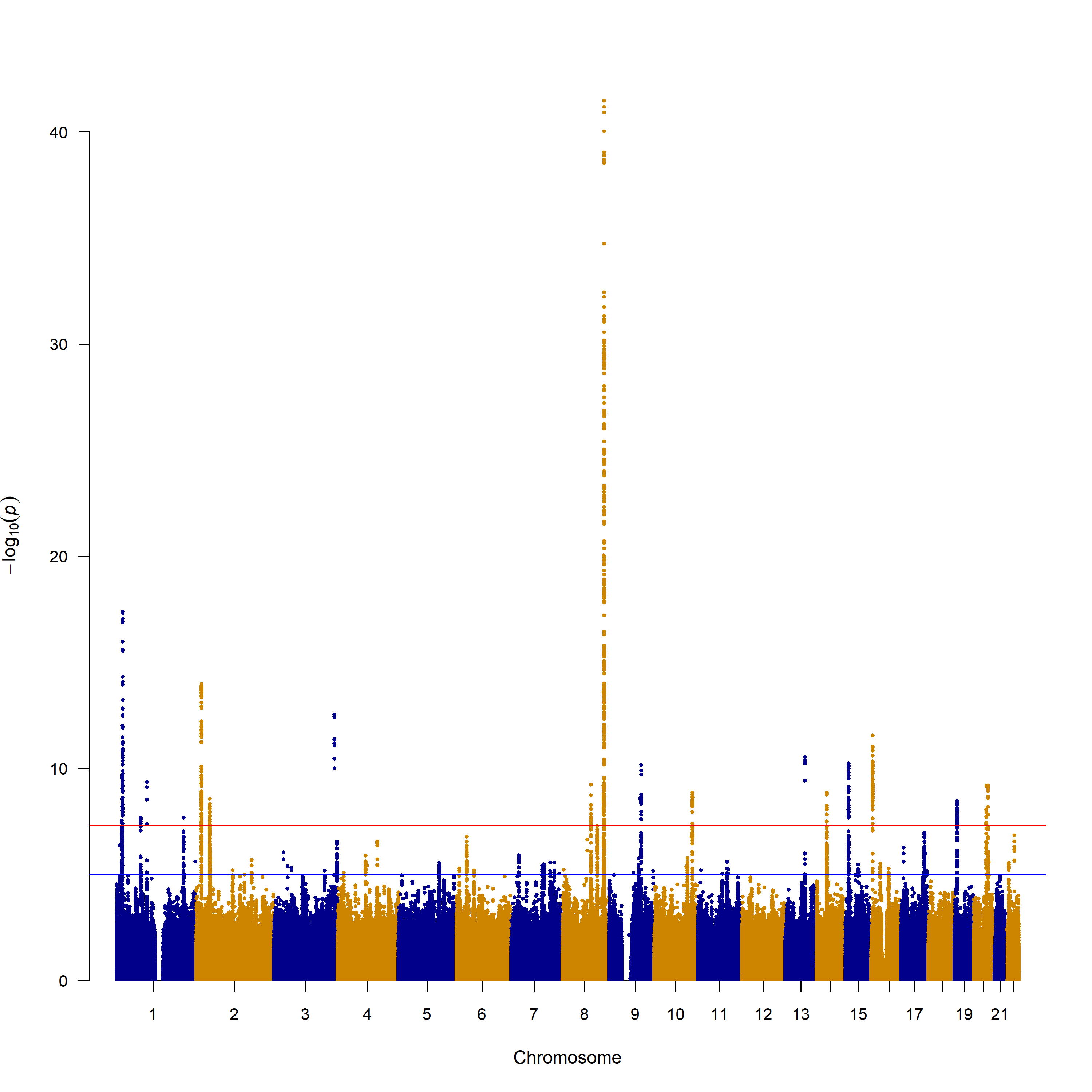


Supplementary Figure 8– Quantile-Quantile plot – Cleft lip with or without palate


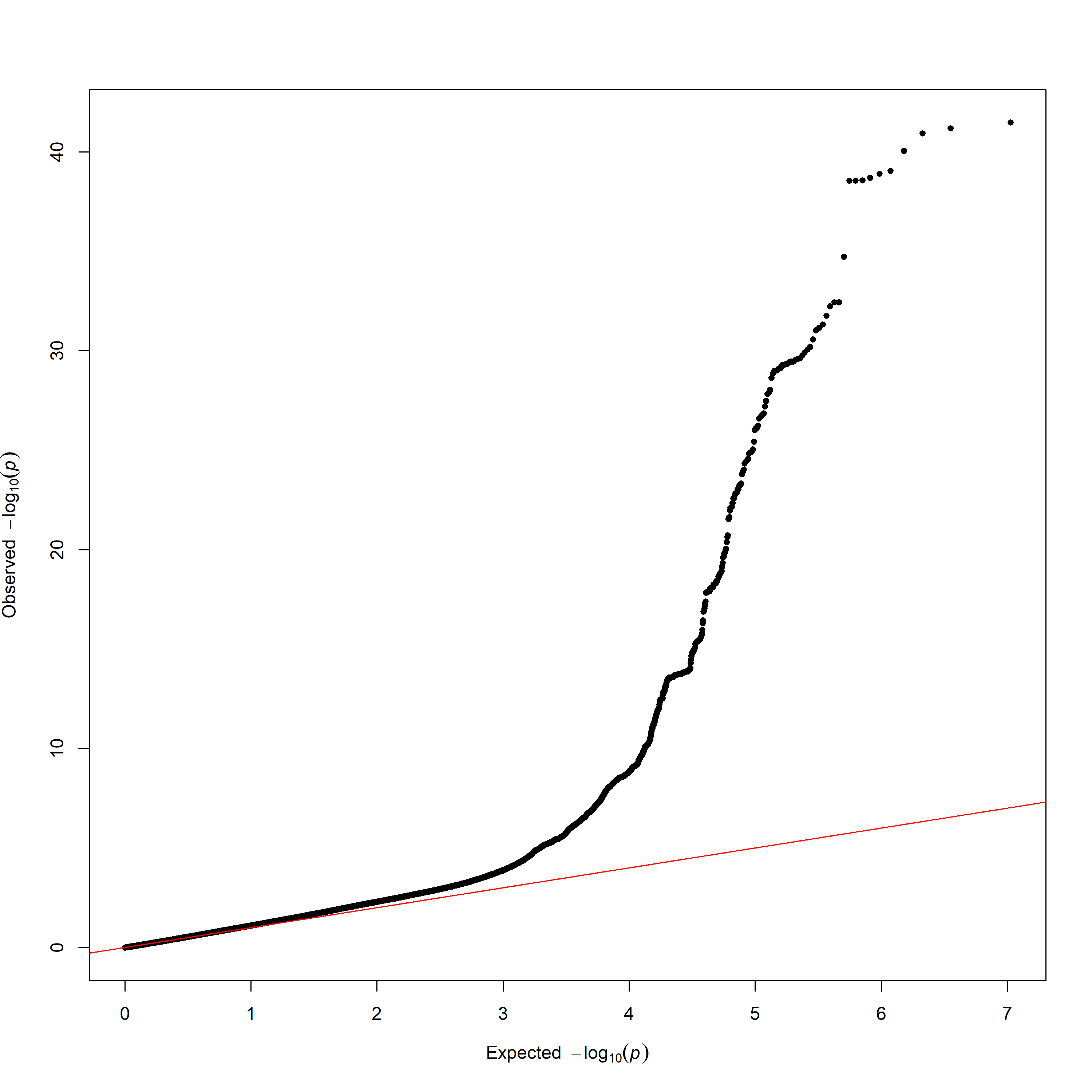


Supplementary Figure 9 - Locus Zoom Plot of region in 1p36.13 (lead SNP rs9439714) – Cleft lip with or without palate


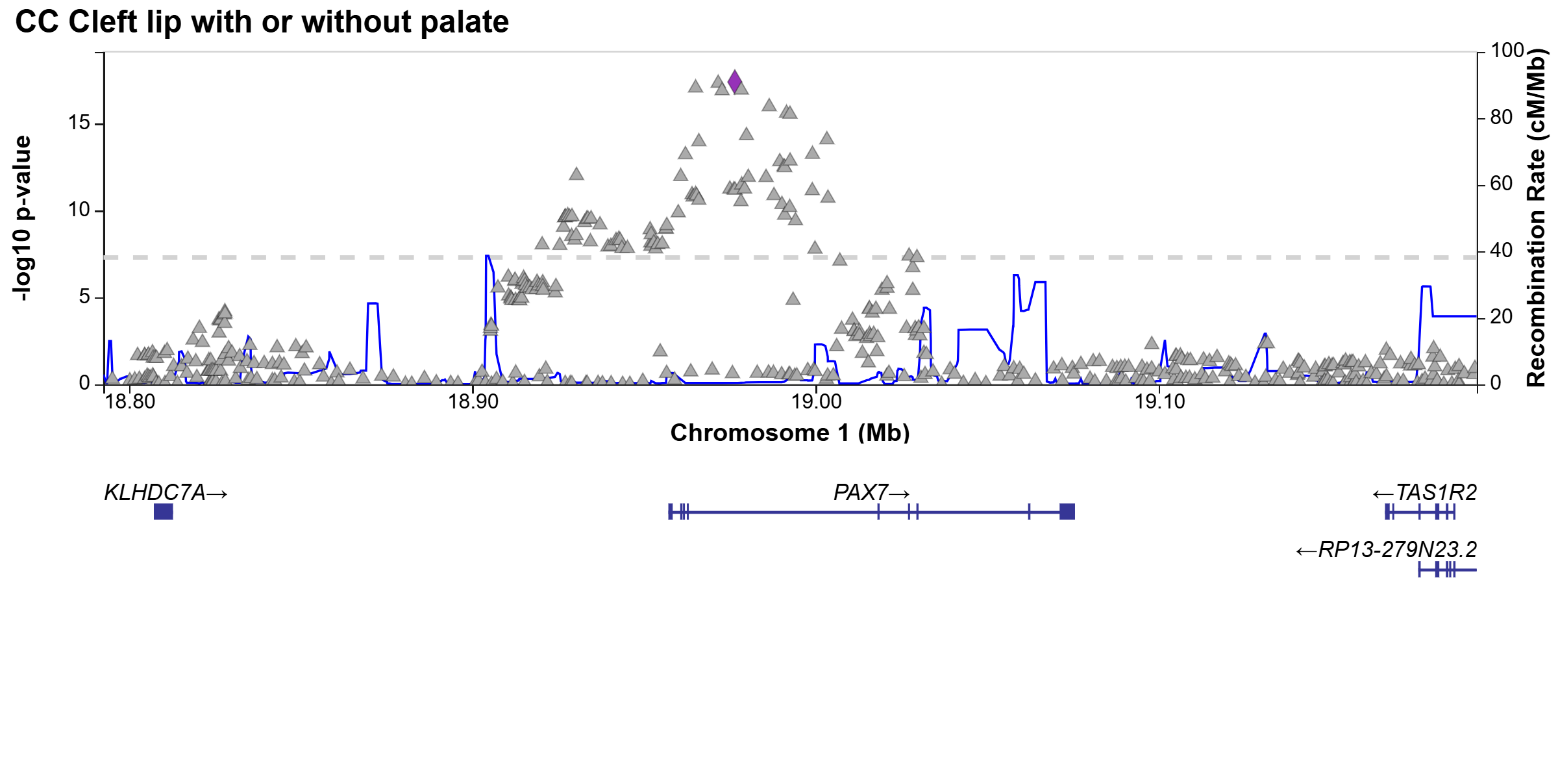
Supplementary Figure 10 - Locus Zoom Plot of region in 1p22.1 (lead SNP rs66515264) – Cleft lip with or without palate


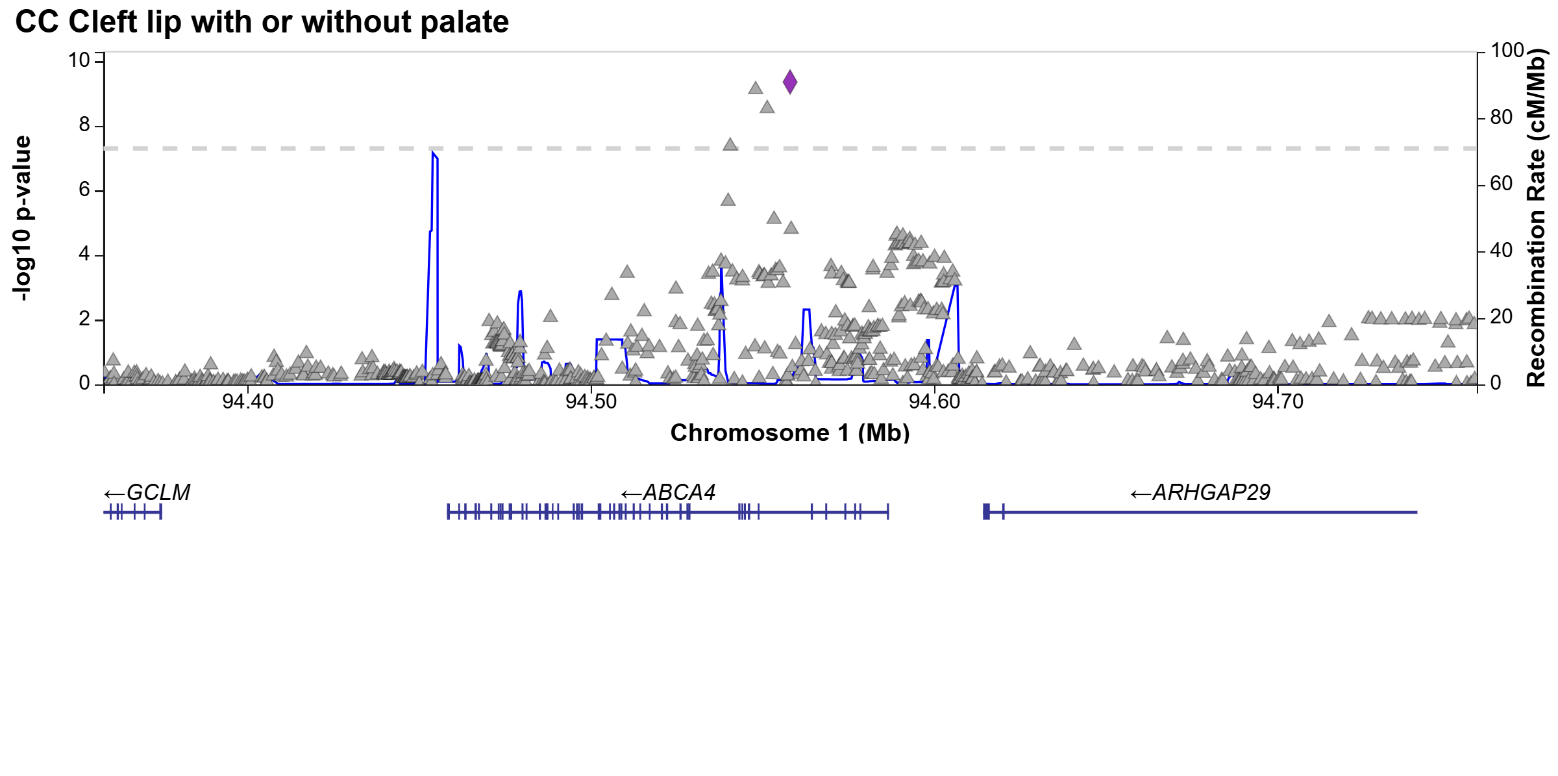


Supplementary Fig 11 - Locus Zoom Plot of region in 2p24.2 (lead SNP rs13385292) - Cleft lip with or without palate


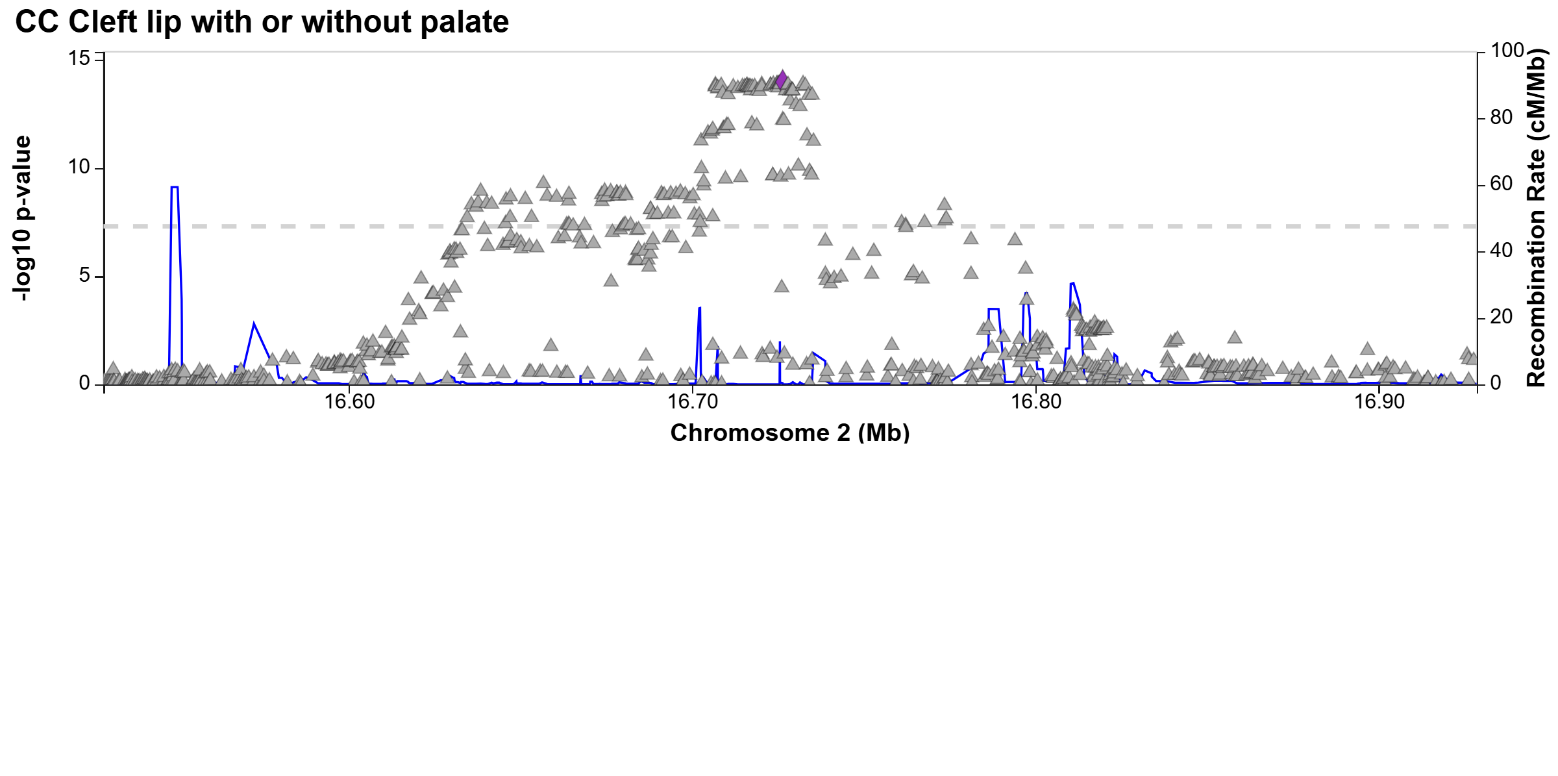
Supplementary Fig 12 - Locus Zoom Plot of region in 2q14.2 (lead SNP rs4952552) - Cleft lip with or without palate


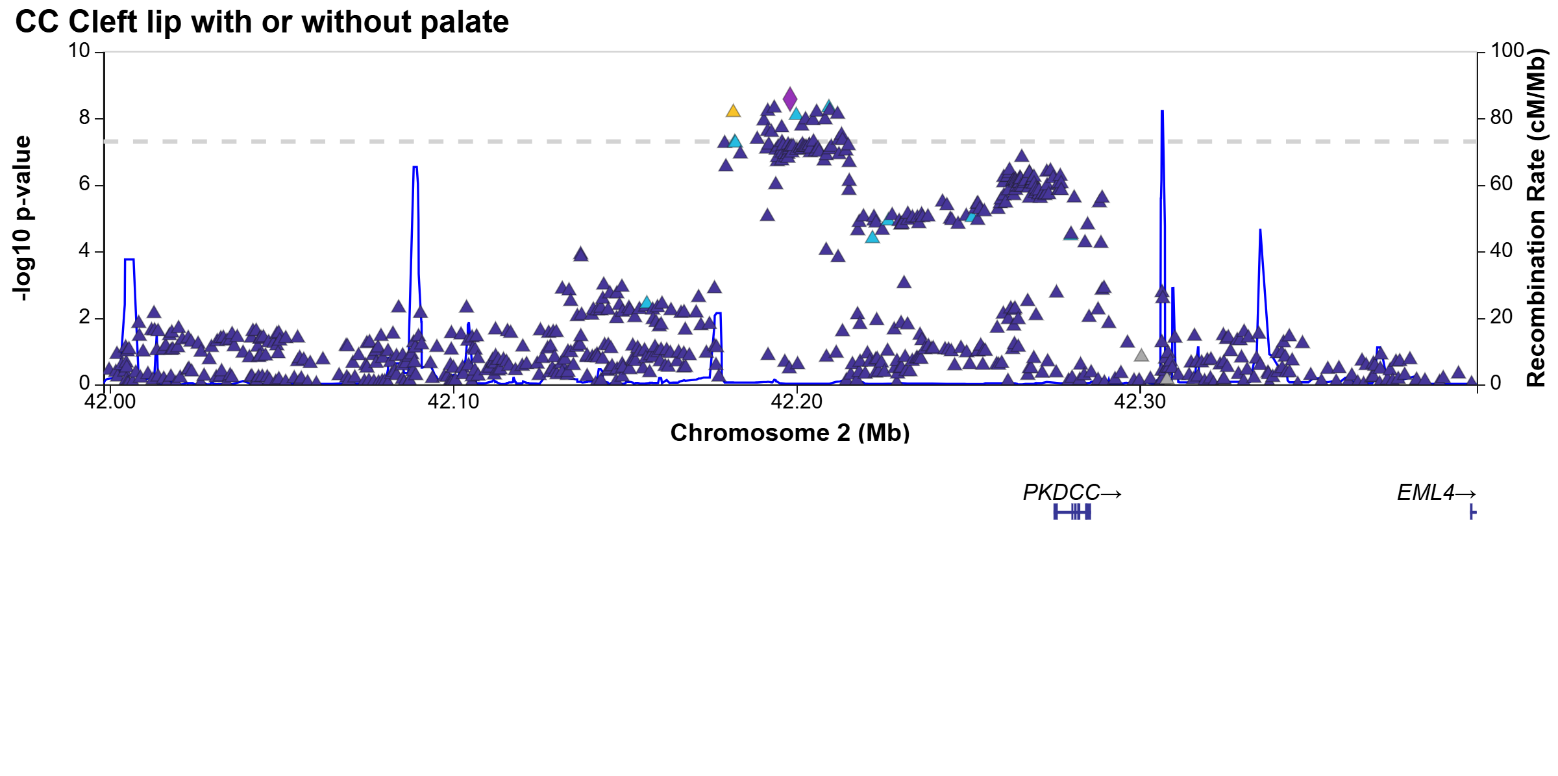


Supplementary Fig 13 - Locus Zoom Plot of region in 3q28 (lead SNP rs79482068) - Cleft lip with or without palate


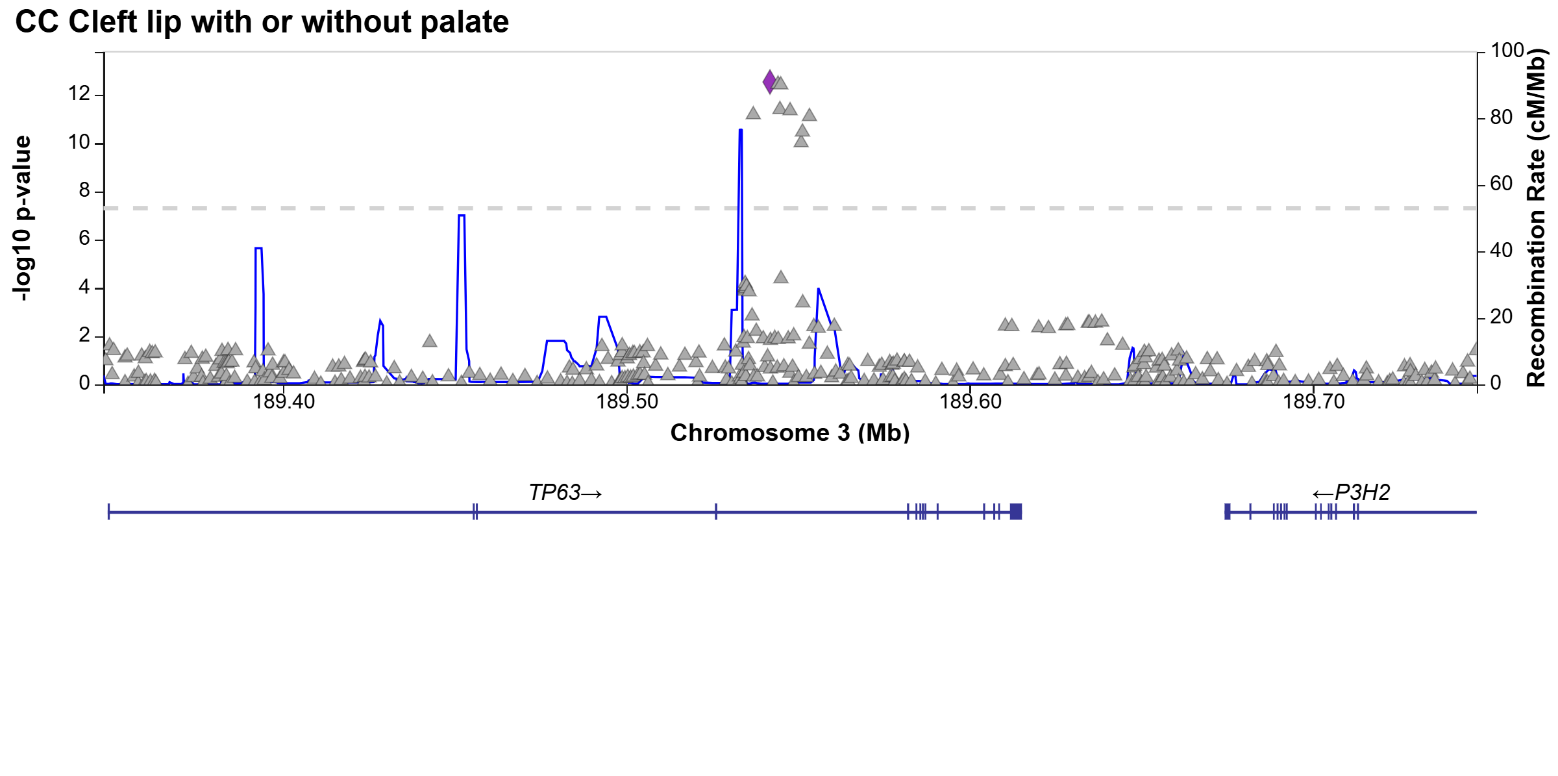
Supplementary Fig 14 - Locus Zoom Plot of region in 8q21.3 (lead SNP rs13385292) - Cleft lip with or without palate


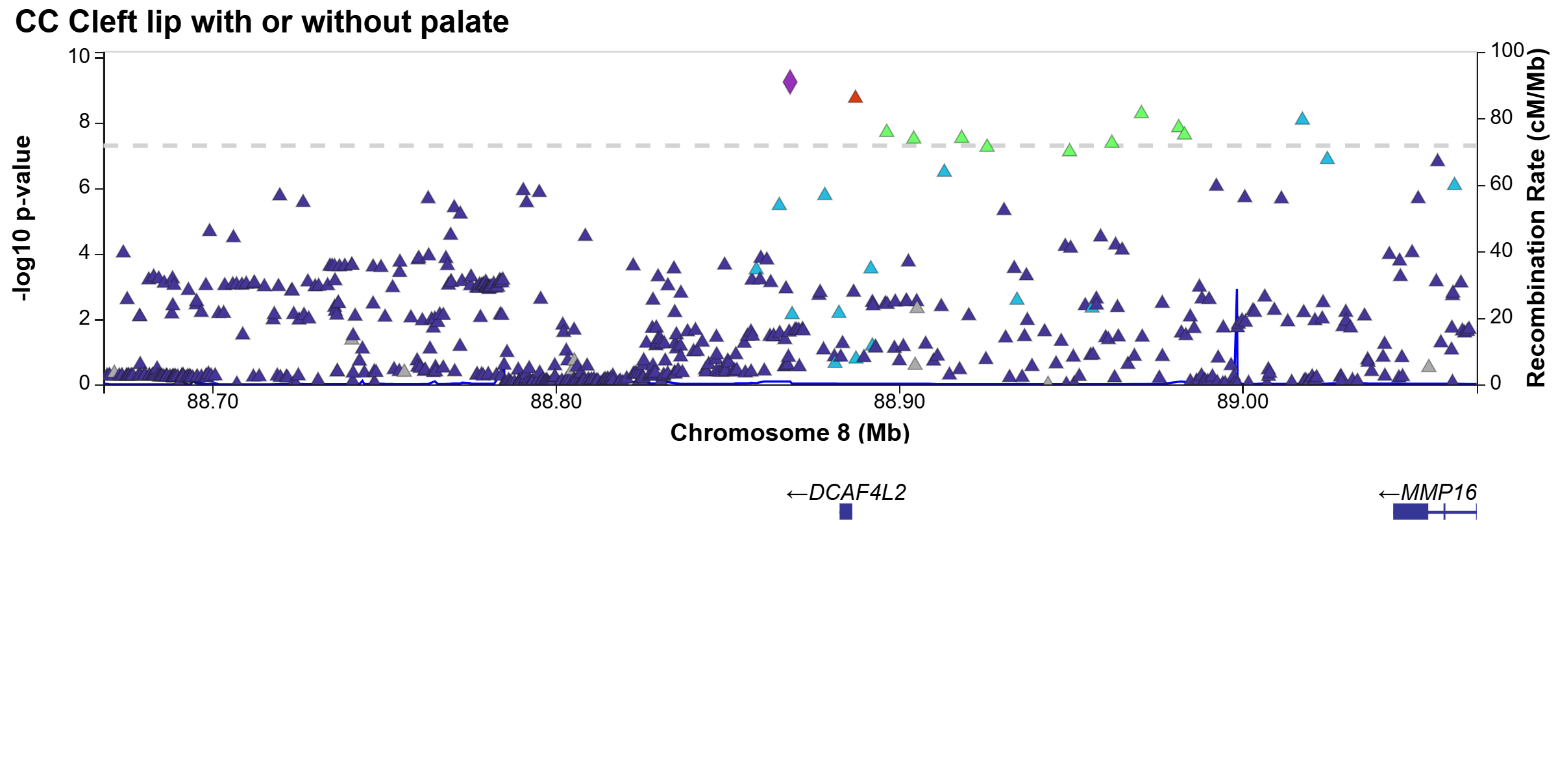


Supplementary Fig 15 - Locus Zoom Plot of region in 8q24.21 (lead SNP rs17242358) - cleft lip with or without palate


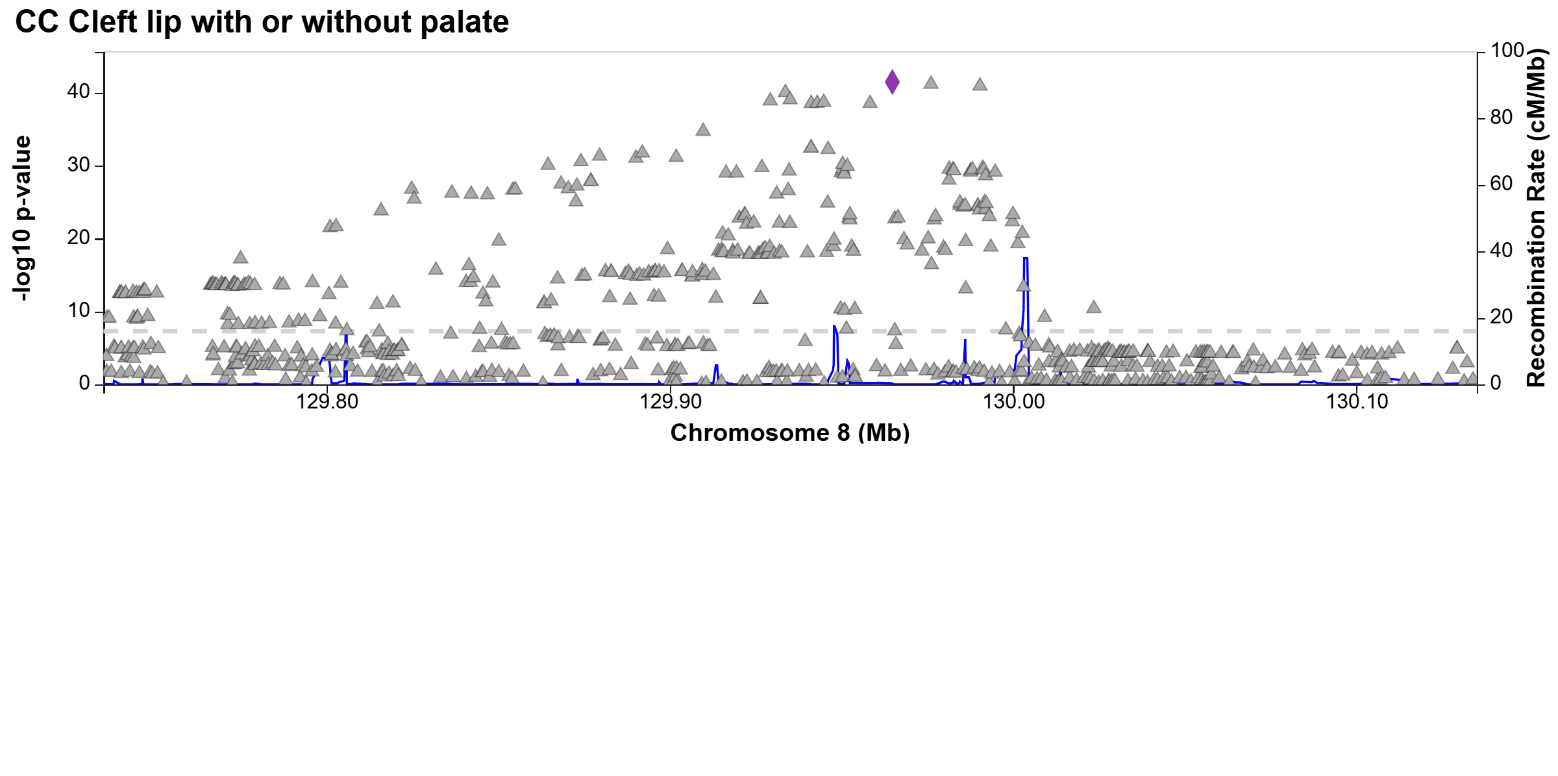
Supplementary Fig 16 - Locus Zoom Plot of region in 10q25.3 (lead SNP rs1898349) - cleft lip with or without palate


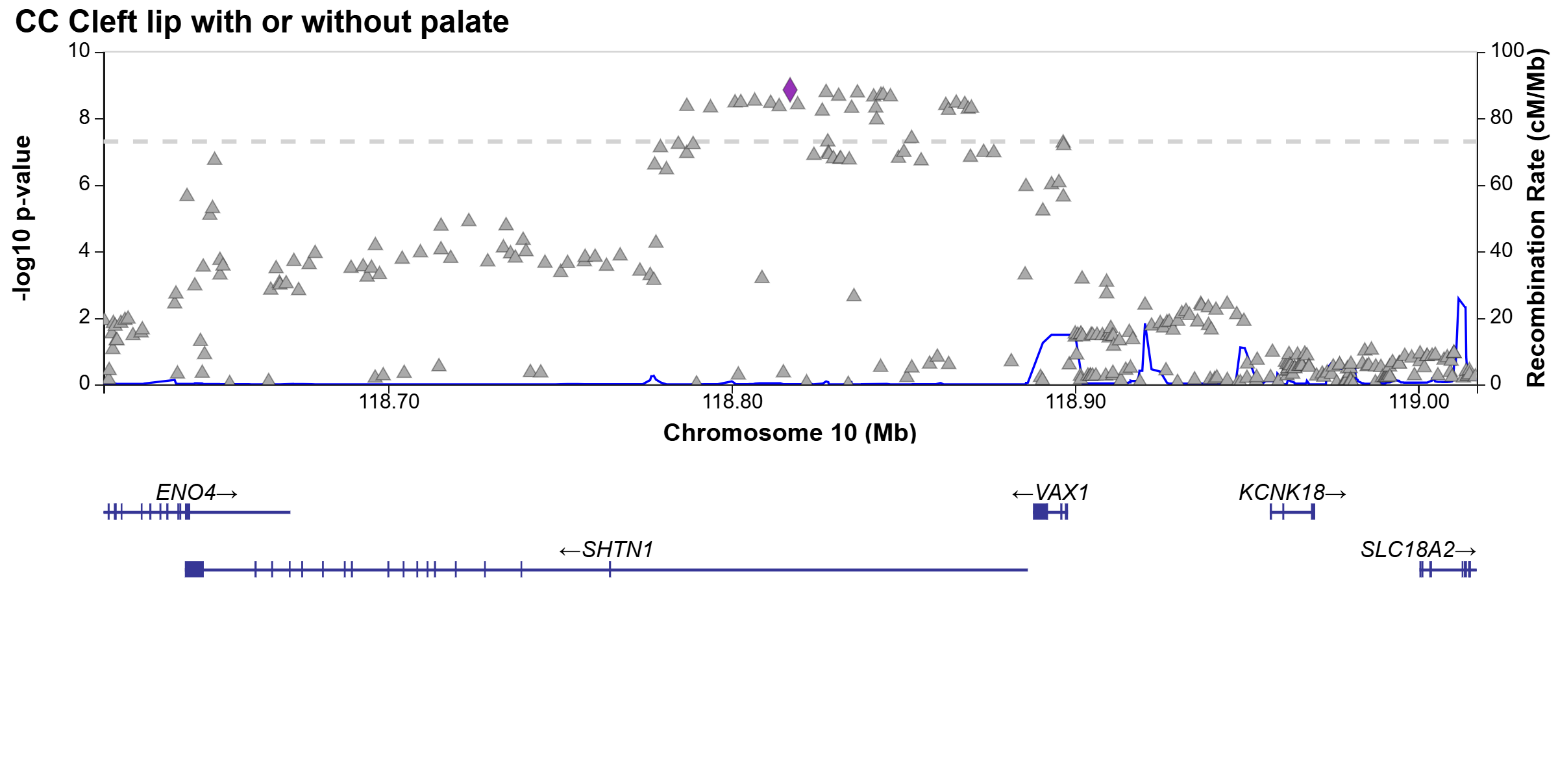


Supplementary Fig 17 - Locus Zoom Plot of region in 14q22.1 (lead SNP rs10483604) - cleft lip with or without palate


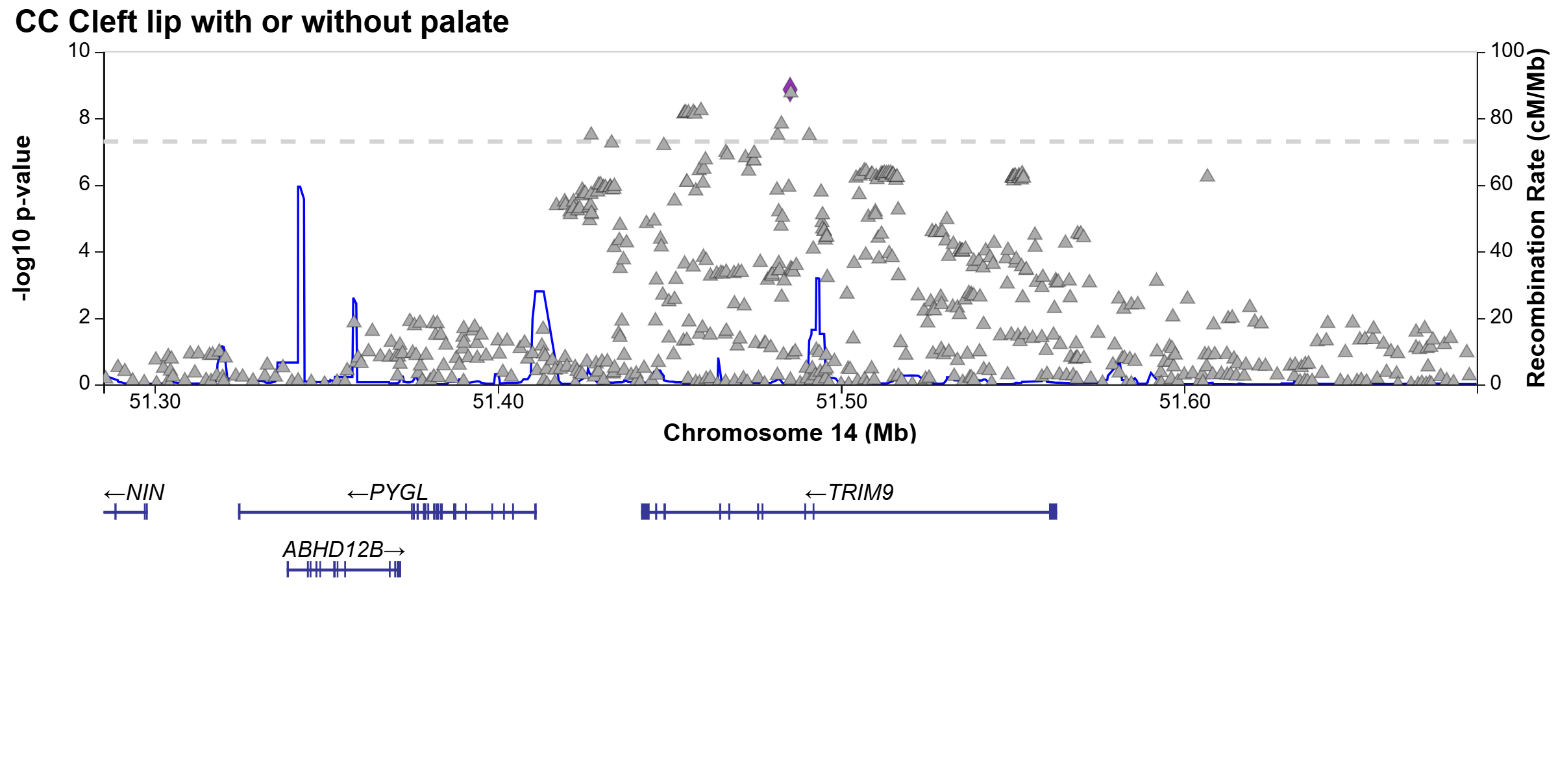


Supplementary Figure 18 - Locus Zoom Plot of region in 14q22.1 (lead SNP rs4901118) - cleft lip with or without palate


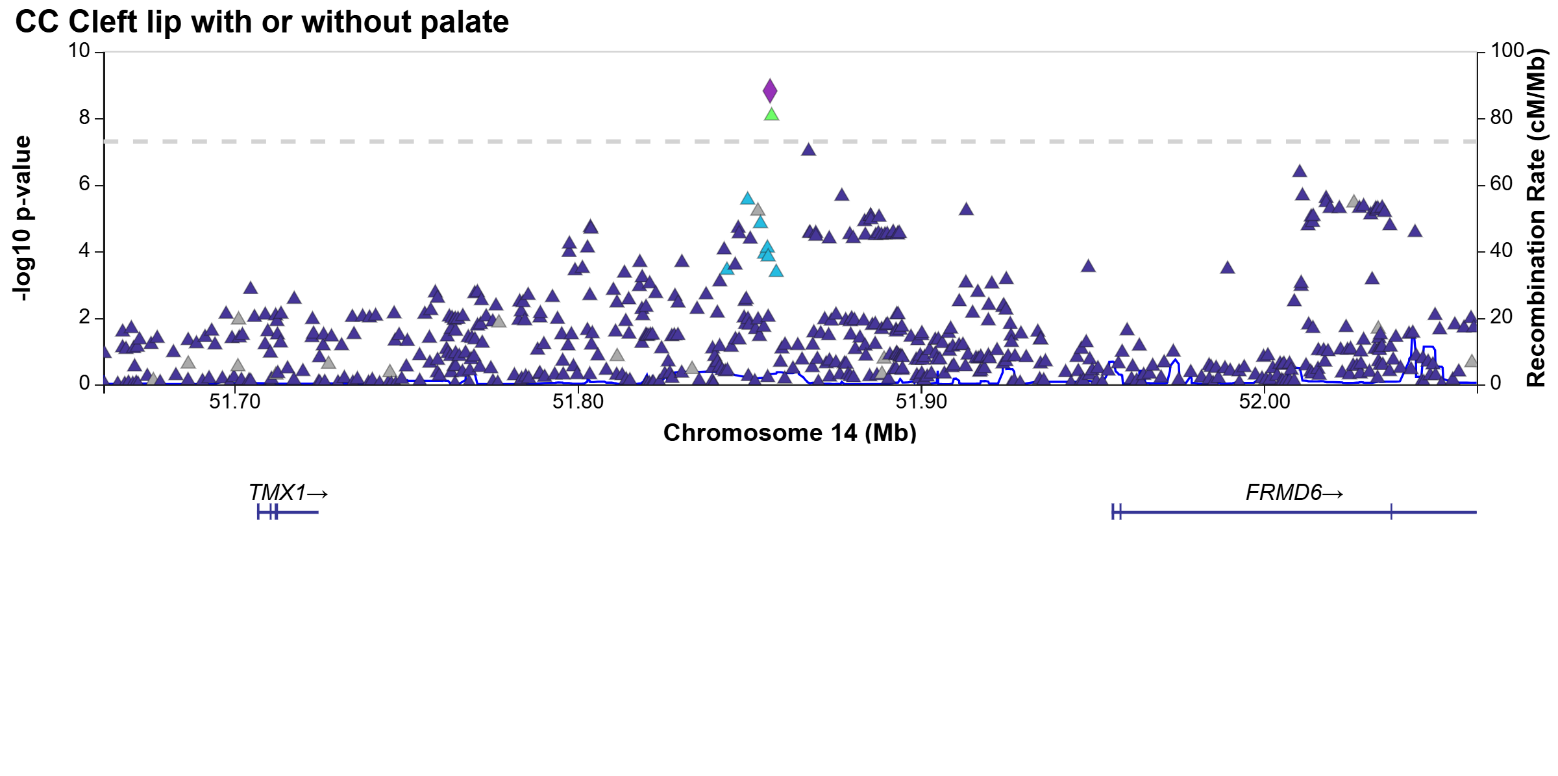


Supplementary Figure 19 - Locus Zoom Plot of region in 15q13.3 (lead SNP rs2600519) - cleft lip with or without palate


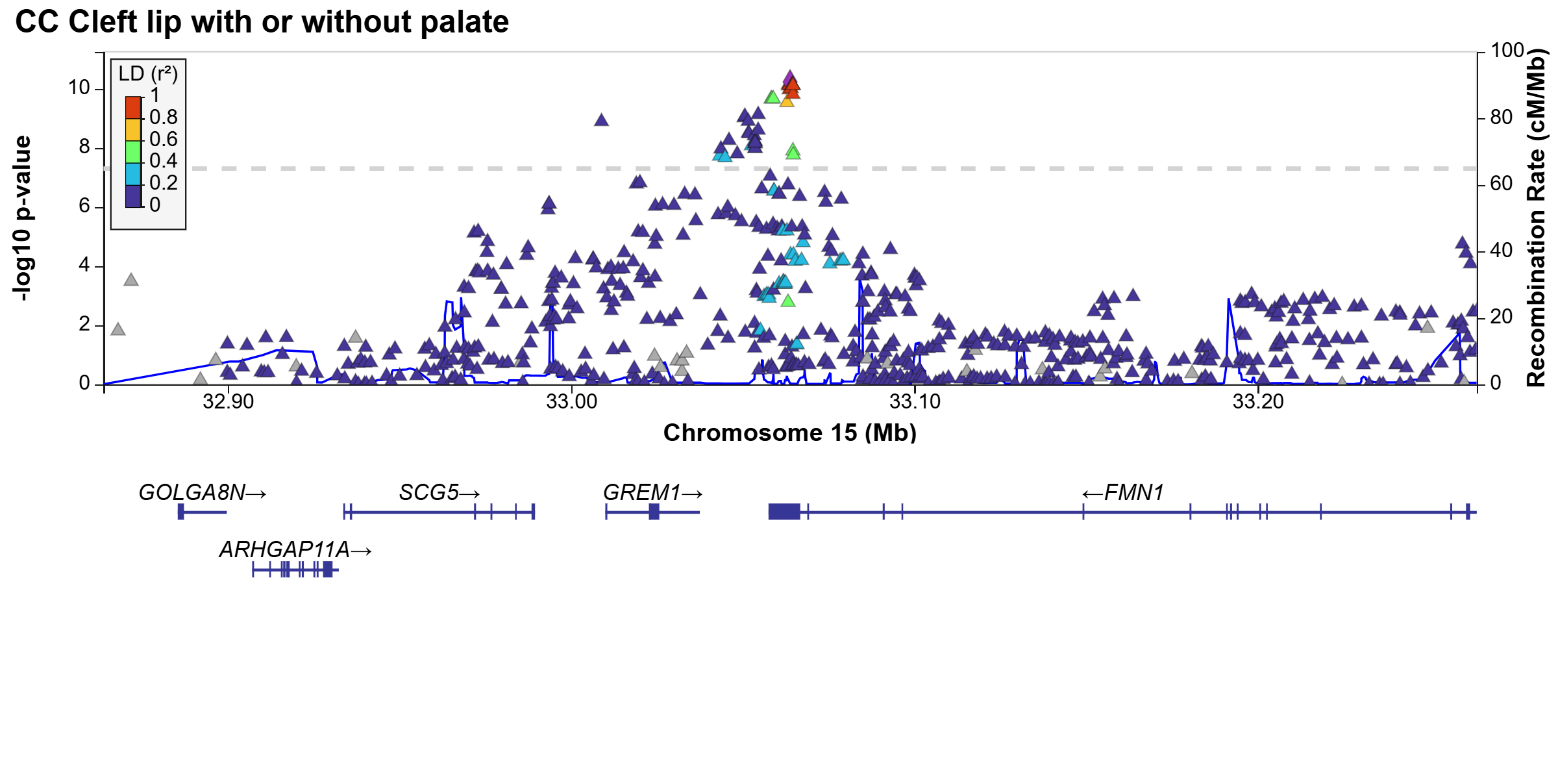


Supplementary Figure 20 - Locus Zoom Plot of region in 16p13.3 (lead SNP rs8044196) - cleft lip with or without palate


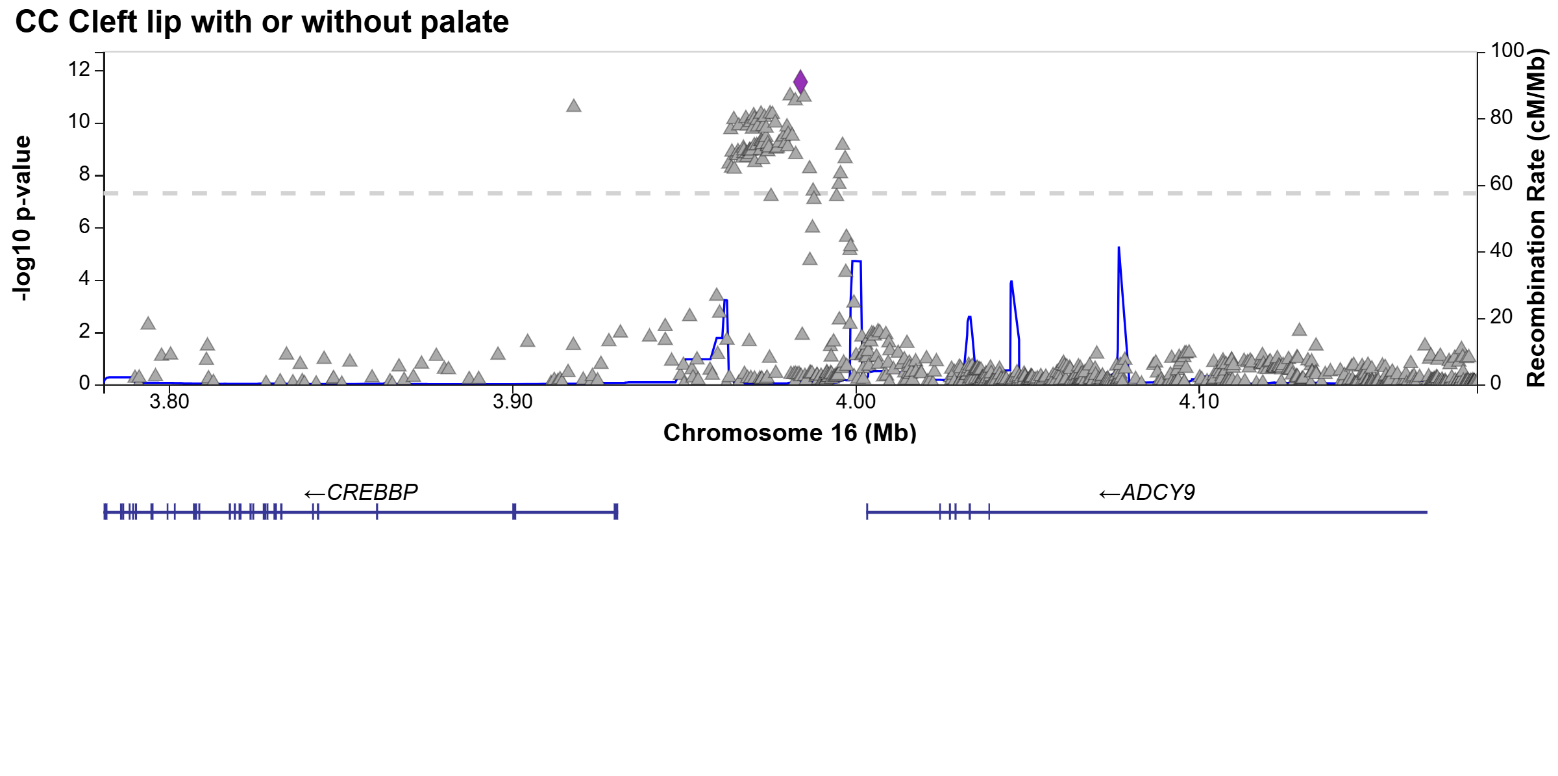


Supplementary Figure 21 - Locus Zoom Plot of region in 19p13.2 (lead SNP rs12971753) - cleft lip with or without palate


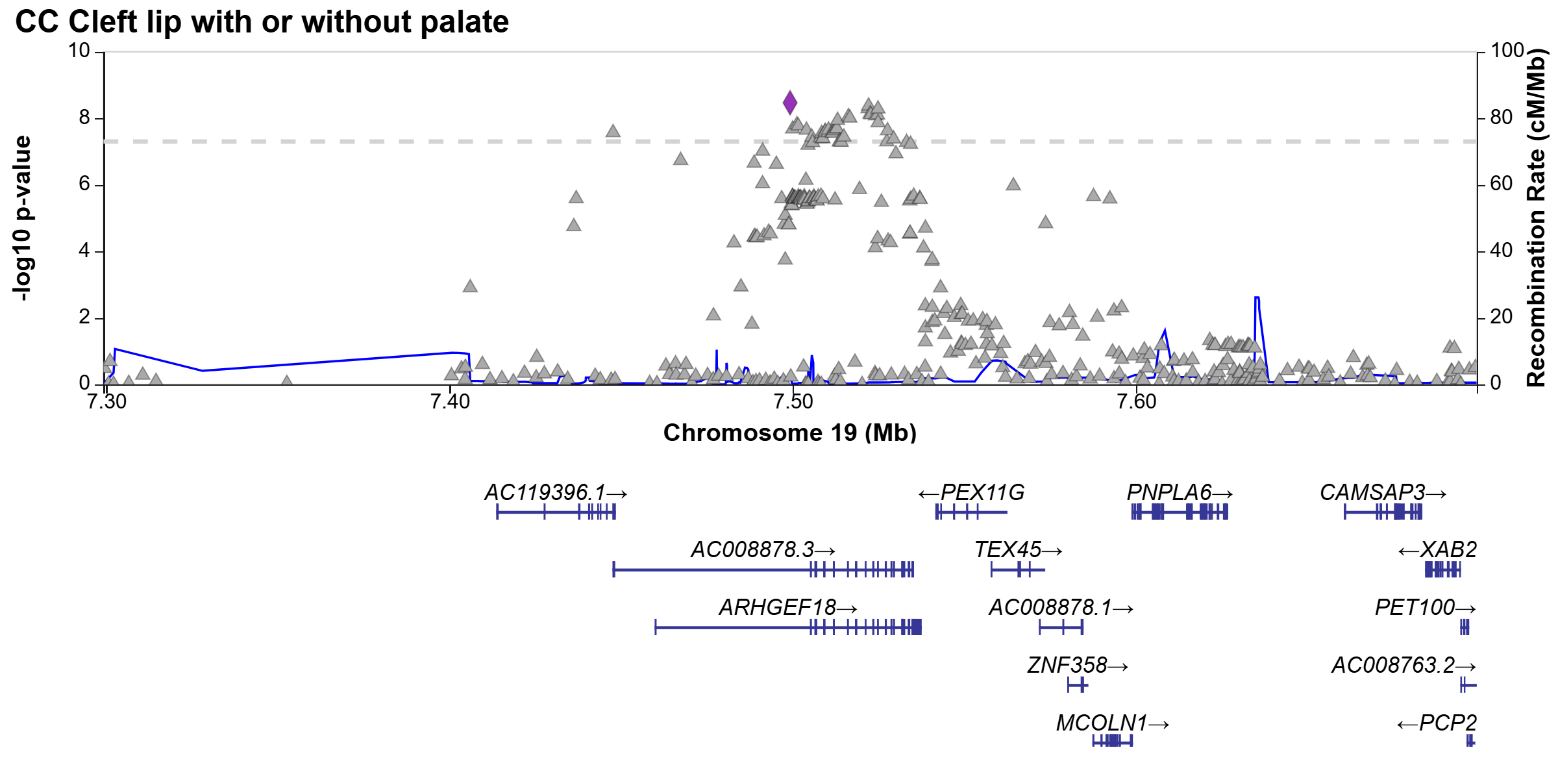


Supplementary Figure 22 - Locus Zoom Plot of region in 20q12 (lead SNP rs34753522) - cleft lip with or without palate


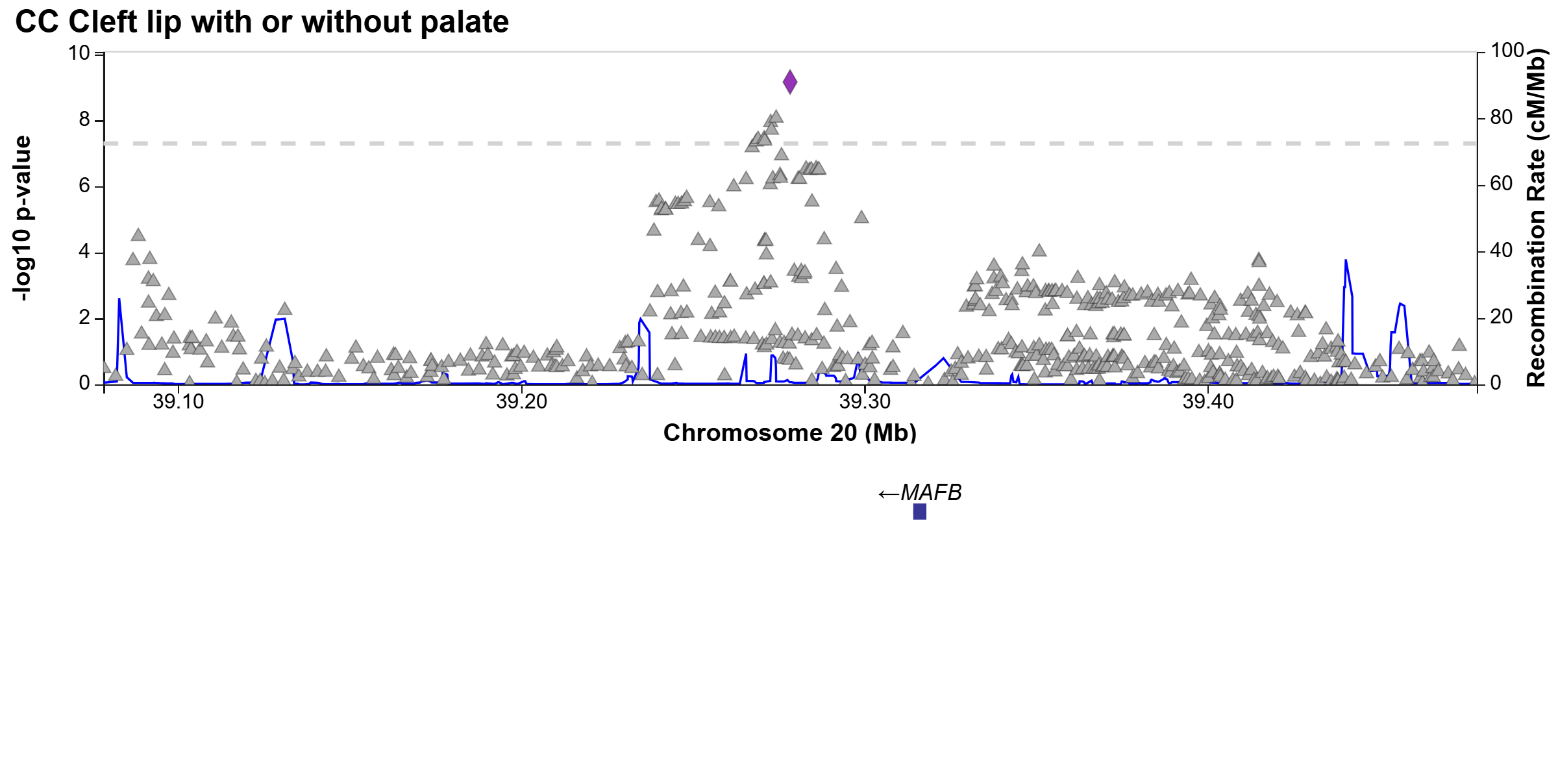


Supplementary Figure 23 - Locus Zoom Plot of region in 20q13.12 (lead SNP rs6018099) - cleft lip with or without palate


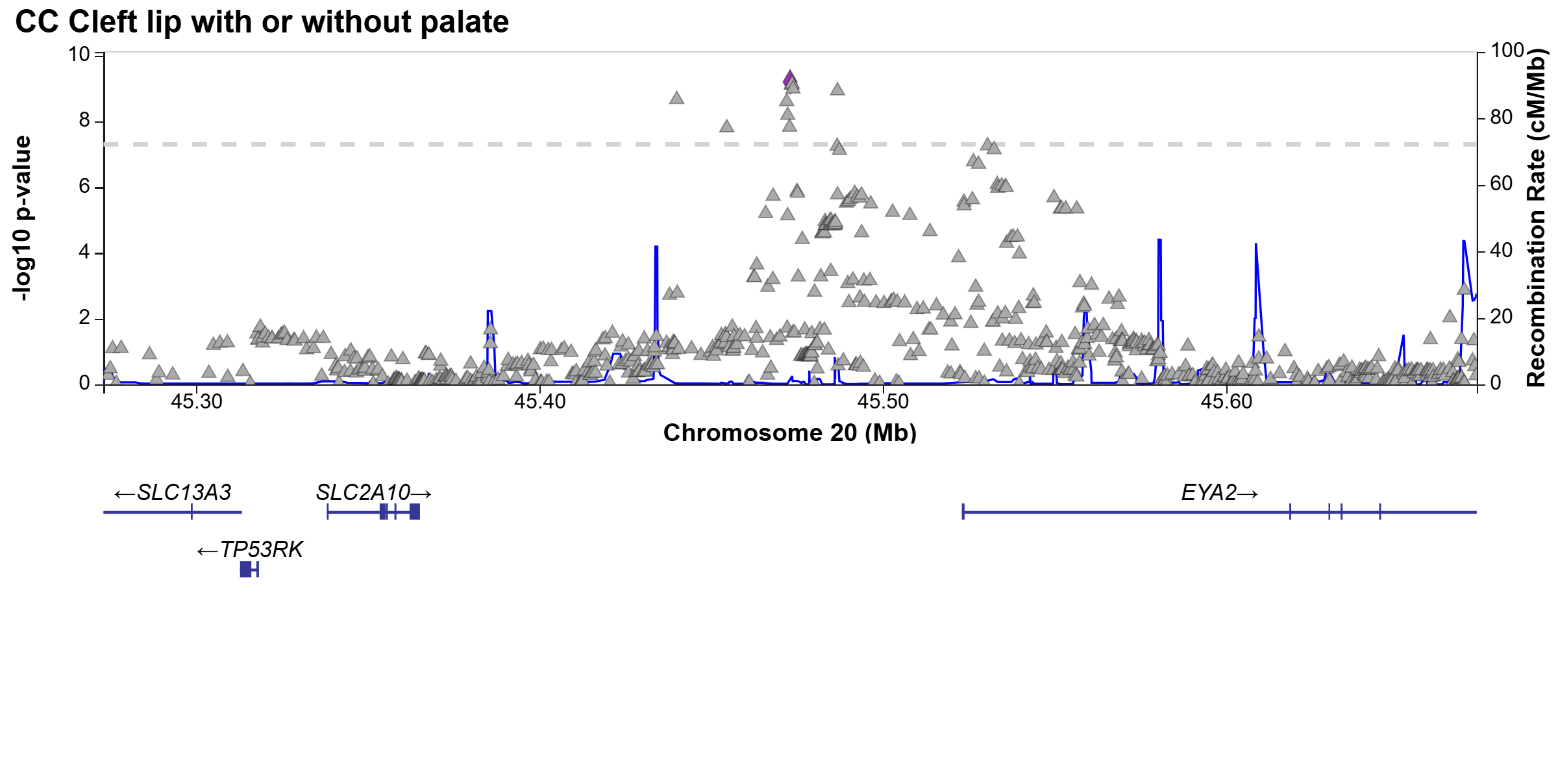


Supplementary Figure 24 – Manhattan plot – non syndromic cleft lip with or without palate


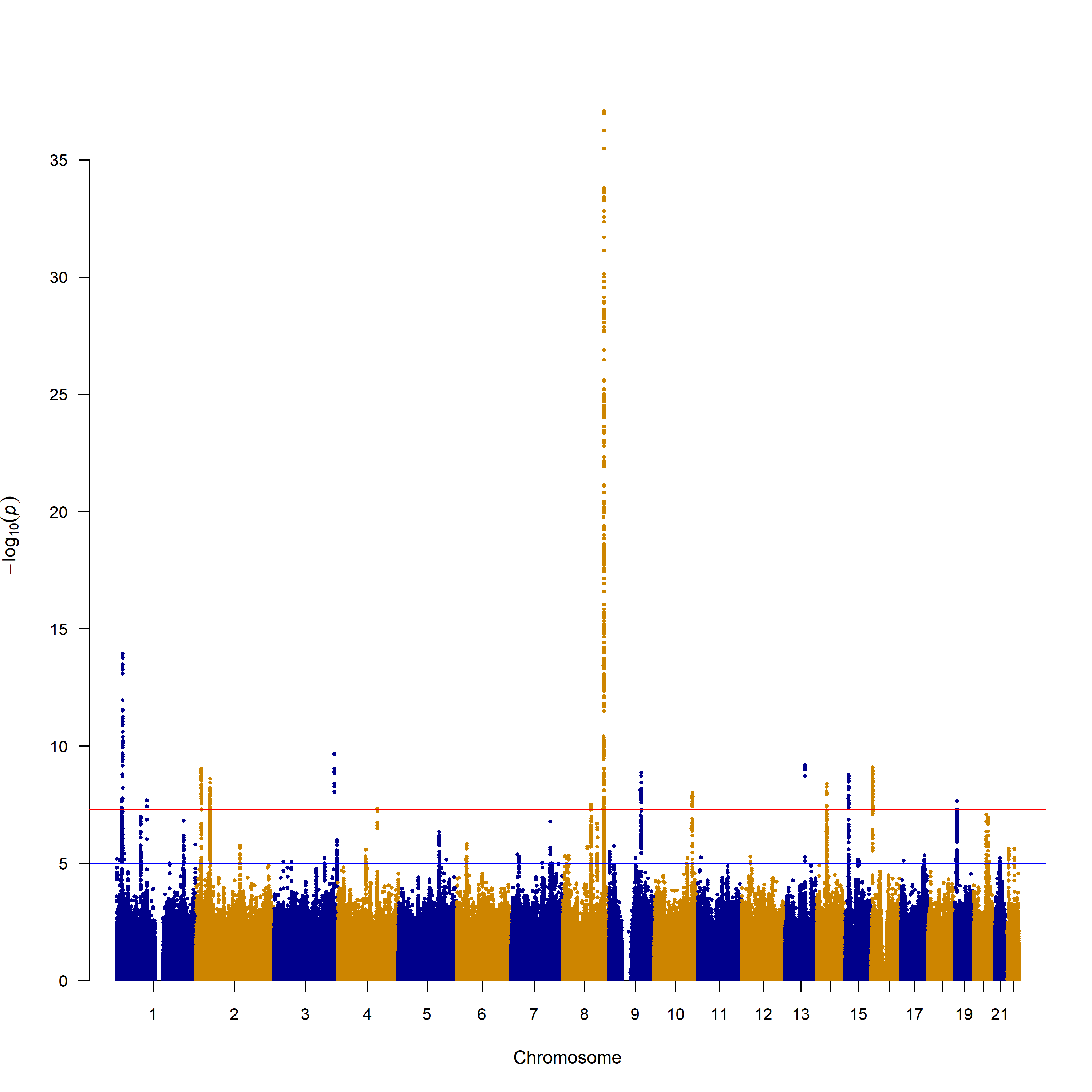


Supplementary Figure 25– Quantile-Quantile plot – non syndromic cleft lip with or without palate


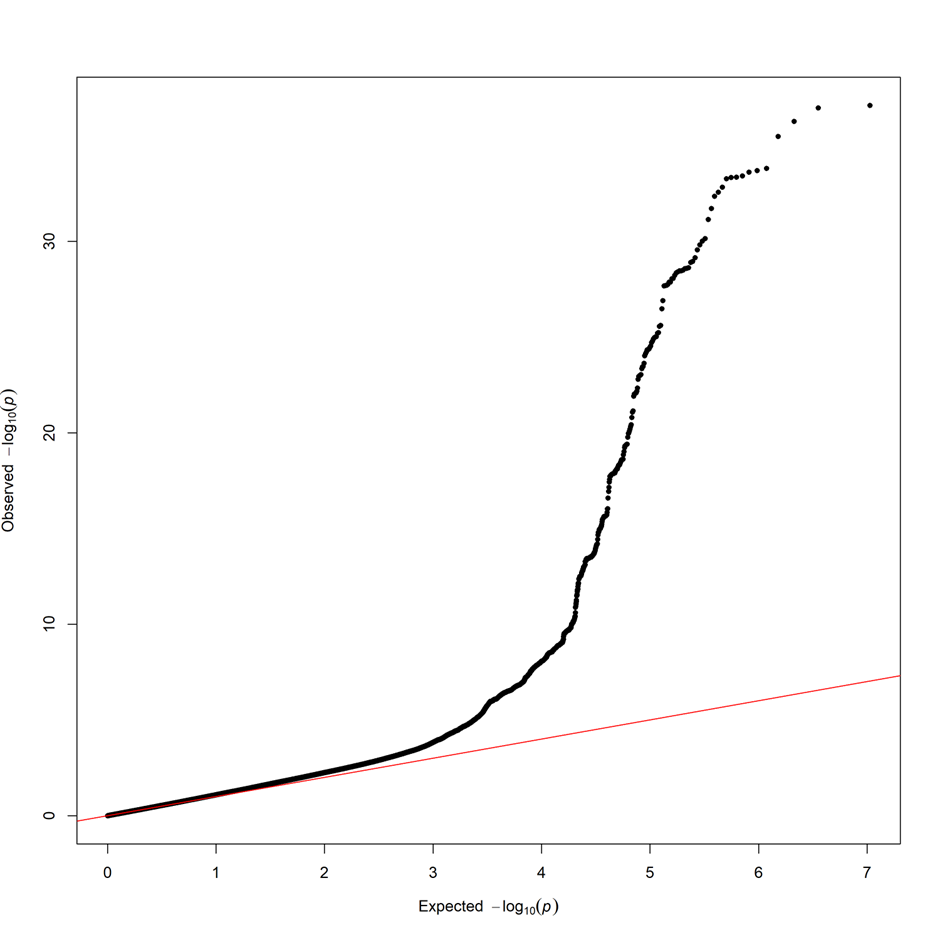


Supplementary Fig 26 - Locus Zoom Plot of region in 1p36.13 (lead SNP rs61769781) – non syndomic cleft lip with or without palate


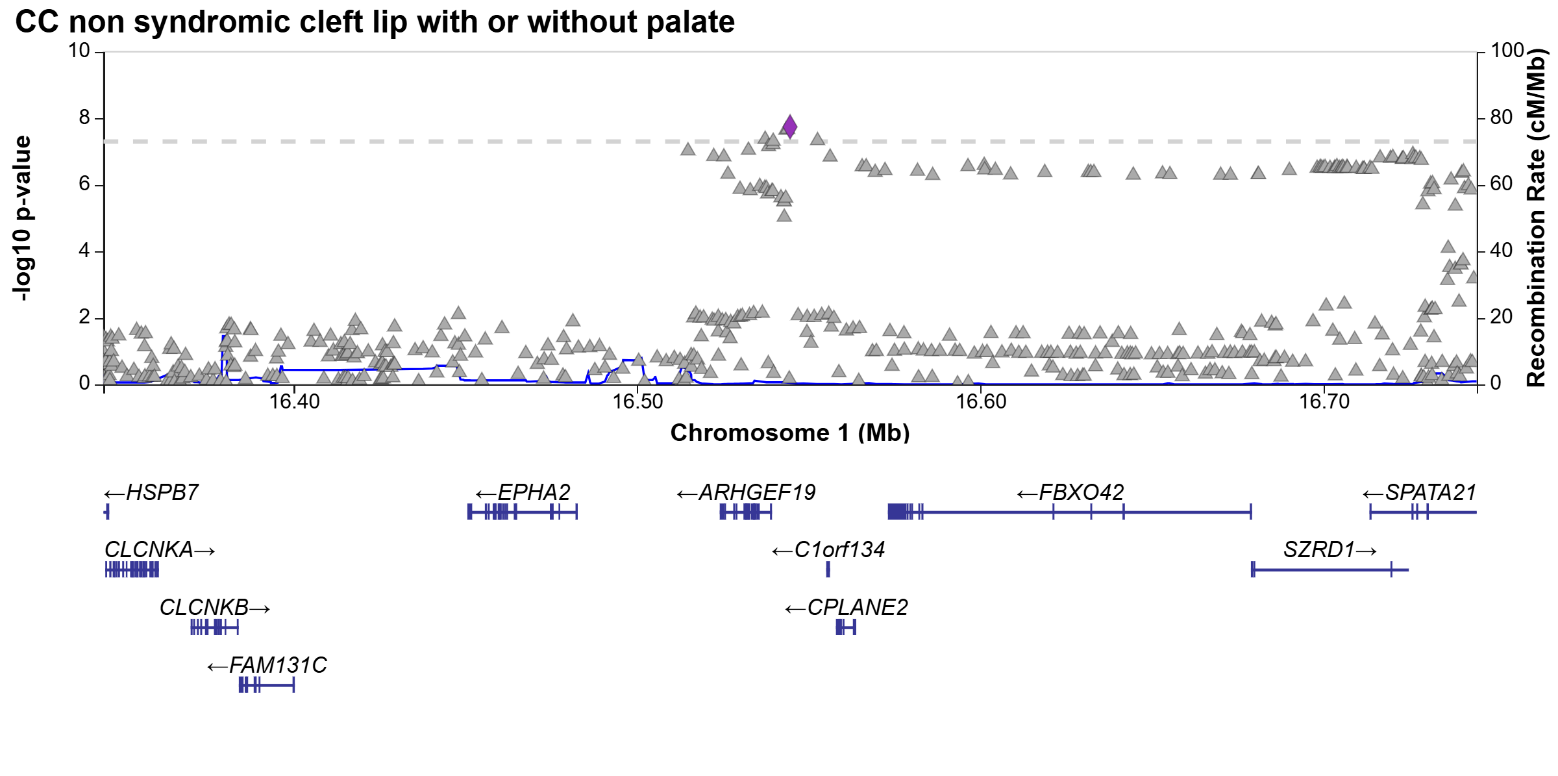


Supplementary Fig 27 - Locus Zoom Plot of region in 2p21 (lead SNP rs6741434) -non syndomic cleft lip with or without palate


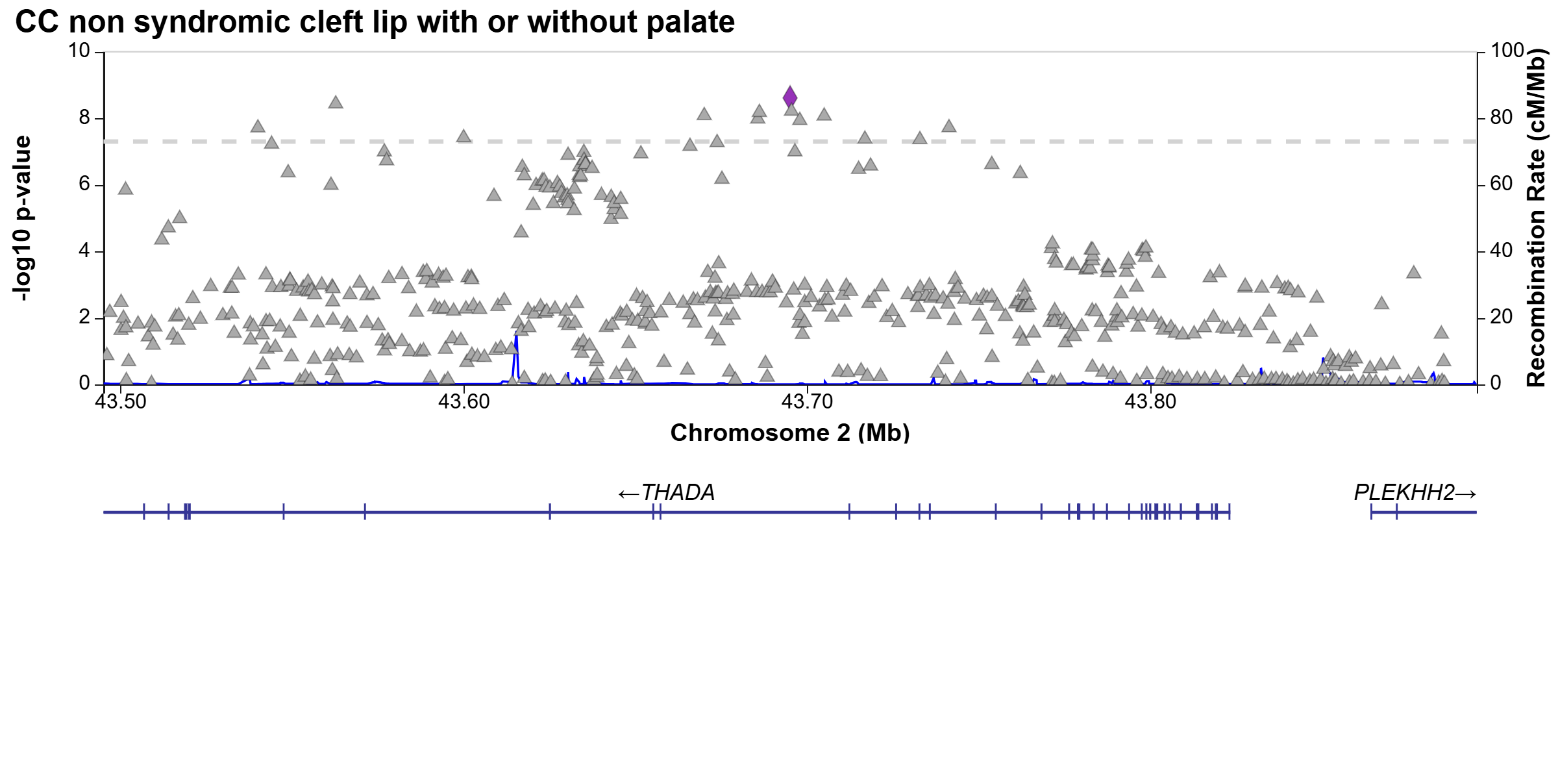


Supplementary Figure 28– Manhattan plot –cleft lip with palate


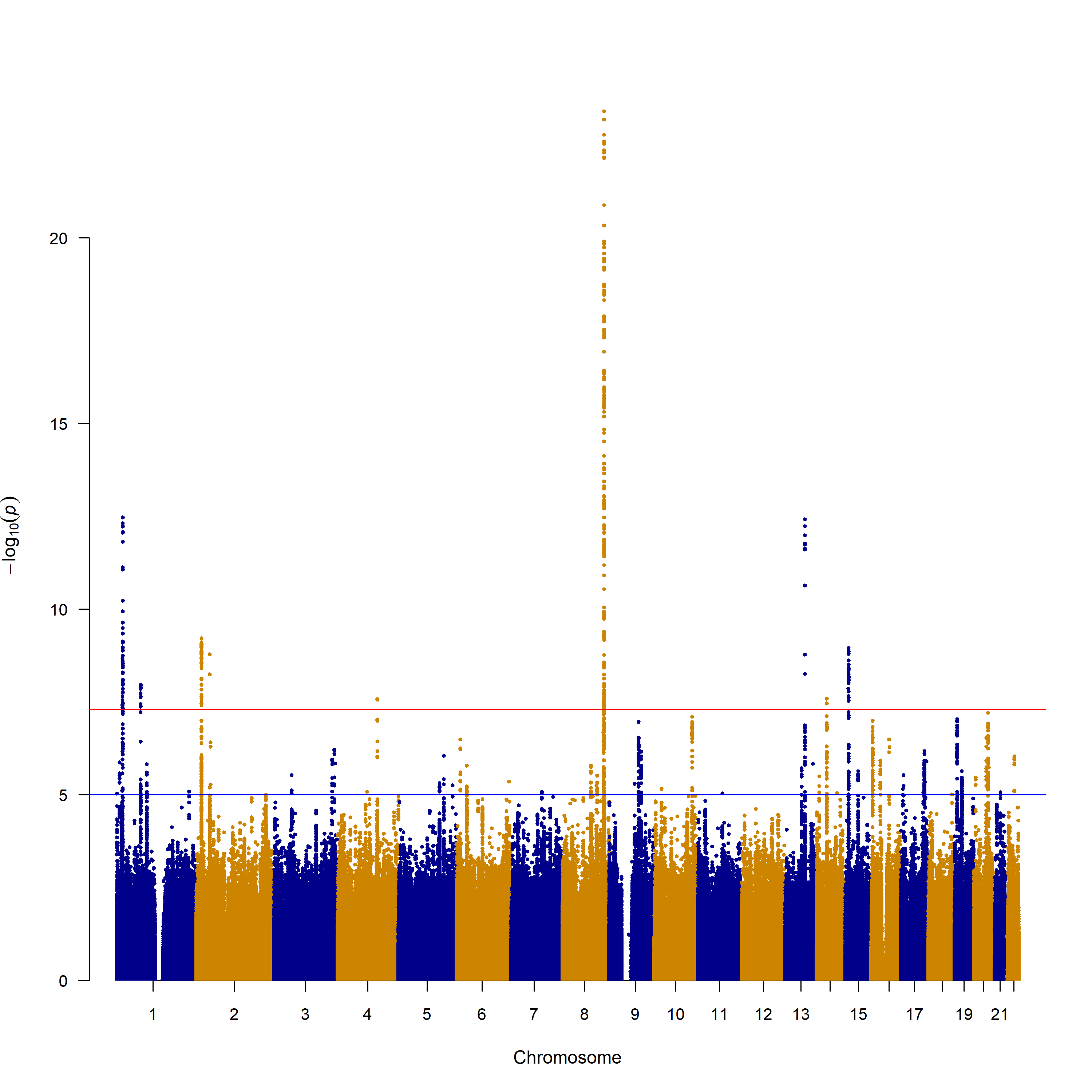


Supplementary Figure 29– Quantile-Quantile plot – cleft lip with palate
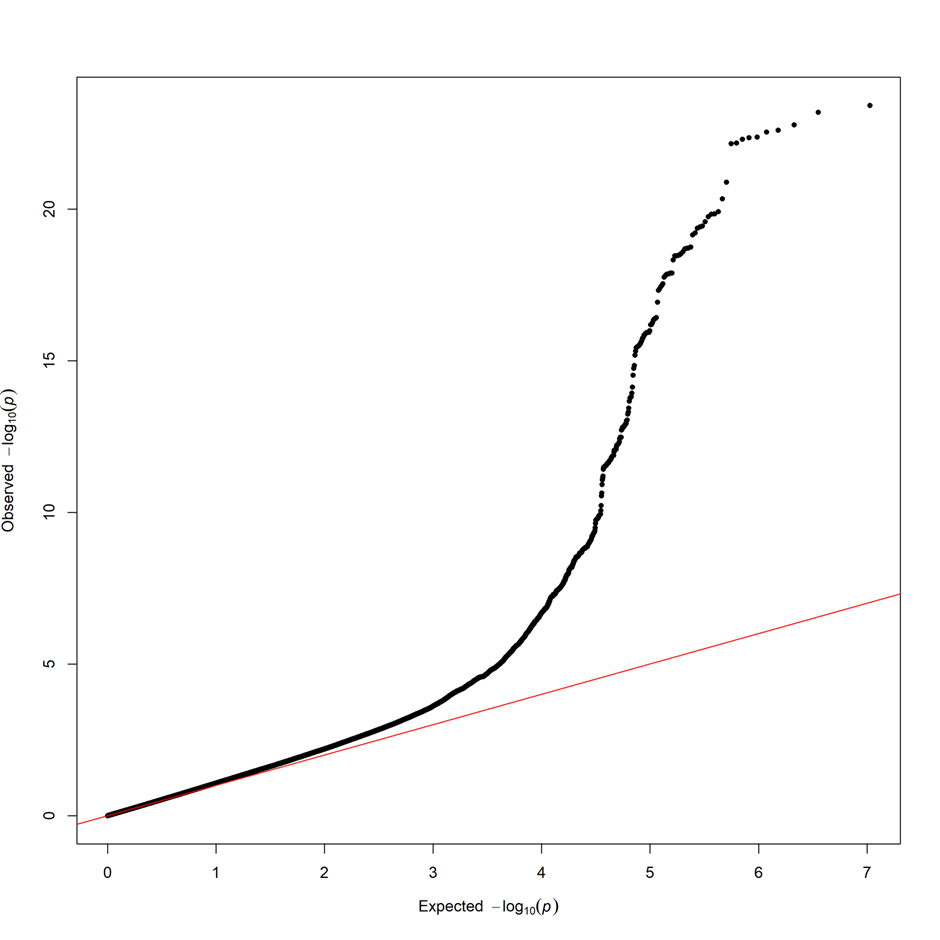


Supplementary Figure 30 - Locus Zoom Plot of region in 4q28.1 (lead SNP rs1347188)-cleft lip with palate
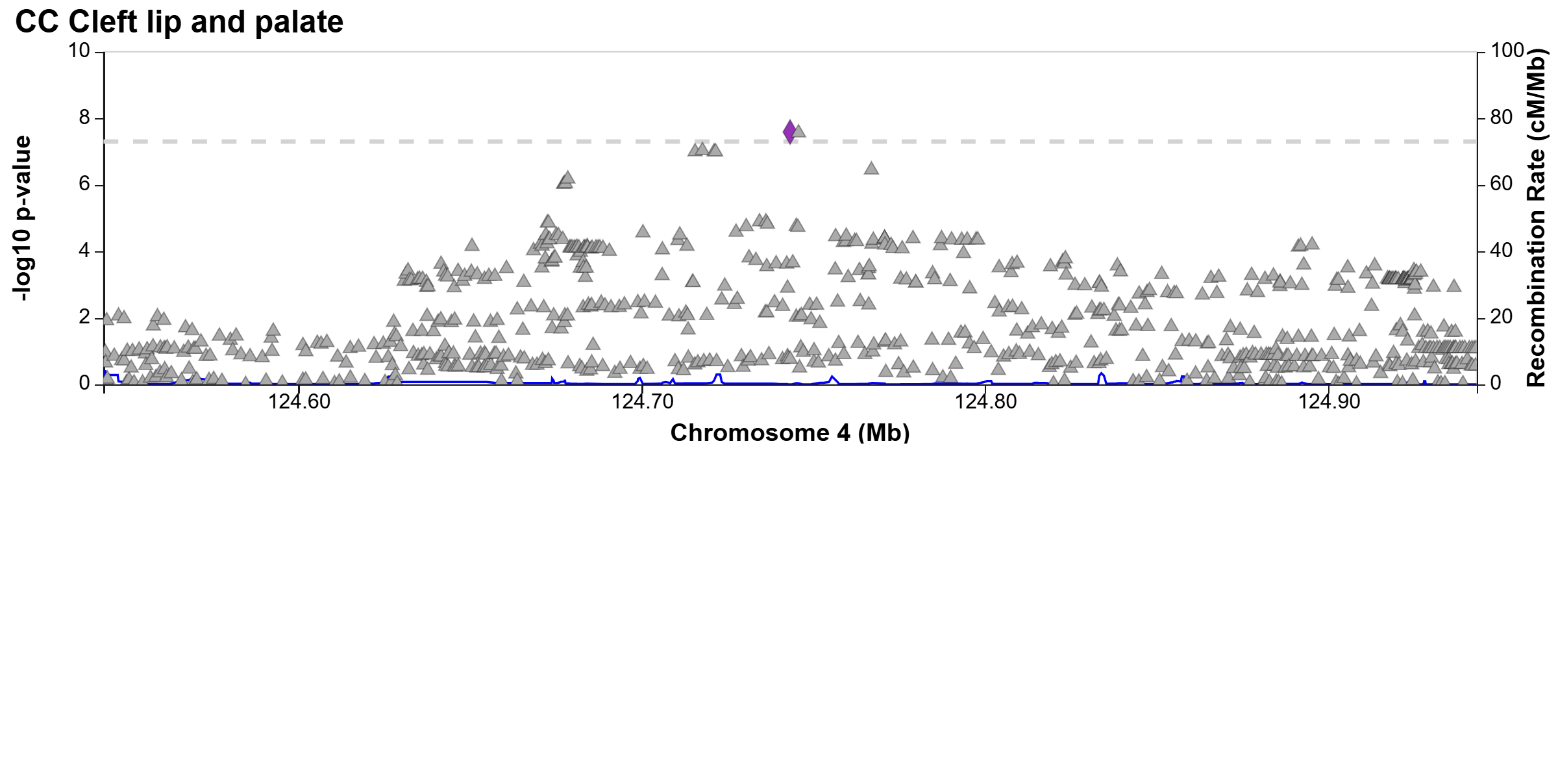
 Supplementary Figure 31 - Locus Zoom Plot of region in 13q31.1 (lead SNP rs9601323)-cleft lip with palate


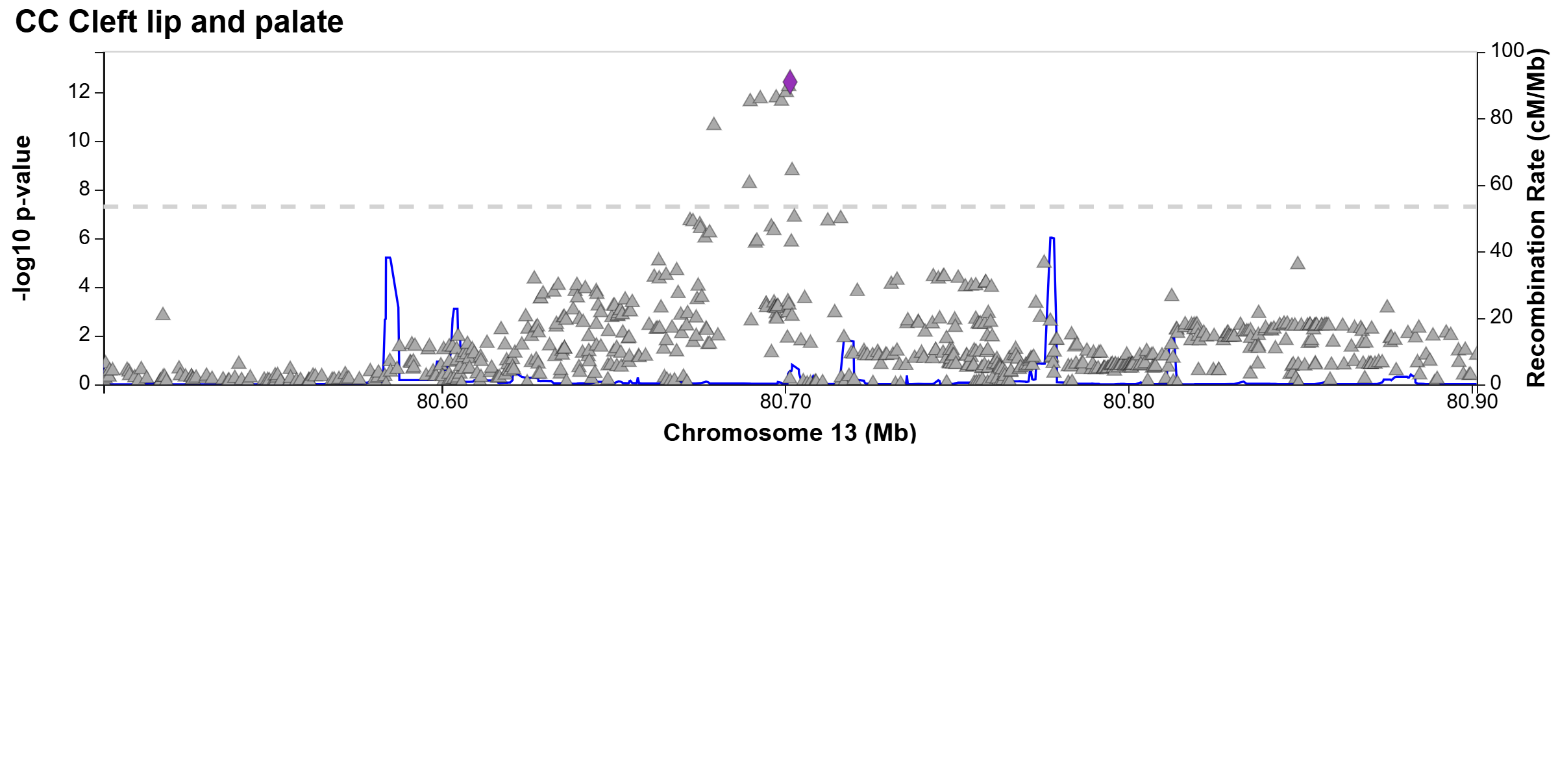


Supplementary Figure 32 – Manhattan plot –cleft lip only
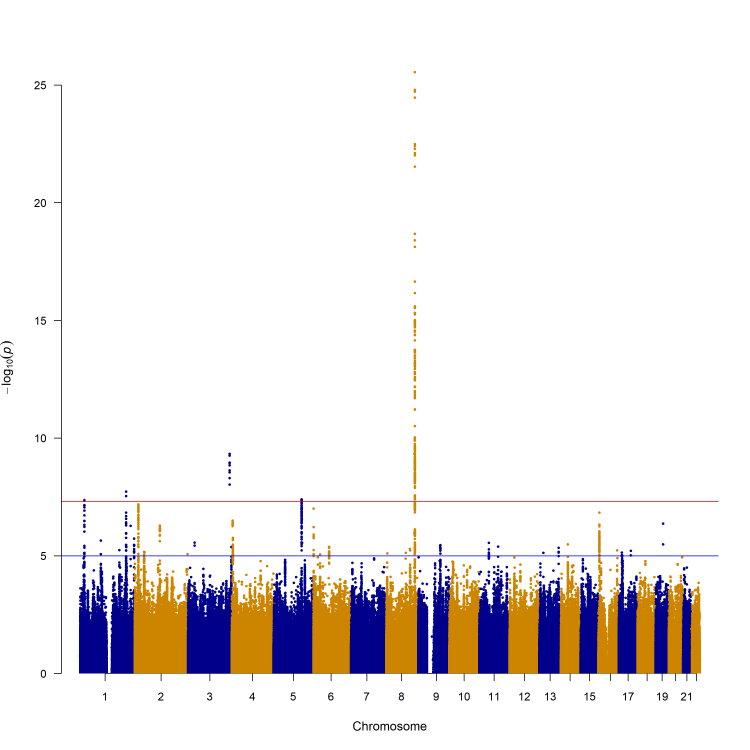


Supplementary Figure 33– Quantile-Quantile plot – cleft lip only
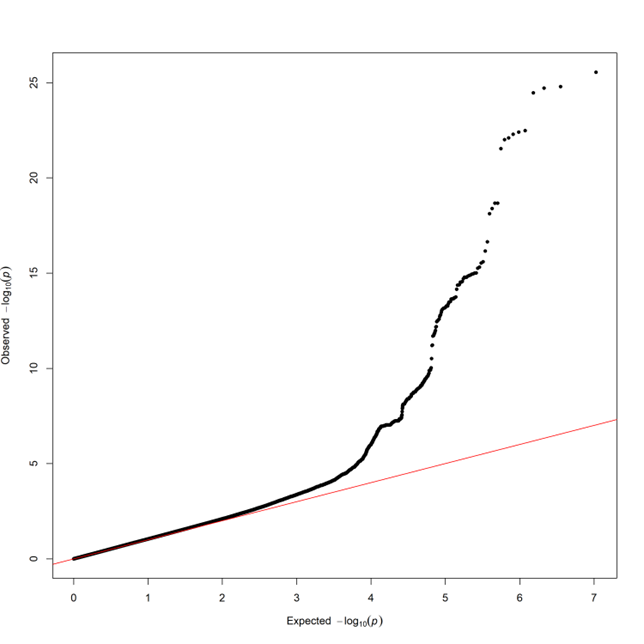


Supplementary Figure 34 - Locus Zoom Plot of region in 1q32.2 (lead SNP rs126280) - cleft lip only


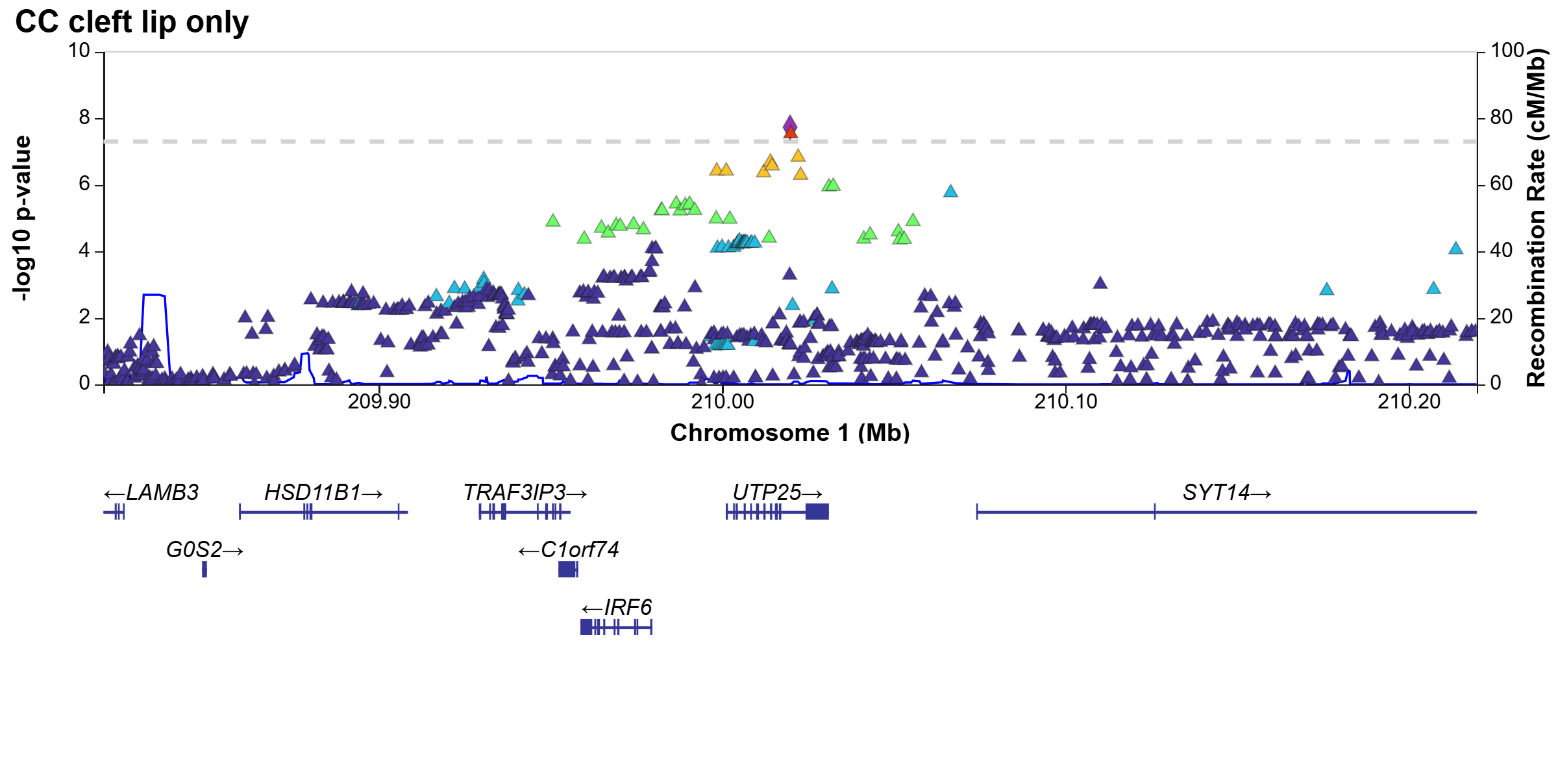


Supplementary Figure 35 - Locus Zoom Plot of region in 5q23.3 (lead SNP rs62390705)-cleft lip only


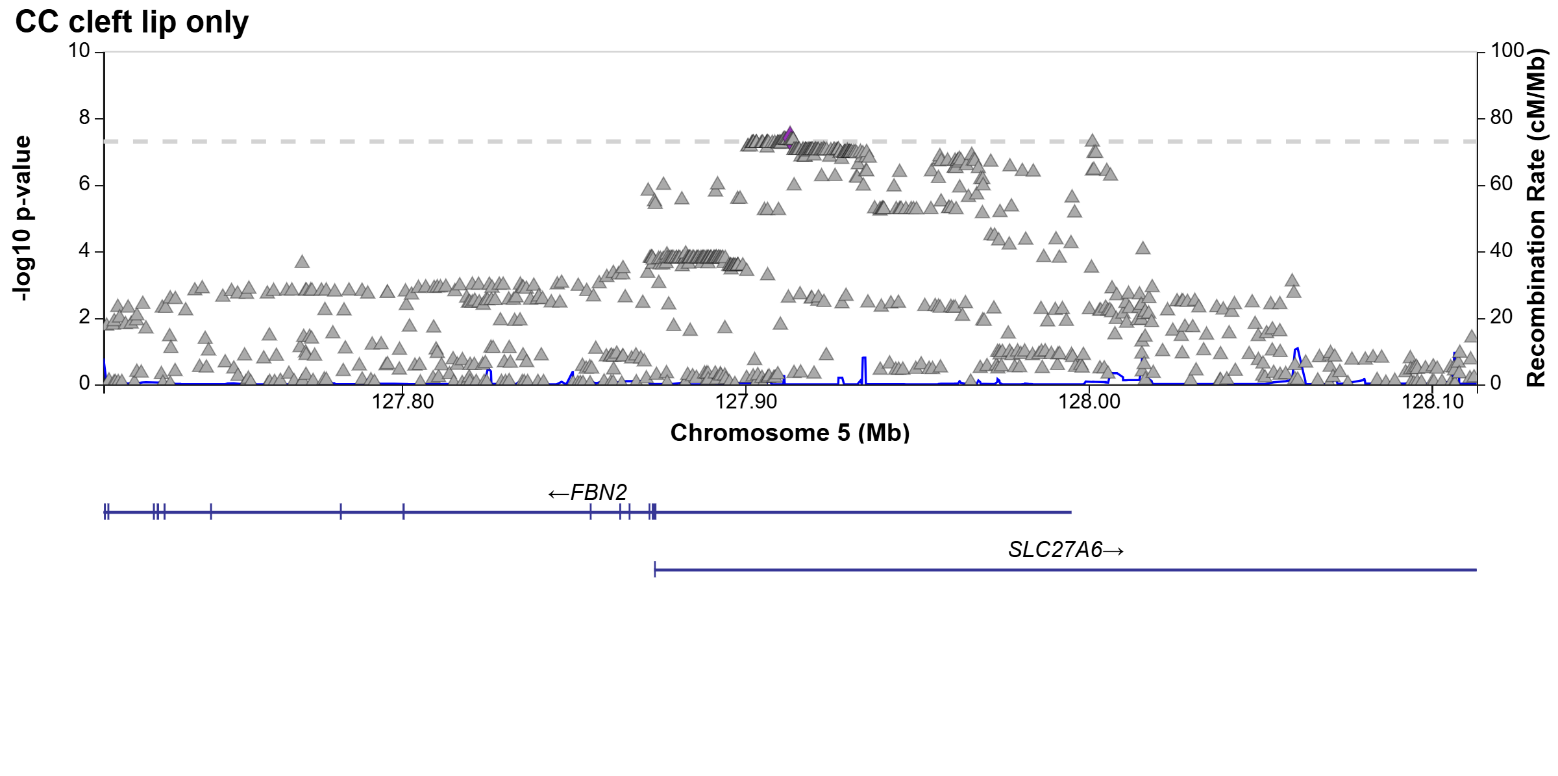


Supplementary Figure 36– Manhattan plot –cleft palate only


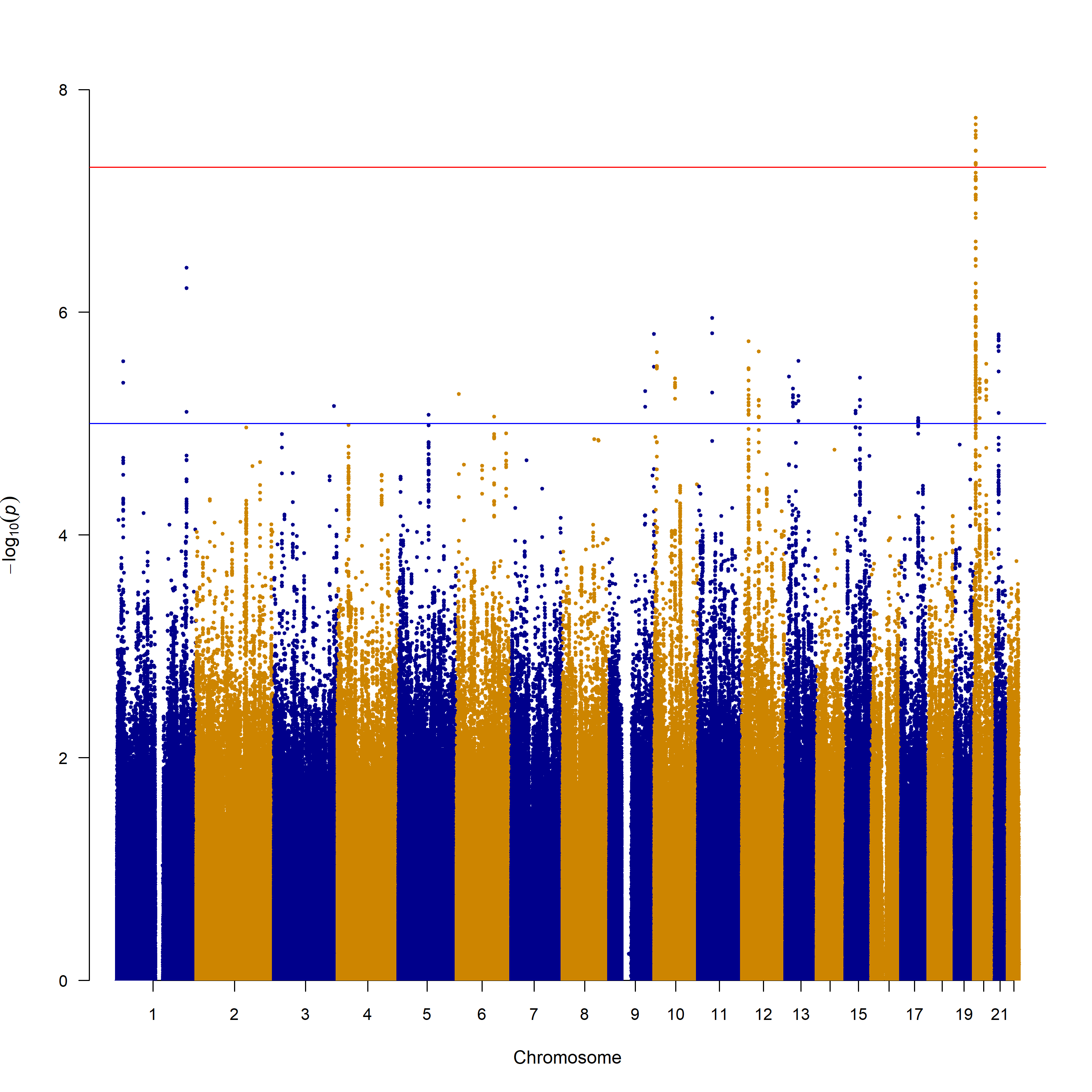


Supplementary Figure 37– Quantile-Quantile plot – cleft palate only


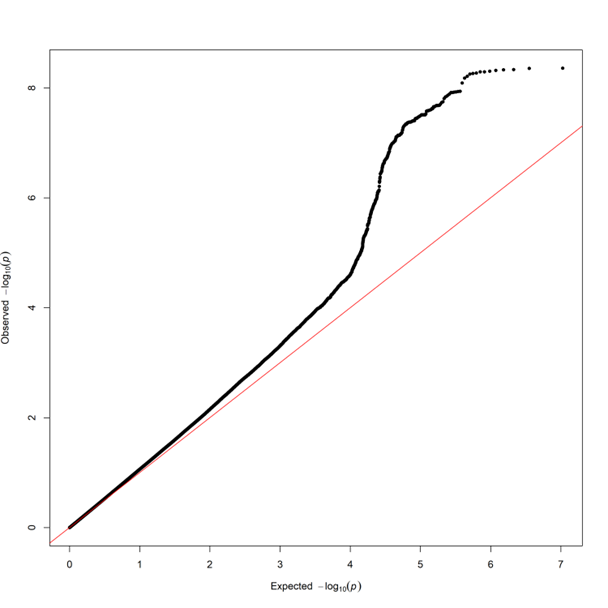


Supplementary Figure 38 - Locus Zoom Plot of region in 6p24.3 (lead SNP rs28361060)-cleft palate only


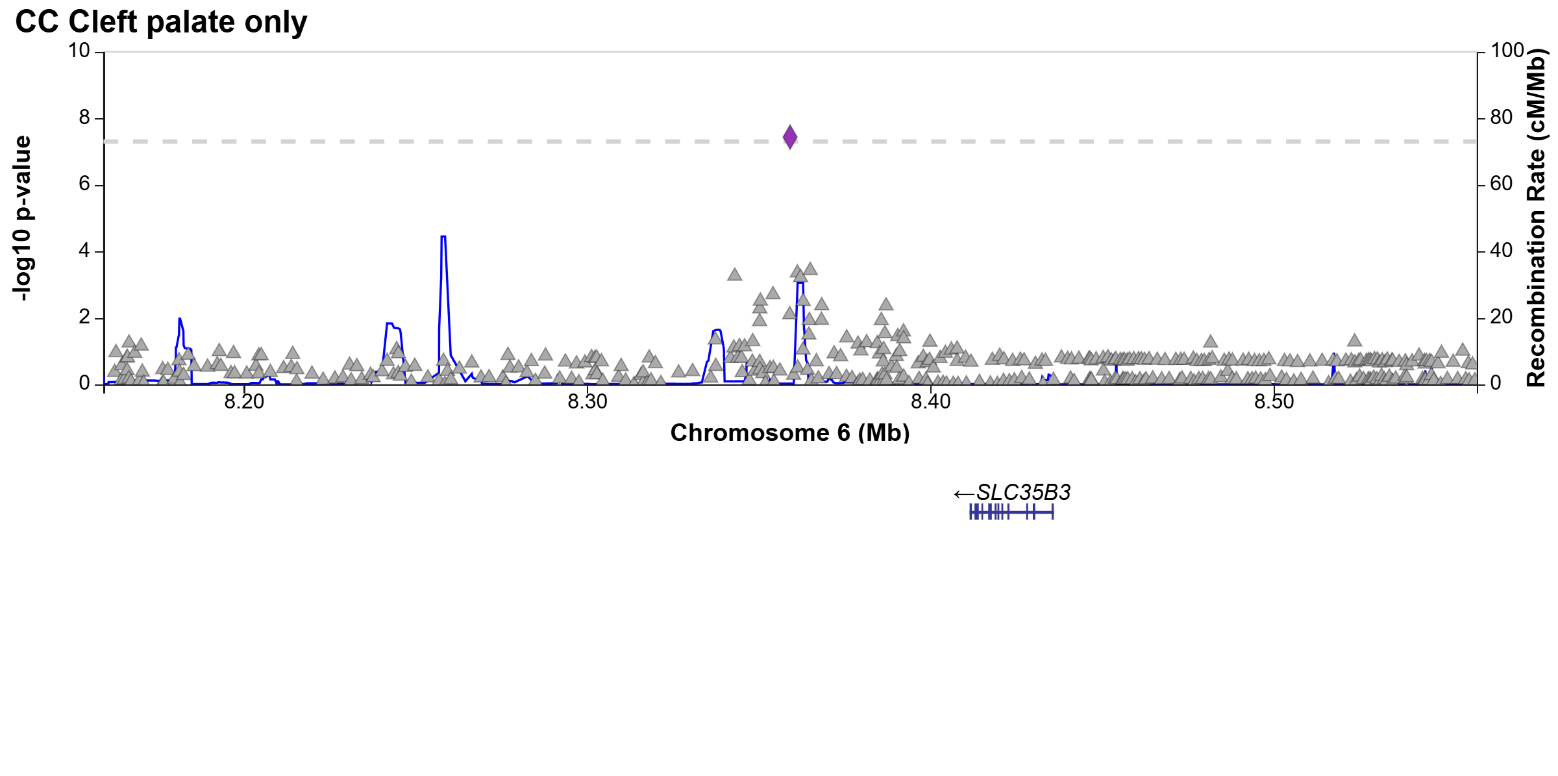
 Supplementary Figure 39 - Locus Zoom Plot of region in 20p12.3 (lead SNP rs2210119)-cleft palate only


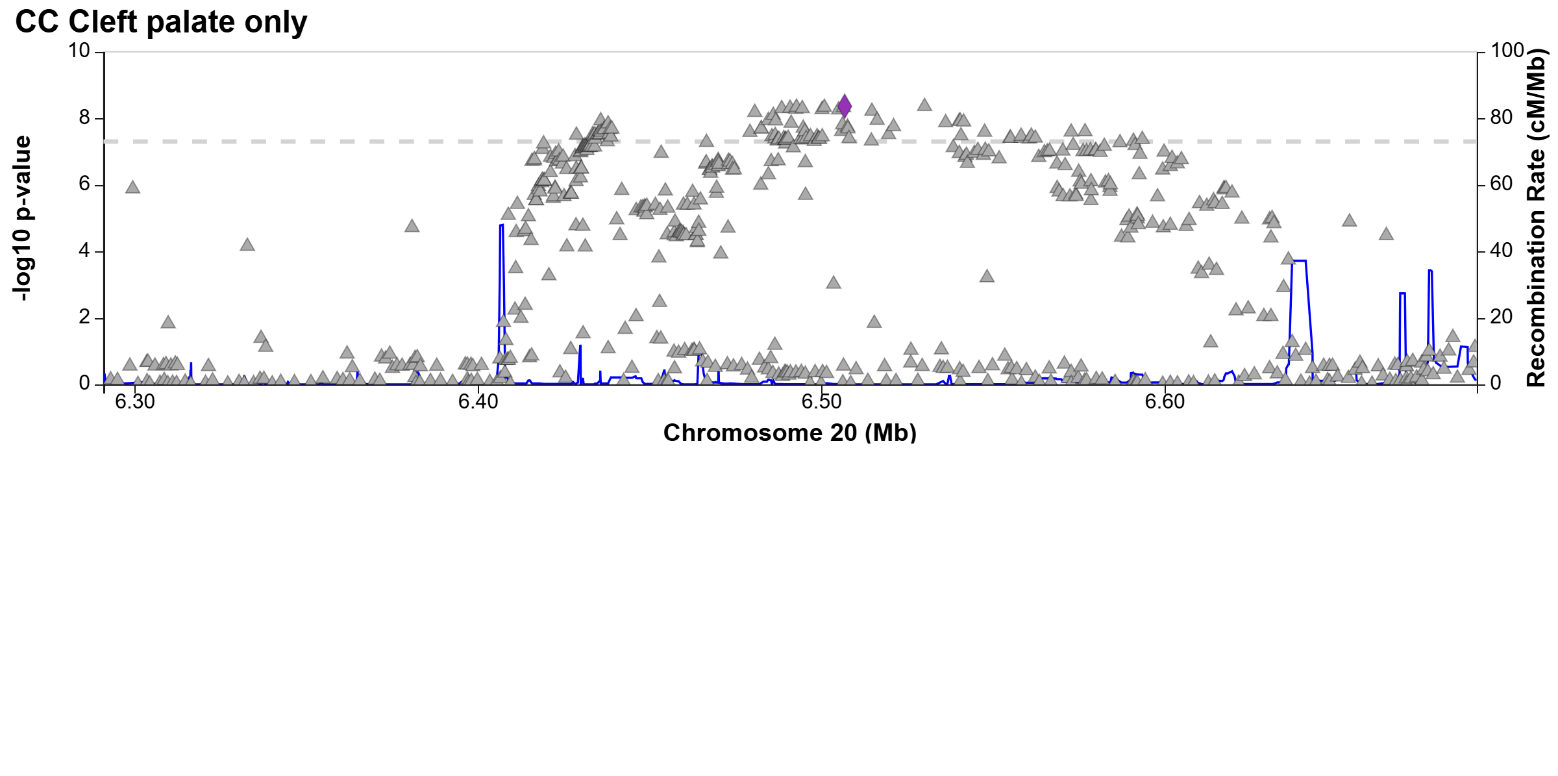


Supplementary Figure 40 – Manhattan plot – Pierre Robin sequence


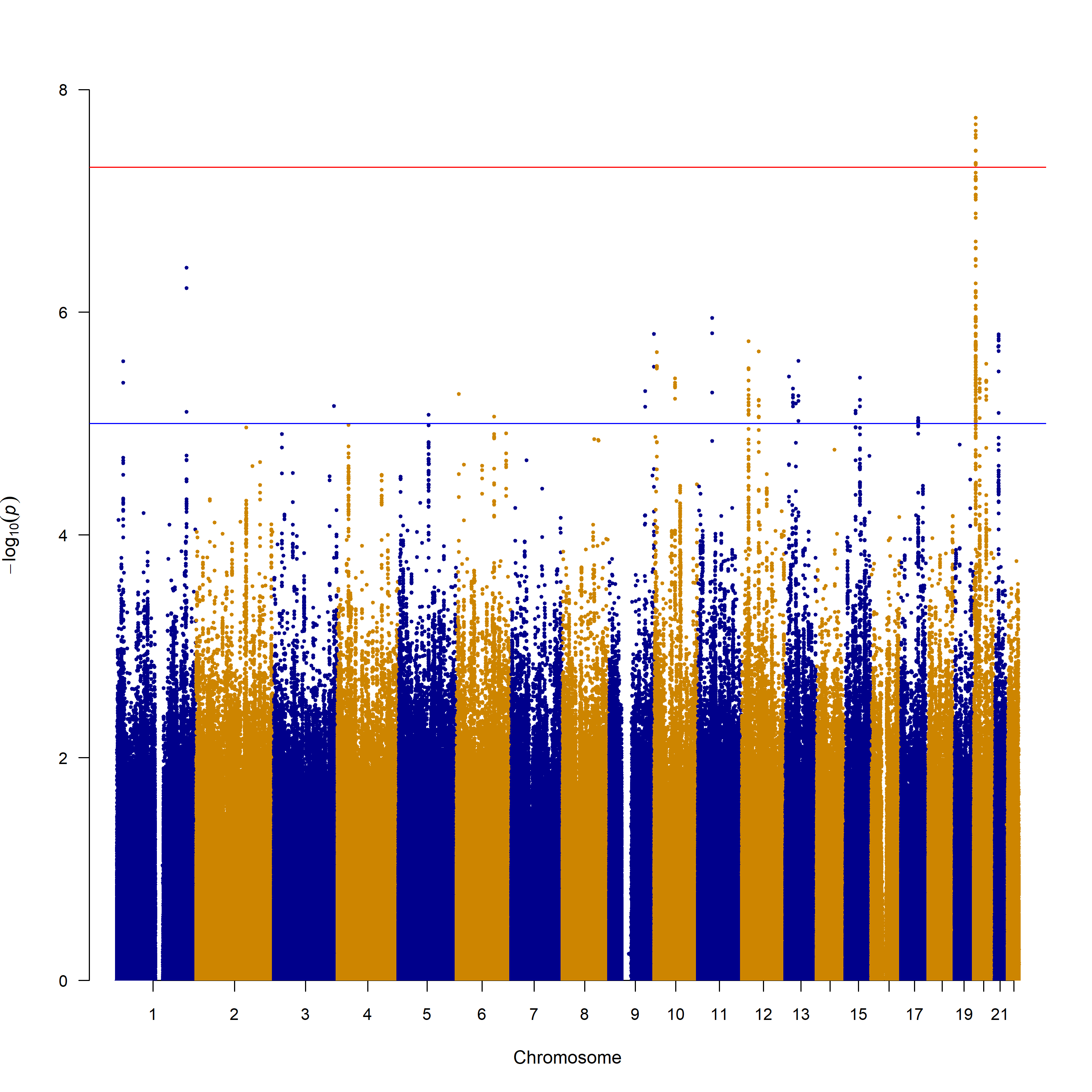


Supplementary Figure 41 –Quantile- Quantile plot – Pierre Robin sequence


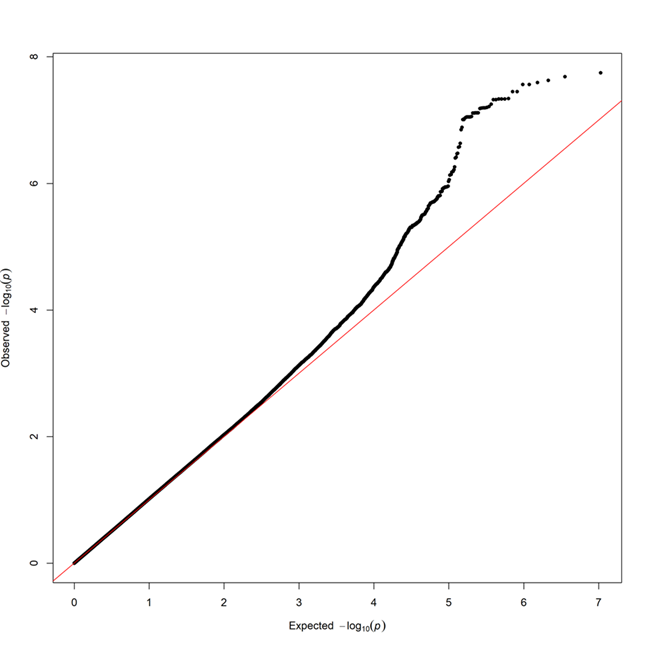


Supplementary Figure 42 – Manhattan plot – non syndromic cleft palate only without Pierre Robin sequence
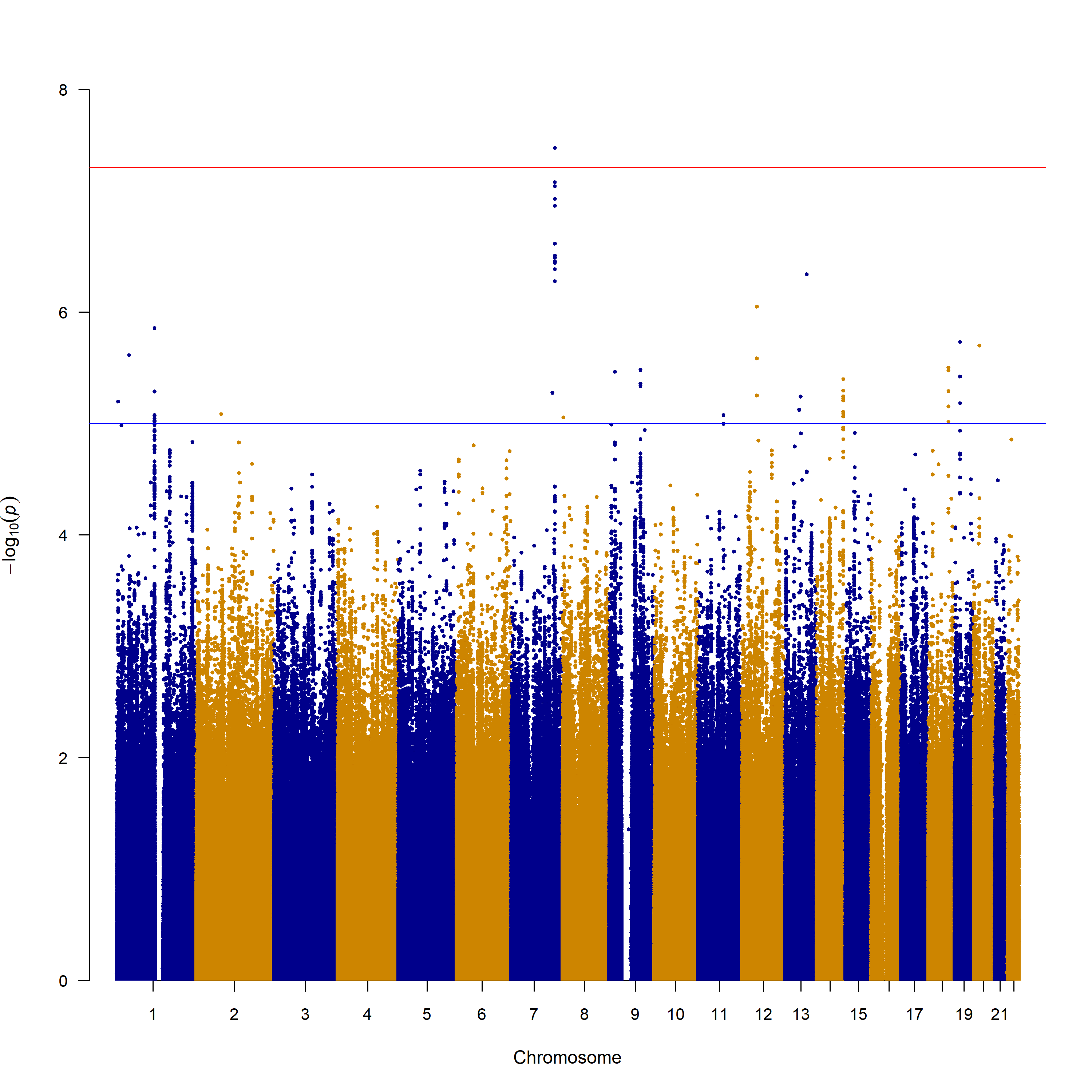


Supplementary Figure 43 – Quantile-Quantile plot – non syndromic cleft palate only without Pierre Robin Sequence


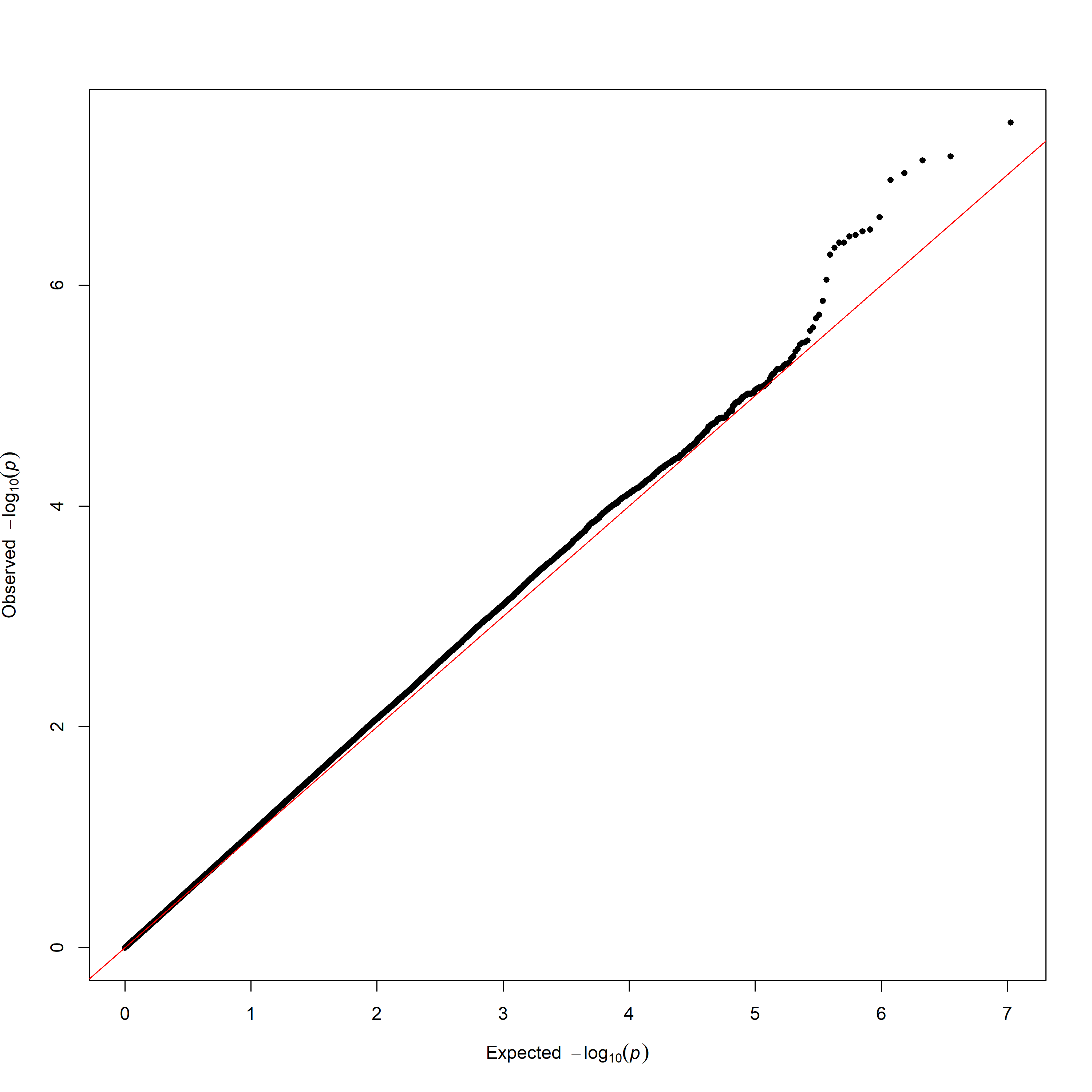


Supplementary Figure 44 - Locus Zoom Plot of region in 7q33 (lead SNP rs171168736)-non-syndromic cleft palate only


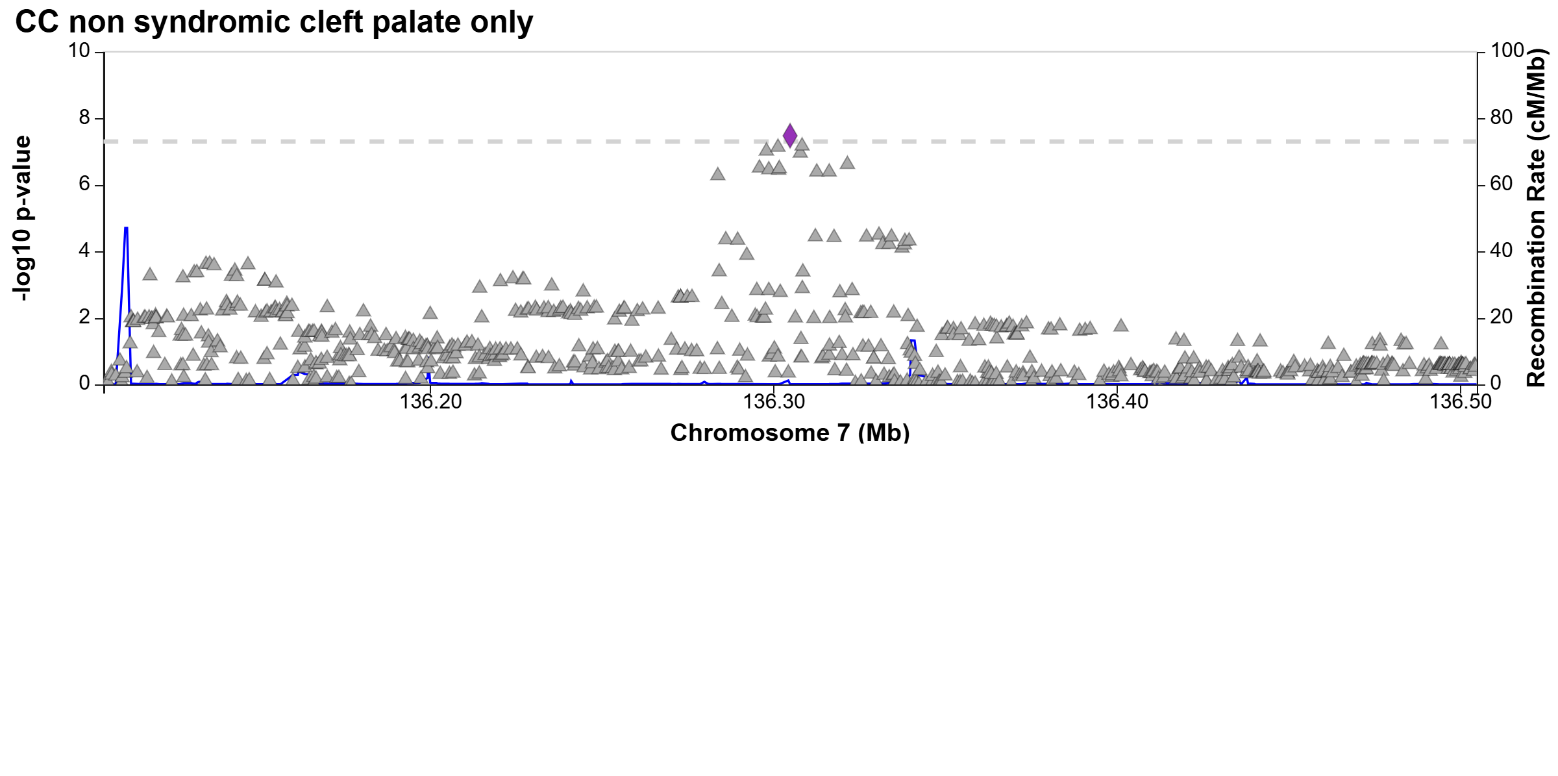

Supplement: Supplementary_Figures_revised_ddaf131 [file supplementary_figures_revised_ddaf131.docx]
